# Rates of compliance and adherence to high-intensity interval training: a systematic review and Meta-analyses

**DOI:** 10.1186/s12966-023-01535-w

**Published:** 2023-11-21

**Authors:** Alexandre Santos, Kyra Braaten, Megan MacPherson, Diego Vasconcellos, Mathew Vis-Dunbar, Chris Lonsdale, David Lubans, Mary E. Jung

**Affiliations:** 1 Faculty of Health and Social Development, University of British Columbia – Okanagan Campus, Kelowna, British Columbia Canada; 2 Institute for Positive Psychology & Education, Australian Catholic University, Melbourne, Victoria Australia; 3 Library, University of British Columbia – Okanagan Campus, Kelowna, British Columbia Canada; 4 School of Education, University of Newcastle, Newcastle, New South Wales Australia; 5 Hunter Medical Research Institute, New Lambton Heights, NSW 2305 Australia; 6 Faculty of Sport and Health Sciences, University of Jyväskylä, Jyväskylä, Finland

**Keywords:** High-intensity interval training, Moderate-intensity continuous training, Compliance, Adherence, Systematic review, meta-analysis

## Abstract

**Background:**

To determine rates of compliance (i.e., supervised intervention attendance) and adherence (i.e., unsupervised physical activity completion) to high-intensity interval training (HIIT) among insufficiently active adults and adults with a medical condition, and determine whether compliance and adherence rates were different between HIIT and moderate-intensity continuous training (MICT).

**Methods:**

Articles on adults in a HIIT intervention and who were either insufficiently active or had a medical condition were included. MEDLINE, EMBASE, PsychINFO, SPORTDiscus, CINAHL, and Web of Science were searched. Article screening and data extraction were completed by two independent reviewers. Risk of bias was assessed using RoB 2.0 or ROBINS-I. Meta-analyses were conducted to discern differences in compliance and adherence between HIIT vs. MICT. Sensitivity analyses, publication bias, sub-group analyses, and quality appraisal were conducted for each meta-analysis.

**Results:**

One hundred eighty-eight unique studies were included (*n* = 8928 participants). Compliance to HIIT interventions averaged 89.4% (*SD*:11.8%), while adherence to HIIT averaged 63% (*SD*: 21.1%). Compliance and adherence to MICT averaged 92.5% (*SD*:10.6%) and 68.2% (*SD*:16.2%), respectively. Based on 65 studies included in the meta-analysis, compliance rates were not different between supervised HIIT and MICT interventions [Hedge’s *g* = 0.015 (95%CI: − 0.088–0.118), *p* = .78]. Results were robust and low risk of publication bias was detected. No differences were detected based on sub-group analyses comparing medical conditions or risk of bias of studies. Quality of the evidence was rated as moderate over concerns in the directness of the evidence. Based on 10 studies, adherence rates were not different between unsupervised HIIT and MICT interventions [Hedge’s *g* = − 0.313 (95%CI: − 0.681–0.056), *p* = .096]. Sub-group analysis points to differences in adherence rates dependent on the method of outcome measurement. Adherence results should be interpreted with caution due to very low quality of evidence.

**Conclusions:**

Compliance to HIIT and MICT was high among insufficiently active adults and adults with a medical condition. Adherence to HIIT and MICT was relatively moderate, although there was high heterogeneity and very low quality of evidence. Further research should take into consideration exercise protocols employed, methods of outcome measurement, and measurement timepoints.

**Registration:**

This review was registered in the PROSPERO database and given the identifier CRD42019103313.

**Supplementary Information:**

The online version contains supplementary material available at 10.1186/s12966-023-01535-w.

## Background

Physical inactivity is a prominent issue worldwide with an estimated 27.5% (95% CI: 25.0–32.2%) of the global population not meeting recommended physical activity guidelines of 75–150 minutes of moderate-to-vigorous physical activity (MVPA) per week [1, 2]. Individuals who are insufficiently active are at higher risk of developing non-communicable chronic diseases such as coronary heart disease, type 2 diabetes, and certain forms of cancer, and an estimated 9% of deaths are attributable to physical inactivity [3]. In contrast, increasing physical activity can lead to an abundance of benefits, some of which include primary and secondary prevention of chronic disease [4], improved cardiorespiratory fitness [5], improved psychological well-being [6], and a reduction in all-cause mortality [7].

The promotion of physical activity through supervised exercise interventions is a common strategy used in healthy and clinical populations, and such interventions have generally shown favorable physiological outcomes [8–11]. Of interest, high-intensity interval training (HIIT) is a type of aerobic exercise that has grown in popularity in recent years [12] as an alternative to more traditional forms of exercise such as moderate-intensity continuous training (MICT). MICT is defined as a continuous effort of at least 10 minutes at a moderate intensity (i.e., 64–76% of maximum heart rate [HRmax]) [13]. HIIT is broadly defined as short bursts of high-intensity exercise (> 80% HRmax) interspersed with periods of rest or light active recovery [14] and can be manipulated into an infinite number of lengths and iterations. Compared to MICT, HIIT may be more time-efficient, and research suggests there are no or small significant differences between the two regarding improvements of physiological markers such as cardiorespiratory fitness [14, 15], vascular function [16], body composition [17], and glycated hemoglobin profiles [18]. Similarly, Oliveira and colleagues [19] have reported comparable rates of perceived exercise enjoyment and affective responses to exercise, although this review has been recently critiqued on the basis of using a single summary statistic to examine enjoyment and affect [20].

*Compliance,* conceptualized as attendance to supervised HIIT and/or MICT sessions, is a key metric for the success of exercise interventions. Systematic reviews have demonstrated generally high attendance rates to supervised HIIT and/or MICT sessions, with Weston and colleagues [14] reporting attendance rates > 85% for six studies focusing on individuals with cardiometabolic disease, and De Nardi and colleagues [18] reporting attendance rates between 70 and 90% in four studies on individuals with prediabetes or type 2 diabetes. The small number of studies synthesized and the specificity of the target populations in these systematic reviews hinder the generalizability of results to a broader population of individuals who are insufficiently active or present with varying medical conditions. Furthermore, the translation of exercise compliance in supervised exercise interventions to unsupervised *adherence*, originally conceptualized as any engagement in real-world physical activity, is not well-known. The need for such synthesis has already been alluded to by Oliveira and colleagues [19], who document that long-term studies are needed to clarify the applicability of HIIT interventions for exercise adherence.

There is debate in the literature as to whether HIIT is a more feasible type of exercise in pragmatic settings compared to MICT [21]. Some argue that HIIT is an enjoyable and time efficient exercise modality, increasing appeal to a general population where time is the most prominent self-reported reason for non-engagement in physical activity [22, 23]. Others argue that HIIT elicits more negative affective responses compared to MICT, which may result in subsequent disengagement from exercise [24]. Preliminary evidence on adherence to HIIT/MICT interventions in real-world unsupervised settings have found mixed results which may be due to differing methods of measuring exercise in free-living conditions. In 2014, Lunt and colleagues [25] found modest adherence rates to a HIIT program in a real-world setting for adults who were overweight and inactive, stating that non-adherence to the exercise program was likely the main reason for small observed changes in cardiorespiratory fitness compared to previous studies. A more recent study by Jung and colleagues [26] showed that after a brief supervised HIIT/MICT program, individuals who were low-active increased weekly MVPA in unsupervised, real-world settings over the next 12 months compared to baseline values measured via accelerometry. A recent literature search by Ekkekakis and Biddle [27] revealed no apparent differences in long-term physical activity adherence between HIIT and MICT protocols. However, this study focused only on long-term adherence and included eight trials with a minimum 12-month follow-up, with no meta-analysis conducted. Given the debate of whether HIIT is a viable type of exercise in real-world settings for individuals who are insufficiently active, coupled with preliminary mixed findings and apparent heterogeneous program designs and methods of measuring physical activity adherence, there is a need for a synthesis of the evidence on physical activity adherence following HIIT programs [19]. The results of such synthesis have important implications on future intervention design and physical activity recommendations.

Compliance to HIIT interventions among individuals who are insufficiently active or present with a medical condition are not known. Furthermore, there is no quantitative synthesis to determine whether there is a difference in compliance rates between HIIT and MICT interventions in these populations. In addition, few studies have examined physical activity adherence following supervised HIIT or MICT interventions. It is also not known whether physical activity adherence is statistically different dependent on the exercise modality engaged in. As such, the primary and secondary purposes of our review were to:

1. Synthesize compliance and adherence rates to HIIT interventions for adults who are insufficiently active or present with a medical condition, and
2. Determine whether compliance and adherence rates differ between HIIT and MICT interventions for adults who are insufficiently active or present with a medical condition.

Based on previous results [14, 18], we hypothesized that compliance to supervised HIIT and MICT interventions would be comparable and relatively high. Considering high rates of physical inactivity [2], conflicting perspectives on the feasibility of HIIT in real-world scenarios [21–24], and mixed preliminary evidence [25–27], we hypothesized that physical activity adherence rates following supervised interventions would be variable between HIIT and MICT dependent on intervention type and methods of physical activity measurement.

## Methods

The reporting of this systematic review follows the 2020 Preferred Reporting Items for Systematic Reviews and Meta-Analyses (PRISMA) statement [28] and the completed PRISMA 2020 checklist can be found as Additional File 1. This review was registered in the PROSPERO database on March 8th, 2019 and given the identifier CRD42019103313. The protocol of this systematic review has been published elsewhere [29]. Extracted data from included studies, data used for analyses, and the analytic code used for quantitative synthesis of the data are available by the study authors upon reasonable request. We have no competing interests or financial support associated with this study to declare.

### Eligibility criteria

Inclusion of studies in this systematic review was based on pre-specified criteria relating to the domains of the PICOS framework [30] and the full details of inclusion and exclusion criteria can be found in Table 1. Briefly, studies were considered eligible if they met the following criteria: Population – human participants between the average ages of 18–65 years who were insufficiently active (i.e., not meeting recommended physical activity guidelines) [1] or defined as presenting with a medical condition; Intervention – a supervised or unsupervised HIIT intervention; Comparator – studies that included a MICT intervention and measured compliance or adherence for these participants were used as comparator groups; Outcomes – a quantifiable measure of compliance or adherence to the HIIT intervention; Study Type – full-text, peer-reviewed, primary research articles. Both randomized and nonrandomized experimental studies were included in an attempt to decrease publication bias.

**Table 1:** Pre-specified PICOs domains for eligibility criteria

Domain	Inclusion Criteria
Population	• 18–65 years of age • Insufficiently active^a^ • If activity not specified, diagnosed with a co-morbidity • Not an animal study
Intervention	• Supervised or unsupervised HIIT intervention^b^
Comparator	• Supervised or unsupervised MICT intervention^c^
Outcomes	• Quantifiable measure of compliance to supervised HIIT program^d^ • Quantifiable measure of adherence to unsupervised exercise following a supervised HIIT program^e^
Study Type	• Full-text available • Peer-reviewed • Observational studies or variations thereof • Randomized controlled trials or variations thereof • Not qualitative, secondary research, grey literature, published protocol, or published abstract^f^

^a^ Insufficiently active is defined as not meeting current physical activity guidelines of 75 minutes of vigorous intensity exercise or 150 minutes of moderate intensity exercise per week [1]

^b^ *HIIT* high-intensity interval training; defined as alternating short bursts of high-intensity (> 80% maximum/peak heart rate or equivalent) exercise with recovery periods or light exercise [14]

^c^ *MICT* moderate-intensity interval training; defined as achieving between ~ 64 and 76% of maximum heart rate or equivalent for a continuous period of at least 10 minutes [13]

^d^ Compliance measured as the frequency of attendance to supervised exercise sessions, either as number of sessions or percentage of sessions attended

^e^ Adherence measured as physical activity engagement in unsupervised settings, either as minutes of MVPA, metabolic equivalent values, number/percentage of prescribed sessions completed, or equivalent

^f^ Acknowledgement that the exclusion of the mentioned study types may increase the chances of publication bias

There was no restriction on the health status of participants, setting of interventions, or any co-interventions present. There was also no restriction on publication date or the language of publication. In addition to qualitatively synthesizing information from these studies, studies that included a MICT comparator group were grouped separately for quantitative syntheses via meta-analyses.

### Information sources

MEDLINE (OVID), EMBASE (OVID), PsycINFO (EBSCO), SPORTDiscus (EBSCO), CINAHL (EBSCO) and Web of Science Core Collection were searched from their inception until October 3, 2022. Additional articles not captured in the database searches were identified through citation searching of included articles, as well as other systematic reviews that were captured in the search.

### Search strategy

The main concepts “high-intensity interval training”, “compliance”, and “adherence” were used to conduct the systematic searches in each database. The full search strategy for each database has been published elsewhere [29]; the Medline search strategy can be found as Additional File 2. The search strategy was developed in consultation with a health sciences librarian and peer-reviewed using the 2015 PRESS review guidelines [31]. No limits on date, study type, population, or language of publication were implemented.

### Selection process

De-duplication of retrieved records was done manually by an independent reviewer using EndNote X9 [Clarivate Analytics, 2018]. Manually de-duplicated records were exported into Covidence [Veritas Health Innovation Ltd., 2015], where additional duplicates were programmatically identified and subsequently reviewed before removal [32].

Title and abstract screening were completed in Covidence by two independent reviewers for each record (equally split between 3 individuals). Reviewers met before screening to ensure consistent understanding of eligibility criteria to reduce conflicts. Reviewers were not blinded to study authors or study settings. Conflicts between reviewers were resolved through deliberation. Consensus was achieved in all cases without the need of a third reviewer. Cohen’s kappa score was used to assess interrater reliability, with interpretation of scores following the convention of 0.21–0.40 as fair agreement, 0.41–0.60 as moderate agreement, 0.61–0.80 as substantial agreement, and 0.81–1.00 as almost perfect agreement [33].

Full-text documents for records that met inclusion criteria in title and abstract screening were retrieved and uploaded into Covidence. Full-text screening was performed on each record by two independent reviewers (equally split between 3 individuals). The same process used in title and abstract screening was used in full-text screening: reviewers met to clarify inclusion criteria, then worked independently until all records had been screened; conflict resolution was done through deliberation, and consensus achieved for all records.

Records identified in any language other than English were included in this review, and when needed, two independent reviewers proficient in the language of the record were sought to complete the screening process in the same way as English records. All records not in English that met the inclusion criteria of this review had data extracted by the same reviewers who did the screening for such records.

### Data collection process

Data extraction consisted of a pilot and extraction phase. An initial data extraction form was piloted on five included studies by two independent reviewers. Reviewers met after extraction of the five articles to discuss potential improvements to the data extraction form, and a finalized form approved for all subsequent articles. The finalized data extraction form can be found as Additional File 3. Data from each included study was extracted by independent reviewers, with the majority of studies being doubly extracted, and conflicts resolved by reviewers via discussion at the end of the data extraction phase.

### Risk of Bias

Risk of bias assessments were completed by two independent reviewers for each included study (equally split between 3 individuals). Studies characterized as randomized controlled trials were assessed using the Cochrane Risk of Bias Tool 2.0 (RoB 2.0) [34], while quasi-experimental studies were assessed using the Risk of Bias in Non-Randomized Studies of Interventions tool (ROBINS-I) [35]. After all studies were assessed, resolution of conflicts was conducted via discussion between the two independent reviewers. Risk of bias for each sub-category as well as a general risk of bias score was summarized using the *Robvis* web application [36].

### Synthesis of information

Data extracted from each included study was summarized qualitatively in tabular format and summary statistics are presented. Authors of included studies that did not report means and/or standard deviations (SDs) for the outcome variables of interest were contacted and given a 2-week timeframe to respond with the requested information. In instances where authors were unable to provide the means and/or SDs, the medians, ranges, and interquartile ranges were used to estimate the means and/or SDs using the methods proposed by Weir and colleagues [37]. Specifically, for studies that provided ranges, SD was estimated by using the following formula where *R* is the range [38]:

$$SD\approx \frac{R}{4}$$

For studies that provided interquartile ranges or 95% CIs, SD was estimated by using the Cochrane Handbook estimator calculation where *q*3 is the third quartile and *q*1 is the first quartile [39], and *t* is the distribution value based on degrees of freedom:

$$SD\approx \frac{q3-q1}{1.35}$$

$$SD\approx \frac{\sqrt{n}\left(95\%{CI}_{upper}-95\%{CI}_{lower}\right)}{2(t)}$$

For studies that provided medians along with ranges, 95% CIs, or interquartile ranges, means were estimated by using the calculations proposed by Wan and colleagues where *m* is the median [40]:

$$\overline{x}\approx \frac{q1+m+q3}{3}$$

Studies with insufficient information to estimate means and/or SDs were omitted from the quantitative synthesis (*n* = 6).

To address the first purpose of this study, weighted averages and weighted standard deviations of compliance and adherence to the prescribed exercise type were calculated using the following calculations where *W* is the weighted average, *w* is the study weight, *X* is the study average, *SDw* is the weighted SD, and *M* is the number of non-zero weights [41]:

$$W=\frac{\sum_{i=1}^n{w}_i{X}_i}{\sum_{i=1}^n{w}_i}$$

$${SD}_w=\sqrt{\frac{\sum_{i=1}^n{w}_i{\left({X}_i-W\right)}^2}{\frac{\left(M-1\right)}{M}{\sum}_{i=1}^n{w}_i}}$$

### Meta-analyses

To address the second purpose of this study, two meta-analyses were conducted: one for the compliance outcome variable and one for the adherence outcome variable. All meta-analyses and accompanying figures were generated in *Comprehensive Meta Analysis Version 4* [42]. Inclusion in the meta-analyses required studies to 1) have a MICT comparator group in addition to a HIIT group, 2) report compliance or adherence rates (for compliance: percentage of attendance to supervised exercise sessions; for adherence: percentage of unsupervised exercise sessions completed of the prescribed exercise type), 3) report the means, SDs, and sample sizes for each group, and 4) have a sample size greater than 1. It should be noted that in studies where mean percentage values were 100%, accompanying SD values were 0 (i.e., all participants completed all sessions). These SD values were changed to 0.001%.

Random-effects meta-analyses were conducted to determine the mean differences between HIIT and MICT in each outcome variable. Random-effects analyses consider individual studies’ variance when assigning weights to each study, assesses the between-study variance (via *τ*^*2*^), and allows for the generalizability of results to other comparable studies in the universe that may not have been captured in these syntheses [43]. The generalizability of the results to various populations was made possible as we included studies focusing on populations that present with varying medical conditions as well as studies that focus on insufficiently active but otherwise healthy populations. Considering most included studies in the meta-analyses had relatively small sample sizes, Hedge’s *g* was used as the effect size point estimate for each study, and pooled Hedge’s *g* was used as the mean effect size point estimate in each analysis. All meta-analyses were summarized in forest plots, with key information presented in the results section. A statistically significant negative pooled Hedge’s *g* indicated an outcome variable favoring the MICT condition, while a statistically significant positive pooled Hedge’s *g* indicated an outcome variable favoring the HIIT condition. For all statistical comparisons, alpha was set to .05.

#### Heterogeneity

Various statistical values were interpreted to identify the presence of heterogeneity in each meta-analysis. The prediction interval was used to estimate the 95% dispersion of the mean effect size for each meta-analysis [44]. *Q* statistic was used to determine whether the effect sizes vary among included studies, and *I*^*2*^ statistic was used to determine the proportion of the observed variance that is due to true effects instead of sampling error. A minimum of 10 studies included in each meta-analysis was considered sufficient for heterogeneity values to be deemed reliable [45].

#### Sensitivity Analyses

To assess the robustness of the meta-analyses, one-study removed analysis was performed. One-study removed analysis is a statistical technique that shows what the pooled Hedge’s *g* effect size would be if each included study was removed from the analysis [46]. This technique showed whether any one study had a statistically or clinically significant impact on the pooled effect size compared to the others based on its weighting and individual effect size.

Funnel plots were used as another type of sensitivity analysis to test for publication bias. Historically, smaller studies and studies whose results are regarded as less conclusive (i.e., non-significant) tend to not be published as often as studies with larger sample sizes and stronger treatment effects, creating the potential introduction of publication bias [47]. Visual inspection of funnel plots allowed for the estimation of whether smaller studies have not been published due to publication bias. For each meta-analysis, if there was apparent asymmetry between one side of the funnel plot compared to the other side, imputation of potentially missing studies was performed by using Duval & Tweedie’s trim and fill function [48] to determine what the pooled effect size would be if such missing studies were included in the meta-analyses. In addition to visual inspection of funnel plots, the Begg & Mazumdar’s rank correlation test [49] was used to assess whether there was an inverse correlation between study size and treatment effect. After correcting for ties, 1-tailed tests based on continuity-corrected normal approximations were computed. Significant correlation findings were interpreted as a potential presence of publication bias, and the subsequent use of Duval and Tweedie’s trim and fill function was performed. In situations where no significant correlation was found, visual inspection of funnel plots was still used to assess publication bias as bias cannot be ruled out if the rank correlation test is not significant [49].

#### Moderation Analyses

To address potential wide prediction intervals associated with each mean effect size point estimate, sub-group analyses were conducted to provide more specificity on the effect sizes for given sub-groups within each outcome variable. All sub-groups were created as dichotomous categorical groups and defined a-priori for each outcome. Although all sub-group analyses’ models were computed using random-effects, sub-groups were combined using a fixed-effect model. The sub-groups created for each outcome variable were as follows:

- Compliance: Study design (randomized controlled trial vs. quasi-experimental), medical condition (presence vs. absence), and subjective risk of bias (low/moderate vs. high).
- Adherence: Study design (randomized controlled trial vs. observational study), medical condition (presence vs. absence), subjective risk of bias (low/moderate vs. high), method of measurement (self-report vs. activity tracker), and timepoint of measurement (≤12 weeks vs. > 12 weeks).

For all sub-group analyses, the pooled *τ*^*2*^ value was used to estimate the mean effect size for each sub-group, as using the individual *τ*^*2*^ values on a small number of studies in each sub-group are likely to be imprecise [50]. Comparisons between the calculated mean effect sizes of sub-groups were performed using a *Q* statistic. Like the main meta-analyses, sub-group analyses were summarized using forest plots and an alpha of .05 was used for all statistical comparisons.

### Quality appraisal of the cumulative body of evidence

Quality of the cumulative body of evidence for each outcome variable was appraised using the Grading of Recommendations Assessment, Development and Evaluation (GRADE) approach [51]. Each outcome variable was assessed on factors that could either decrease or increase the quality of the cumulative evidence [52]. Factors that could decrease the quality of evidence included study limitations (risk of bias), inconsistencies of results (heterogeneity), indirectness of evidence, imprecision, and publication bias. The observation of a large magnitude of an effect was used as a factor that could increase the quality of evidence. Each factor was appraised by an independent reviewer in consultation with the GRADE Handbook [52]. The overall quality of the evidence for each outcome variable was determined based on a continuum of four grades: high, moderate, low, and very low. Outcome variables that generally had a higher proportion of randomized controlled trials started on a “high” rating of quality, while outcome variables that generally had a higher proportion of observational trials started on a “low” rating of quality. Fluctuations thereafter were a result of the factor appraisals mentioned above. The results of the quality appraisals were summarized in a GRADE evidence profile table created using the GRADEpro GDT tool [53].

### Equity, diversity, and inclusion statement

Our author group is gender balanced, representative of different disciplines within health sciences, and includes 3 junior, 2 mid-career, and 3 senior researchers living in two different countries, although we acknowledge that both are high-income countries (Canada and Australia). Three of the authors are women (including the senior corresponding author, who also identifies as of Chinese heritage), and two of the authors are part of equity-deserving groups due to their heritage from the Global South and persons of color.

Our systematic review attempted to include studies from all regions of the globe, and efforts were made to include information presented in other languages so that diverse perspectives from traditionally underrepresented, equity-deserving cultures in academia were represented. We also included studies with many diverse forms of conditions so that results could be pertinent to a wide variety of populations regardless of physical ability, mental health status, or medical condition. Similarly, we included studies with samples of varying ages, demographics, regional locations, and biological sex. We sought to gather information on reported gender identities during our data collection phase to be more inclusive of those not conforming to binary gender classifications, although a scarcity of gender identity reporting was noticed in this field of research.

## Results

**Fig. 1:**
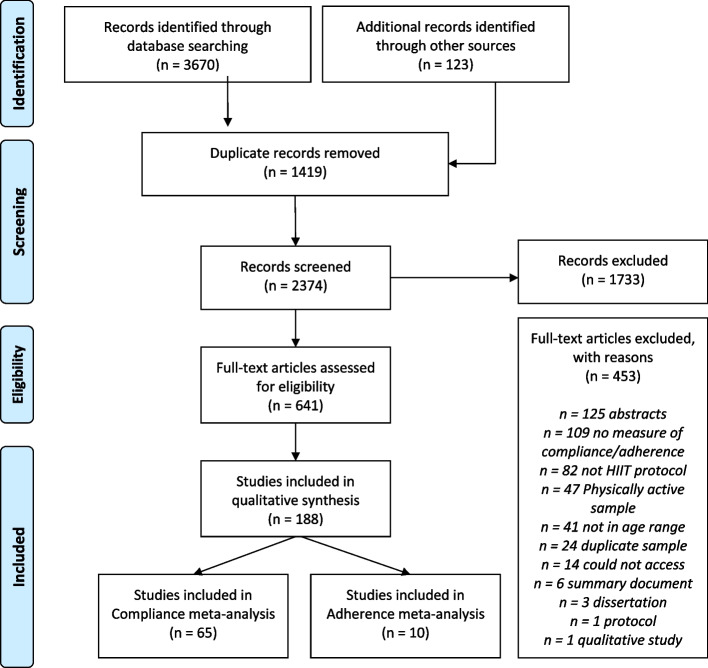
PRISMA Flow Diagram

A PRISMA flow diagram shows the progression of record screening throughout this systematic review (Fig. 1). A total of 3670 records were retrieved via database searches and an additional 123 records were retrieved through manual searching of reference lists for a total of 3793 records. After de-duplication of records, 2374 records went through title and abstract screening. Cohen’s kappa for the title and abstract screening phase was 0.64, indicating moderate agreement between reviewers. Of the 2374 records, 641 went to the full-text screening phase. Cohen’s kappa for the full-text screening phase was 0.77, indicating substantial agreement. After consensus was reached, 188 unique studies were included in this systematic review with a total sample size of 8928 participants [25, 26, 54–239].

### Study characteristics

General information about each study can be found in tabular form as Additional File 4. The majority of articles were published between 2016 and 2022 (*n* = 131; 69.7%; range 1996 to 2022), with 2016 having the most articles published of any given year (*n* = 26; 13.8%). Only one included article [76] was in a language other than English (Spanish; 0.53%). Most studies were conducted at a single centre (*n* = 176; 93.6%) with only 12 studies reporting multi-center designs (6.4%). Research studies were conducted in 30 different countries, the most prominent being Canada (*n* = 31; 16.5%), the United States (*n* = 24; 12.8%), and Norway (*n* = 22; 11.7%). 74.5% of included studies reported receiving funding (*n* = 140); 6.9% declared at least one conflict of interest (*n* = 13), 79.3% had no conflicts to declare (*n* = 149), and 13.8% had no declaration statement (*n* = 26). Ethical approval from an institutional board and participant consents were obtained for 98.9% of included studies (*n* = 186).

#### Study Design

Information about studies’ design and population of interest are found in Additional File 5. Most included studies were prospective, with only 4 studies reporting on retrospective data (2.1%). One-hundred and 55 studies were designed as randomized controlled trials or variations thereof (82.4%), while 31 studies were quasi-experimental (16.5%), one study was an exploratory retrospective analysis (0.5%), and one study was a case study (0.5%). Of the 188 included studies, 49 included insufficiently active but otherwise healthy individuals (26.1%). The remaining 139 studies included individuals who presented with at least one medical condition (73.9%). A total of 46 different medical conditions were captured in this systematic review, with the most prominent conditions being cancer (*n* = 21; 11.2%), obesity (*n* = 19; 10.1%), coronary artery disease (*n* = 13; 6.9%), and type 2 diabetes (*n* = 13; 6.9%).

#### Group Characteristics

Additional File 6 details information regarding sample size and group characteristics in each study. Total sample size was on average 48 participants per study (*SD*: 46.2) and ranged from 1 participant [106] to 255 participants [73]. All included studies had a HIIT intervention group; 87 of them included a MICT group (46.3%), 96 included a control group (51.1%), and 47 included another type of group (25%). Mean age for individuals allocated to a HIIT group was 46.6 years (*SD*: 13.4). For studies that included a MICT group, mean age for individuals allocated to MICT was 47.3 years (*SD*: 13.8). Regarding biological sex, 53% (*SD*: 34%) and 56% (*SD*: 36.1%) of HIIT and MICT participants were male, respectively. No study reported on participants’ gender or sexual identity.

#### Intervention Characteristics

Additional File 7 summarizes characteristics of the supervised HIIT protocols and where applicable, MICT interventions introduced in each study, while Table 2 provides details on unsupervised, prescribed exercise interventions. Most interventions were one-on-one supervised exercise sessions (*n* = 159; 84.6%), with the remainder being group sessions ranging between 2 [56, 99] to 15 [209] participants in each session. For studies with a supervised intervention, participants engaged in 1 to 8 sessions per week, with the modal frequency being 3 sessions per week (*n* = 105; 55.9%) and total number of sessions averaging 30 (*SD*: 18.2) and ranging between 6 sessions [151, 201] and 104 sessions [121]. For studies with an unsupervised exercise intervention, participants were most often prescribed 3 sessions per week (*n* = 13; 43.3%) of their specific exercise type (HIIT or MICT), ranging between 1 to 6 sessions per week. Five studies prescribed exercise in terms of minutes of MVPA per week ranging between 75 and 180 minutes of any physical activity meeting a minimum moderate-intensity threshold. In unsupervised settings, exercise was prescribed for variable lengths of time ranging between 4 weeks [139] to 80 weeks [129], with 52 weeks being the most commonly prescribed length (*n* = 7; 23.3%).

**Table 2:** Characteristics of Studies with Unsupervised Exercise Components

Study Reference	Number of Prescribed Unsupervised Sessions	Length of Unsupervised Time Period	FITT Description	
			HIIT	MICT
Aamot et al. [54]	150 minutes MVPA/week	52 weeks post-intervention	–	–
Bjorke et al. [73]	2 sessions/week	24 weeks	5-10 × 2 min; 80–90% HRR; Running/Walking/Cycling; 60s recovery.	75 min; 40–50% HRR; Running/Walking/Cycling.
Currie et al. [85]	1 session/week	12 weeks post-intervention	Lower limb exercises	Lower limb exercises
Dowd et al. [94]	150 minutes MVPA/week	12 weeks post-intervention	–	–
Emtner et al. [99]	2 sessions/week	8 weeks post-intervention	Pool swimming	–
Gauthier et al. [108]	3 sessions/week	6 weeks	20x30s; 6–8 BORG10 RPE; Wheelchair propulsion; 60s recovery.	30 min; 4–5 BORG10 RPE; Wheelchair propulsion.
Guillamo et al. [118]	4–6 sessions/week	20 weeks post-intervention	3 × 3-5 min; 17–18 BORG20 RPE; Biking; 3 min recovery	–
Heje et al. [123]	3 sessions/week	8 weeks post-intervention	2 sets of 5x10s; Maximal sprints; Biking; 50s recovery.	–
Hesketh et al. [124]	3 sessions/week	12 weeks	4-9x 60s; 80% HR max; Body weight exercises; 60s recovery.	45 min; 50–70% HR max; Home-based exercises.
Howden et al. [129]	3–4 sessions/week	80 weeks	4x4min; 95% HR peak; Running/Biking/Elliptical; 3 min recovery.	–
Ivanova et al. [134]	1–2 sessions/week	24 weeks post-intervention	4-10x 60s; 90% HR peak; Treadmill/Stationary bike/Elliptical; 60s recovery.	20-50 min; 65% HR peak; Treadmill/Stationary bike/Elliptical.
Jung et al. [139]	1–2 sessions/week	4 weeks post-intervention	10x 60s; 90% HR peak; Treadmill/Stationary bike/Elliptical; 60s recovery.	50 min; 65% HR peak; Treadmill/Stationary bike/Elliptical.
Jung et al. [26]	3 sessions/week	52 weeks post-intervention	10x 60s; 90% HR peak; Treadmill/Stationary bike/Elliptical; 60s recovery.	50 min; 65% HR peak; Treadmill/Stationary bike/Elliptical.
Karstoft et al. [142]	5 sessions/week	16 weeks	10x3min; 70% VO2 peak; Walking; 3 min recovery.	60 min; 55% VO2 peak; Walking.
Keogh et al. [147]	4 sessions/week	8 weeks	5x45s; High intensity; Biking; 90s recovery.	20 min; Moderate intensity; Biking.
Locke et al. [157]	75–150 minutes MVPA/week	24 weeks post-intervention	10x60s; 85% HR peak; Walking/Biking/Elliptical; 60s recovery.	30 min; 65% HR peak; Walking/Biking/Elliptical.
Madssen et al. [163]	3 sessions/week	52 weeks post-intervention	4x4min; 85–95% HR max; Running/Biking/Skiing; 3 min recovery.	–
Mendelson et al. [167]	3 sessions/week	16 weeks post-intervention	22x60s; 100% PPO; Cycle ergometer; 60s recovery.	32-44 min; 50% PPO; Cycle ergometer.
Midtgaard et al. [171]	180 minutes MVPA/week	52 weeks post-intervention	–	–
Moholdt et al. [173]	3–4 sessions/week	24 weeks post-intervention	4x4min; 90% HR max; Biking; 3 min recovery.	46 min; 70% HR max; Biking.
Moholdt et al. [174]	3 sessions/week	24 weeks	4x4min; 85–95% HR max; Running/Biking/Swimming; 3 min recovery.	–
Pattyn et al. [184]	150 minutes MVPA/week	52 weeks	–	–
Poon et al. [188]	3 sessions/week	8 weeks	10x60s; 80–90% HR max; Running; 60s recovery.	50 min; 65–70% HR max; Running.
Poon et al. [189]	3 sessions/week	16 weeks post-intervention	6-12x 60s; 80–90% HR max; Running; 60s recovery.	40 min; 65–70% HR max; Walking.
Roy et al. [198]	3 sessions/week	52 weeks	HIIT exercises; 80–90% HR max; Walking/Biking	–
Scott et al. [207]	3 sessions/week	6 weeks	6-10x 60s; 80% HR max; Body weight exercises; 60s recovery.	–
Smith-Ryan et al. [213]	2 sessions/week	12 weeks	10x60s; 75–95% HR max; Home-based exercises; 60s recovery.	–
Taylor et al. [217]	3 sessions/week	52 weeks post-intervention	4x4min; RPE 15–18 BORG20 RPE; Home-based exercises; 3 min recovery.	40 min; RPE 11–13 BORG20 RPE; Home-based exercises.
Valent et al. [226]	2–3 sessions/week	8–12 weeks	6-8 × 2-3 min; 60–80% HRR; Hand cycling; 1-2 min recovery.	–
Vella et al. [227]	3 sessions/week	5 weeks post-intervention	10x60s; 75–80% HRR; Treadmill/Cycle ergometer/Elliptical; 60s recovery.	20 min; 55–59% HRR; Treadmill/Cycle ergometer/Elliptical.

FITT descriptions follow the convention of duration of intervals/exercise, intensity of exercise sessions, modality/type of exercise, and for HIIT interventions, recovery duration. *MVPA* moderate-to-vigorous physical activity, *HR* heart rate, *HRR* heart rate reserve, *VO2* volume of oxygen consumption, *PPO* peak power output, *BORG10 RPE* rating of perceived exertion based on the 0–10 scale, *BORG20 RPE* rating of perceived exertion based on the 6–20 Borg scale

For both supervised and unsupervised interventions, there was wide diversity in exercise prescription; each HIIT and MICT intervention has been summarized in Additional File 7 and Table 2 according to their intensity, time, and type. In addition to HIIT or MICT, 14.4% of interventions also had a strength training component (*n* = 27), 5.9% had other exercise components such as stretching, yoga, cross-training, etc. (*n* = 11), and 13.8% had some form of educational counselling/behaviour change technique component (*n* = 26).

### Risk of Bias assessment

**Fig. 2:**
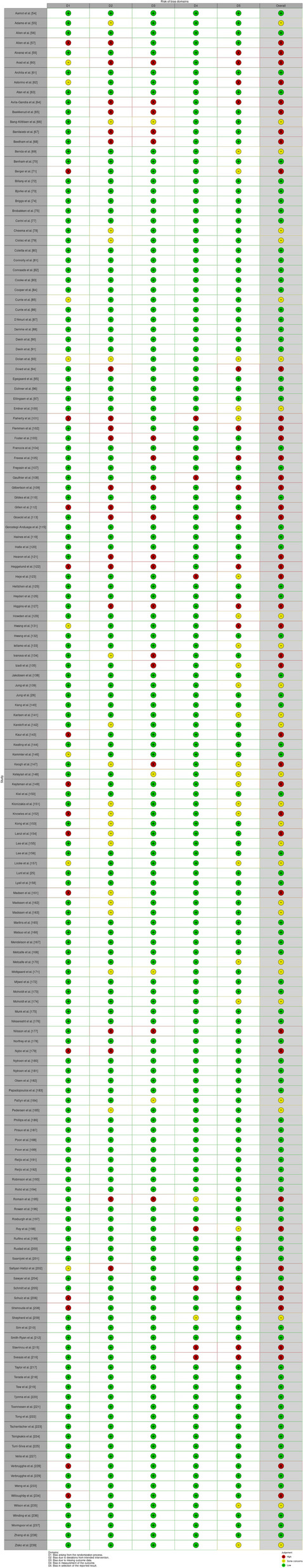
Risk of Bias 2.0 Traffic Light Plot (*n* = 156)

**Fig. 3:**
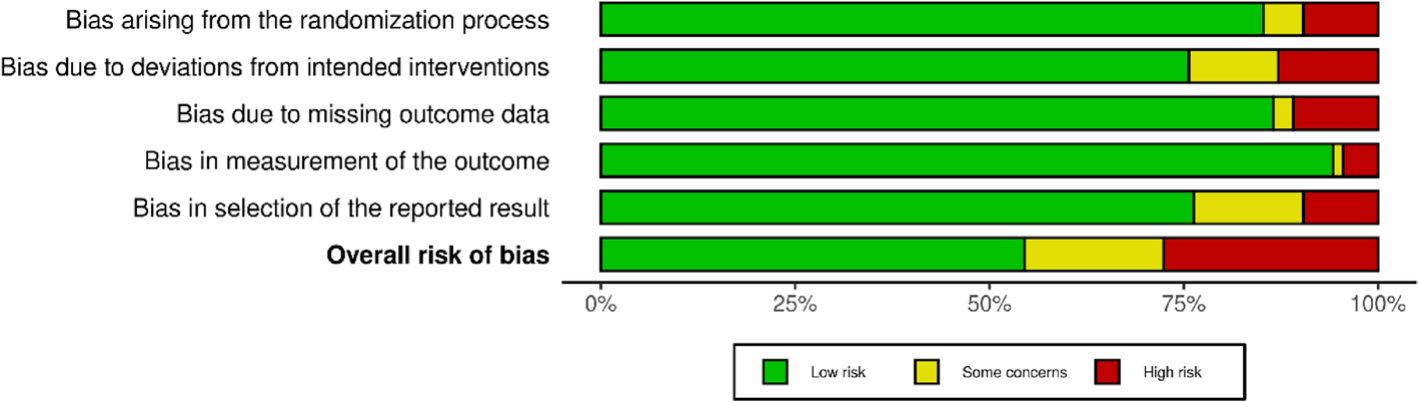
Summary Plot of Risk of Bias 2.0 Assessments (*n* = 156)

Risk of bias assessments for each randomized controlled trial can be found in Fig. 2, with accompanying summary results illustrated in Fig. 3. A total of 156 studies were assessed, with 85 (54.5%) showing overall low risk of bias, 28 (17.9%) showing some concerns, and 43 (27.6%) showing high risk of bias based on the 5 domains of RoB 2.0 [34].

**Fig. 4:**
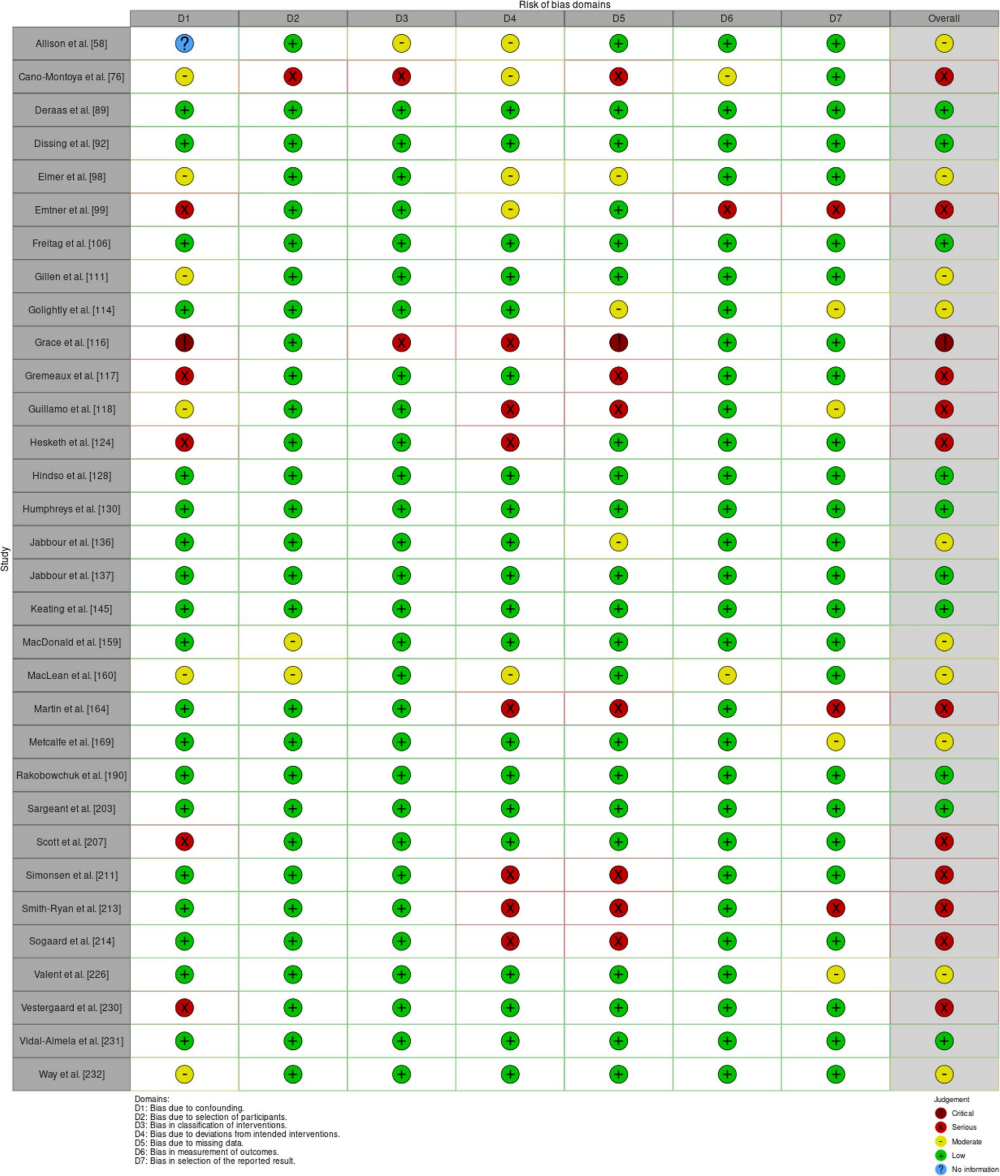
ROBINS-I Traffic Light Plot (*n* = 32)

**Fig. 5:**
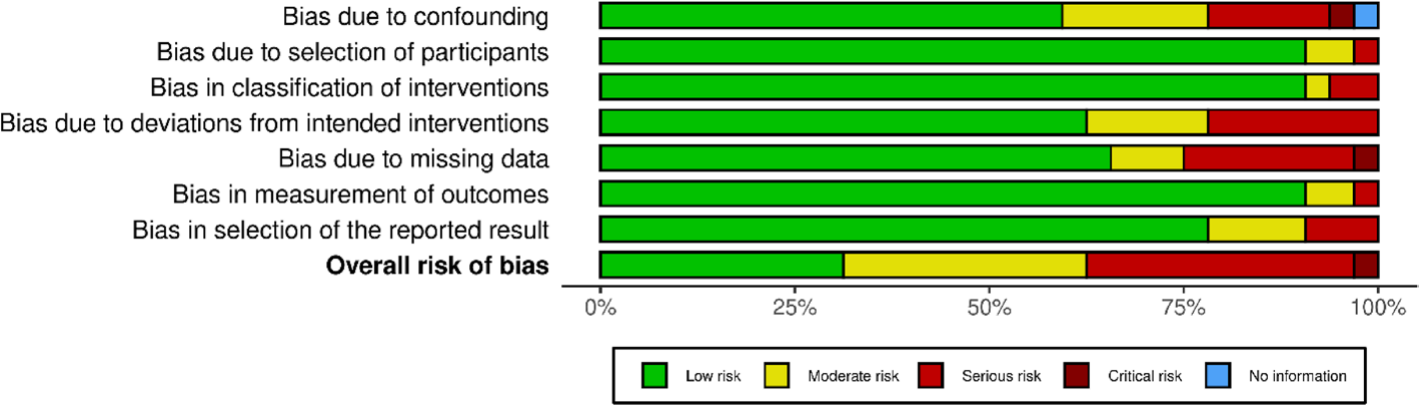
Summary Plot of ROBINS-I Assessments (*n* = 32)

Individual study results and summary statistics of risk of bias of studies that were not randomized controlled trials are depicted in Figs. 4 and 5, respectively. Thirty-two studies were assessed using the 7 domains of ROBINS-I [35]: 10 studies (31.3%) were categorized as low risk of bias, 10 (31.3%) were moderate risk, 11 (34.4%) were categorized as high risk of bias, and 1 study (3.1%) was categorized as critical risk of bias.

### Compliance

#### Weighted Average and SD

Of the 188 studies included in the systematic review, 172 reported compliance rates to a supervised HIIT intervention, and 76 reported compliance rates to supervised MICT. Individual study results and dropout rates can be found in Additional File 8. On average, 12.9% (*SD:* 13%) and 11.8% (*SD:* 11.6%) of participants dropped out from a supervised HIIT or MICT intervention, respectively. Six studies reported compliance rates in units other than percentage of supervised sessions completed and were therefore omitted from quantitative syntheses. The results of the remaining 166 studies and 70 studies were used to calculate the weighted average and weighted SD for compliance rates to supervised HIIT and MICT interventions, respectively. Overall, compliance to supervised HIIT interventions averaged 89.4% (*SD*: 11.8%), while compliance to supervised MICT interventions averaged 92.5% (*SD*: 10.6%).

#### Meta-Analysis

**Fig. 6:**
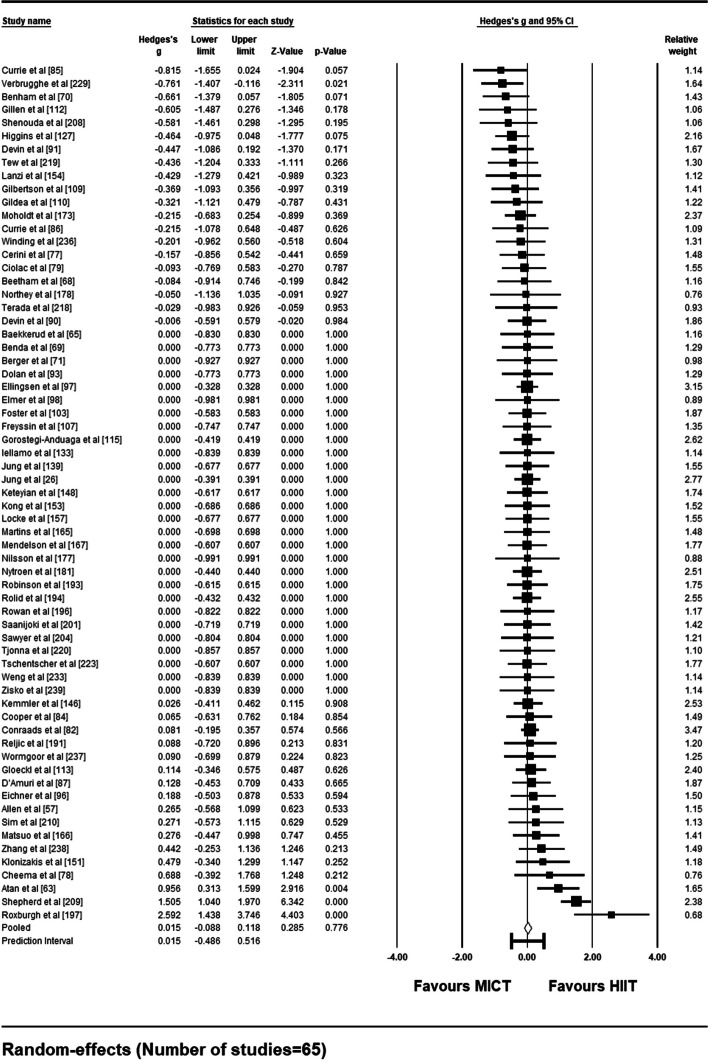
Forest Plot Comparing Compliance Rates to HIIT vs. MICT Interventions

**Fig. 7:**
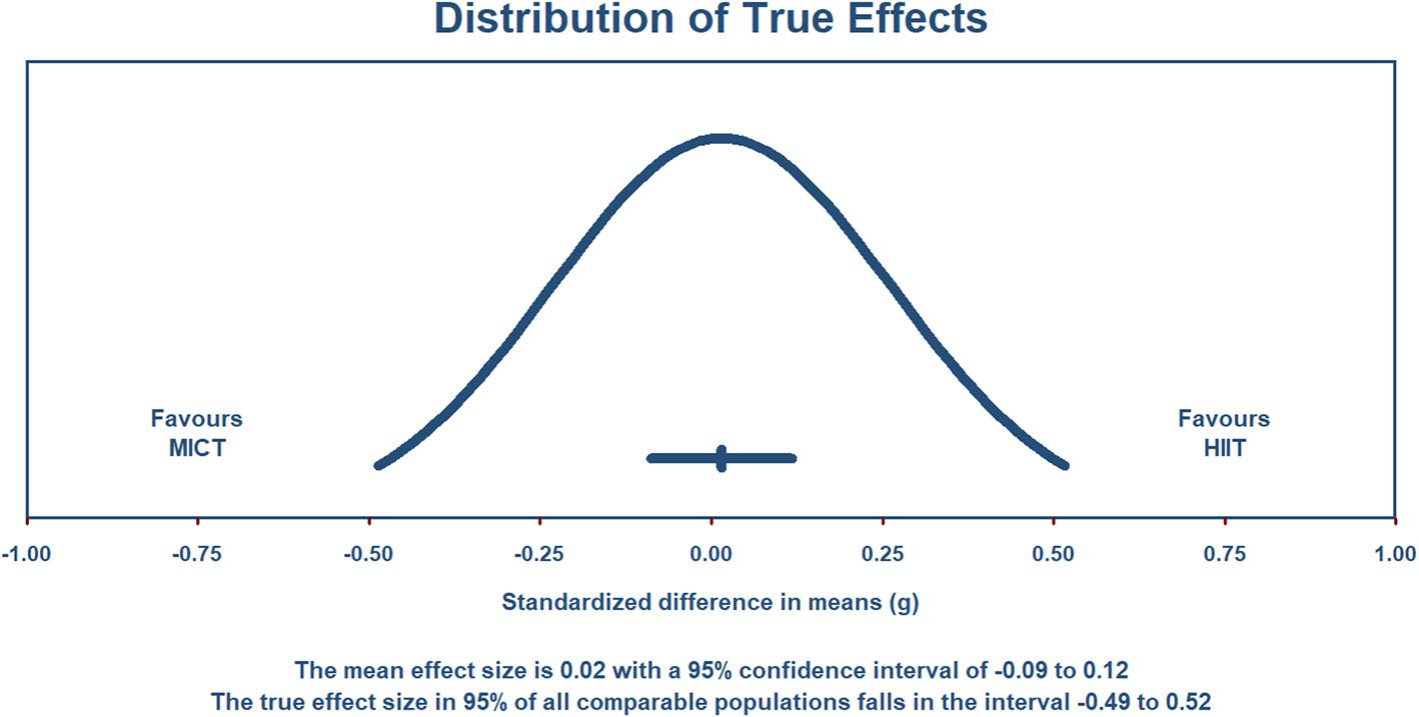
Pooled Effect Size Point Estimate, 95% Confidence Interval, and Accompanying Prediction Interval for Compliance Rates to HIIT vs. MICT Interventions

Sixty-five studies met the criteria necessary to be included in a random-effects meta-analysis comparing compliance rates between supervised HIIT and MICT interventions. A forest plot depicting each study’s weight, effect size and accompanying 95% confidence interval can be found as Fig. 6. Pooled results show no significant difference in compliance rates between supervised HIIT and MICT interventions [Hedge’s *g* = 0.015 (95% CI: − 0.088 – 0.118), *p* = .78]. The prediction interval demonstrates that 95% of true effects for all comparable studies in the universe fall somewhere between − 0.49 and 0.52 from the reference line (see Fig. 7). Between-study variance of the true effects is denoted by *τ*^2^, with standard deviation being the square root (*τ*^2^ = 0.060). Effect size point estimates significantly varied among included studies [*Q* (64) = 100.88, *p* = .002]. *I*^*2*^ statistic revealed that 36.56% of the observed variance was due to true effects, with the remaining proportion attributable to sampling error.

#### Sensitivity Analysis

One-study removed analyses showed that no study included in this meta-analysis had a significant statistical impact on the pooled effect size compared to all other studies (*p* > .05), suggesting the analysis is robust.

#### Publication Bias

**Fig. 8:**
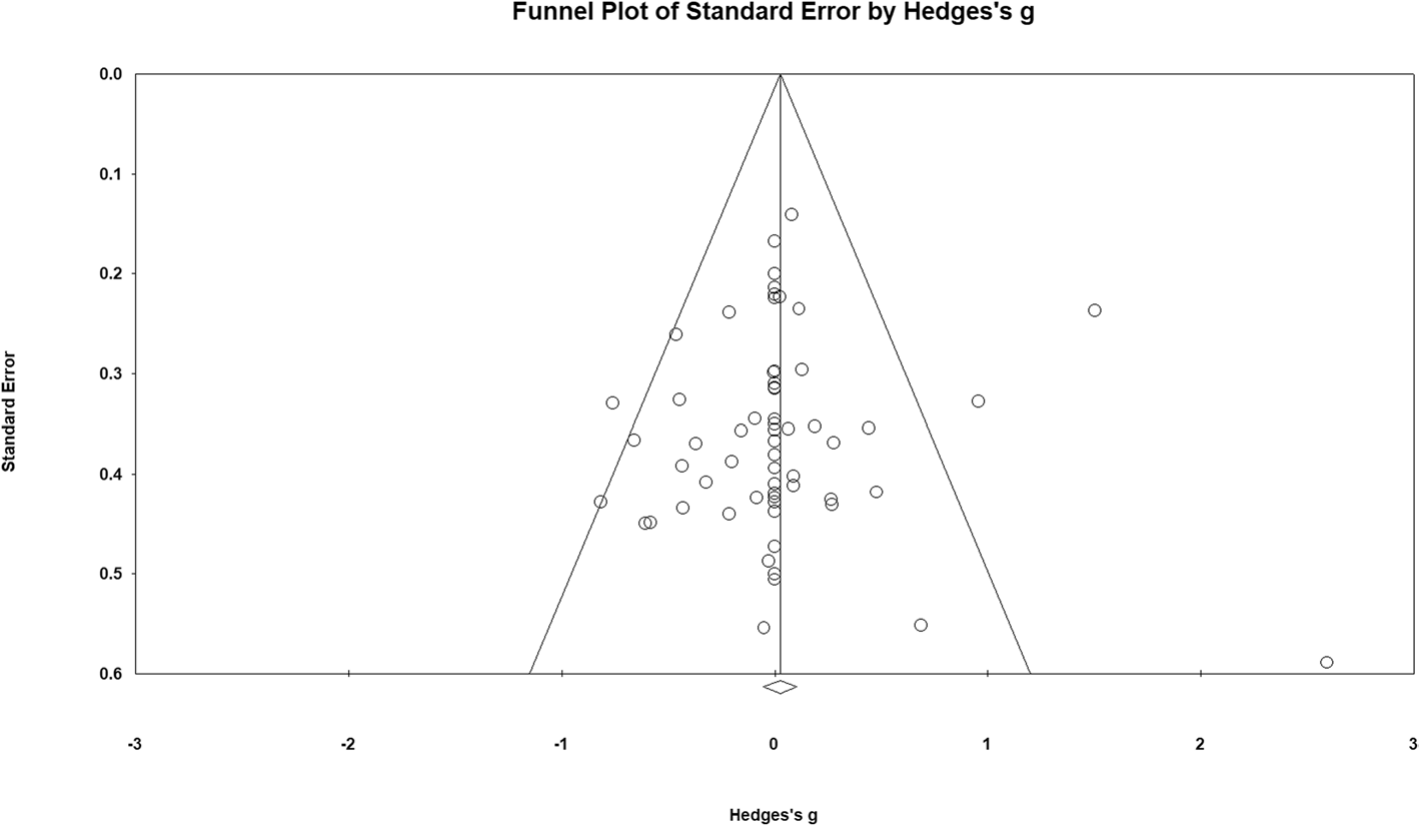
Funnel Plot for Compliance Rates to HIIT vs. MICT Interventions

Begg & Mazumdar’s rank correlation test [49] was used to assess the presence of publication bias. Kendall’s *τ*_b_ for 1-tailed test with continuity correction suggested low publication bias in the compliance meta-analysis (*τ*_b_ = 0.131, *p* = .062). Visual inspection of a funnel plot mapping each included study relative to the pooled effect size revealed symmetry on both sides of the reference line, also suggesting low publication bias (see Fig. 8). As a result, no potential missing studies were imputed in the meta-analysis through the trim and fill function.

#### Moderation Analyses

Of the three planned sub-group analyses for the compliance outcome variable, two were conducted. Only one study included in the meta-analysis was not a randomized controlled trial [98]. Therefore, sub-group analysis based on study design (randomized control trial vs. quasi-experimental study) was waived.

**Fig. 9:**
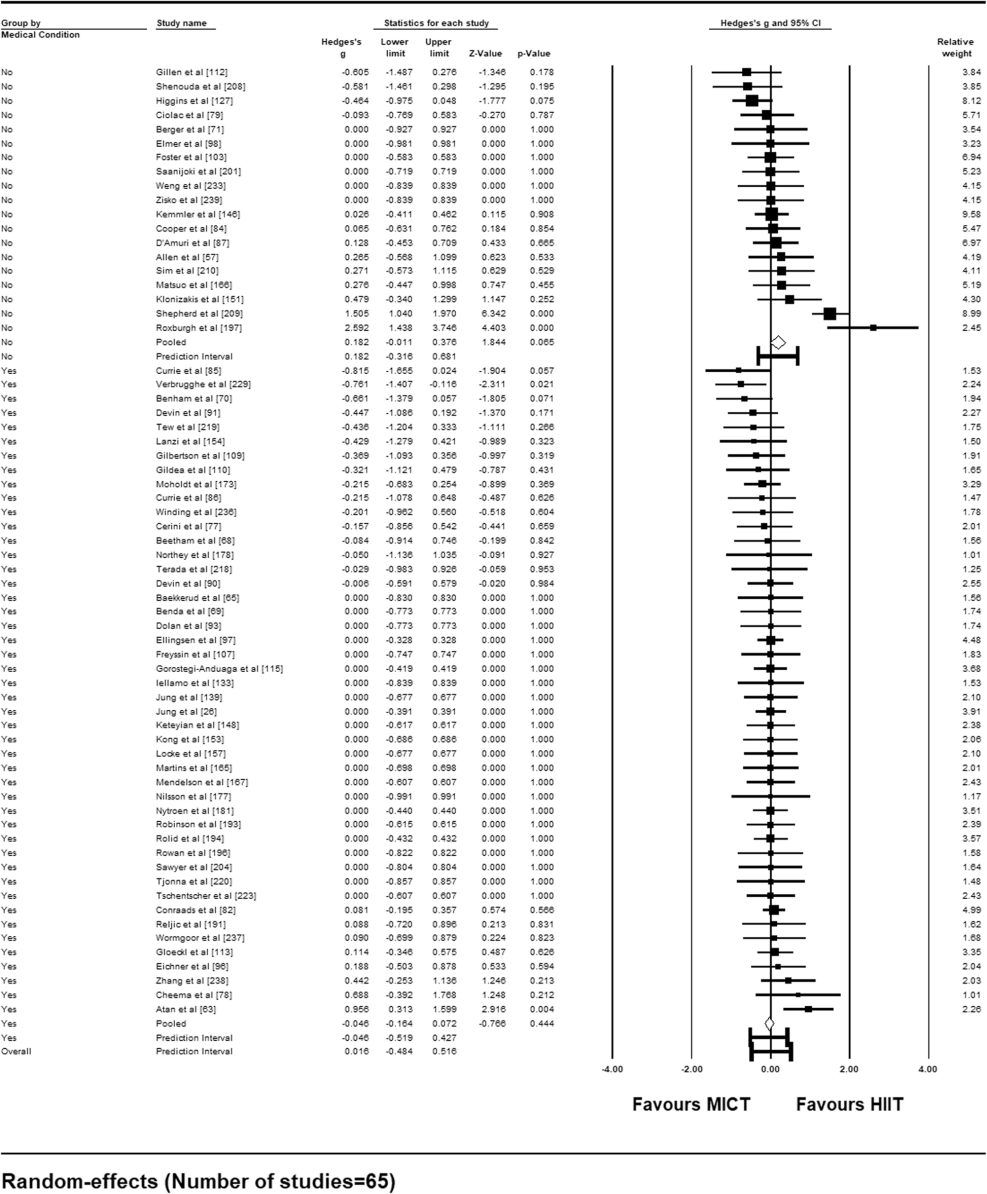
Forest Plot of Sub-Group Analysis (presence vs. absence of medical condition) for Compliance Rates to HIIT vs. MICT Interventions

Sub-group analysis based on the presence vs. absence of a medical condition is summarized in a forest plot (Fig. 9). Of the 65 studies included in the meta-analysis, 46 had a presence of a medical condition (70.8%). For those presenting with a medical condition, mean effect size was not significant [Hedge’s *g* = − 0.046 (95% CI: − 0.164 – 0.072), *p* = .44] with a prediction interval of − 0.519 to 0.427. For the remaining 19 studies on insufficiently active but otherwise healthy samples, mean effect size was also not significant [Hedge’s *g* = 0.182 (95% CI: − 0.011 – 0.381), *p* = .065] with a prediction interval of − 0.316 to 0.681. Comparisons between the two mean effect sizes using a *Q*-statistic showed a significant difference in compliance rate between those with a presence vs. absence of a medical condition [*Q* (1) = 3.90, *p* = .048].

**Fig. 10:**
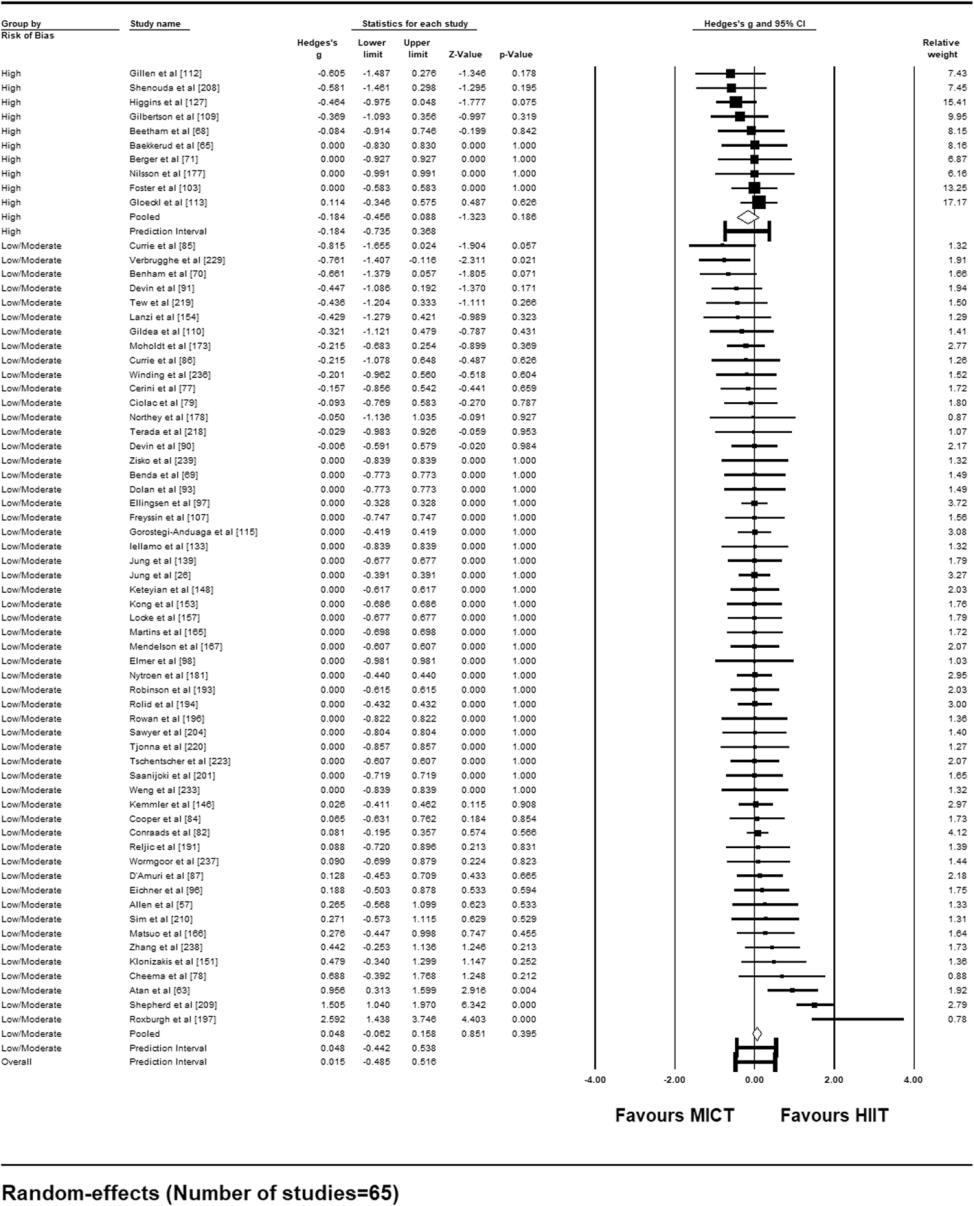
Forest Plot of Sub-Group Analysis (low/moderate vs. high risk of bias) for Compliance Rates to HIIT vs. MICT Interventions

Sub-group analysis based on subjective risk of bias (low/moderate vs. high) is summarized as a forest plot (Fig. 10). Ten studies were assessed to have high risk of bias, with the other 55 studies having low/moderate risk (84.6%). Mean effect sizes for both sub-groups were not statistically significant (*ps* > .05), and comparison between the two mean effect sizes using a *Q*-statistic showed no significant difference in compliance rates between studies with a high risk of bias compared to studies with a low/moderate risk of bias [*Q* (1) = 2.39, *p* = .122].

### Adherence

#### Weighted Average and SD

Thirty studies reported adherence rates to unsupervised, real-world HIIT interventions, while 17 studies reported adherence rates to MICT. A summary of these results can be found in Table 3. There was greater variety in the method of measurement, unit of measurement (e.g., MVPA/week as opposed to adherence to prescribed exercise type), and timepoint of measurement of adherence rates when compared to compliance rates. As a result, 15 of the 30 studies were included in the calculation of weighted average and weighted SD for HIIT, as the other 15 reported adherence rates in units other than percentage of exercise sessions completed in the prescribed exercise type. Ten studies were used in the weighted average and SD calculation for MICT. On average, adherence rate to unsupervised, real-world HIIT sessions was 63% (*SD*: 21.1%). Adherence to MICT sessions was 68.2% (*SD*: 16.2%).

**Table 3:** Adherence to Unsupervised Interventions

Study Reference	Method of Measurement	Unit of Measurement	Timepoint(s) of Measurement	Adherence Result (SD)	
				HIIT	MICT
Aamot et al. [54]	Self-Report	Percentage of reported regular exercisers	52 weeks post-intervention	72%	–
Bjorke et al. [73]	Activity Tracker	Percentage of prescribed exercise sessions completed	24 weeks	52% (32%)	65% (32%)
Currie et al. [85]	Activity Tracker	Percentage of prescribed exercise sessions completed	12 weeks post-intervention	91.7% (83.3%)	100%
Dowd et al. [94]	Self-Report	Number of MVPA minutes per week	12 weeks post-intervention	131.5 (32.1)	–
Emtner et al. [99]	Self-Report	Number of exercise sessions completed per week	8 weeks post-intervention	2	–
Gauthier et al. [108]	Self-Report	Percentage of prescribed exercise sessions completed	6 weeks	86.1% (11.7%)	97.8% (9.4%)
Guillamo et al. [118]	Self-Report	Percentage of prescribed exercise sessions completed	20 weeks post-intervention	30%	–
Heje et al. [123]	–	Number of exercise minutes per week	8 weeks post-intervention	30–50	
Hesketh et al. [124]	Activity Tracker	Percentage of prescribed exercise sessions completed	12 weeks	39% (36%)	48% (35%)
Howden et al. [129]	Activity Tracker	Percentage of prescribed exercise sessions completed	80 weeks	88% (11%)	–
Ivanova et al. [134]	Activity Tracker	Number of MVPA minutes per week	4- and 24-weeks post-intervention	4: 313.5 (88.3) 24: 290.8 (122.7)	4: 313.5 (88.3) 24: 290.8 (122.7)
Jung et al. [139]	Self-Report	Percentage of prescribed exercise sessions completed	4 weeks post-intervention	89% (11%)	71% (31%)
Jung et al. [26]	Activity Tracker	Change in MVPA minutes per week	12-, 24-, and 52-weeks post-intervention	12: 68.5 24: 24.4 52: 2.2	12: 86.4 24: 99 52: 61.6
Karstoft et al. [142]	Activity Tracker	Percentage of prescribed exercise sessions completed	16 weeks	85% (4%)	94% (6%)
Keogh et al. [147]	Self-Report	Percentage of prescribed exercise sessions completed	8 weeks	94% (8%)	88% (12%)
Locke et al. [157]	Activity Tracker	Number of MVPA10+ minutes per week	24 weeks post-intervention	69.4 (11.7)	53 (16.9)
Madssen et al. [163]	Self-Report	Number of exercise sessions completed per week	52 weeks post-intervention	2–3	–
Mendelson et al. [167]	Activity Tracker	Number of MVPA minutes per week	16 weeks post-intervention	105 (90)	82 (53)
Midtgaard et al. [171]	Self-Report	Percentage of reported regular exercisers	52 weeks post-intervention	70.4%	–
Moholdt et al. [173]	Self-Report	Percentage of reported regular exercisers	24 weeks post-intervention	73.9%	68%
Moholdt et al. [174]	Self-Report	Number of exercise sessions completed per week	24 weeks	1.6 (1.6)	–
Pattyn et al. [184]	Activity Tracker	Percentage of reported regular exercisers	52 weeks	93.1%	89.6%
Poon et al. [188]	Activity Tracker	Percentage of prescribed exercise sessions completed	8 weeks	90.1% (4.3%)	95.8% (3.3%)
Poon et al. [189]	Activity Tracker	Percentage of prescribed exercise sessions completed	16 weeks	84% (8.4%)	83.8% (4.3%)
Roy et al. [198]	Activity Tracker	Percentage of reported regular exercisers	52 weeks	23.1%	–
Scott et al. [207]	Activity Tracker	Percentage of prescribed exercise sessions completed	6 weeks	95% (2%)	–
Smith-Ryan et al. [213]	Activity Tracker	Percentage of prescribed exercise sessions completed	12 weeks	63.3% (36.9%)	–
Taylor et al. [217]	Self-Report	Number of exercise sessions completed per week	12-, 24-, and 52-weeks post-intervention	12: 2.8 (1.7) 24: 3.5 (1.5) 52: 3.1 (1.8)	12: 3.3 (1.8) 24: 3.7 (1.6) 52: 3.5 (2.1)
Valent et al. [226]	Self-Report	Percentage of prescribed exercise sessions completed	12 weeks	79.2% (12.5%)	–
Vella et al. [227]	Activity Tracker	Percentage of prescribed exercise sessions completed	8 weeks post-intervention	93.4% (8.3%)	93.1% (10.6%)

Activity tracker measurement includes heart rate monitors, wearable technology, and accelerometry data. *MVPA* moderate-to-vigorous physical activity, *MVPA10+* moderate-to-vigorous physical activity in bouts of 10 minutes or more

#### Meta-Analysis

**Fig. 11:**
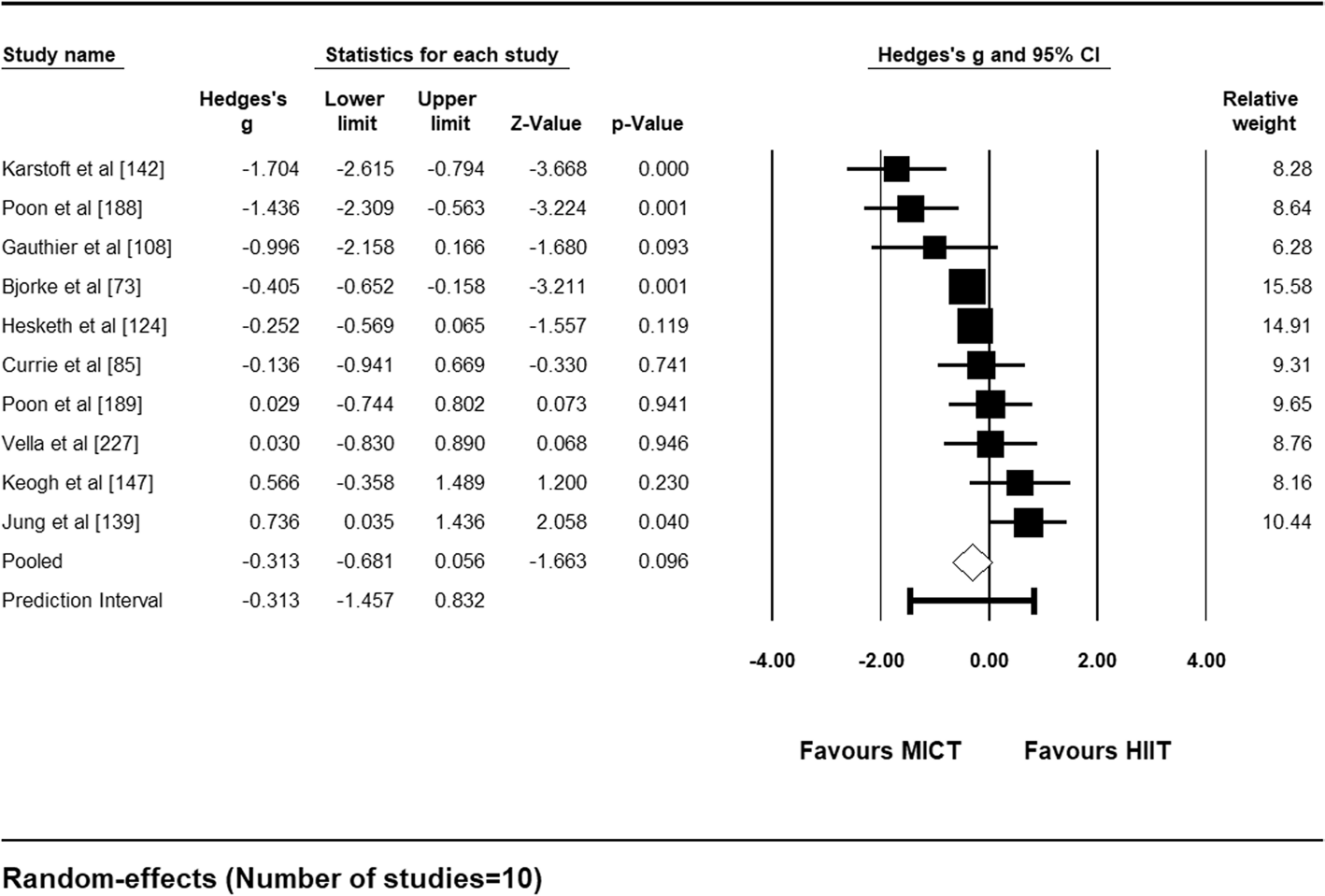
Forest Plot Comparing Adherence Rates to HIIT vs. MICT Interventions

**Fig. 12:**
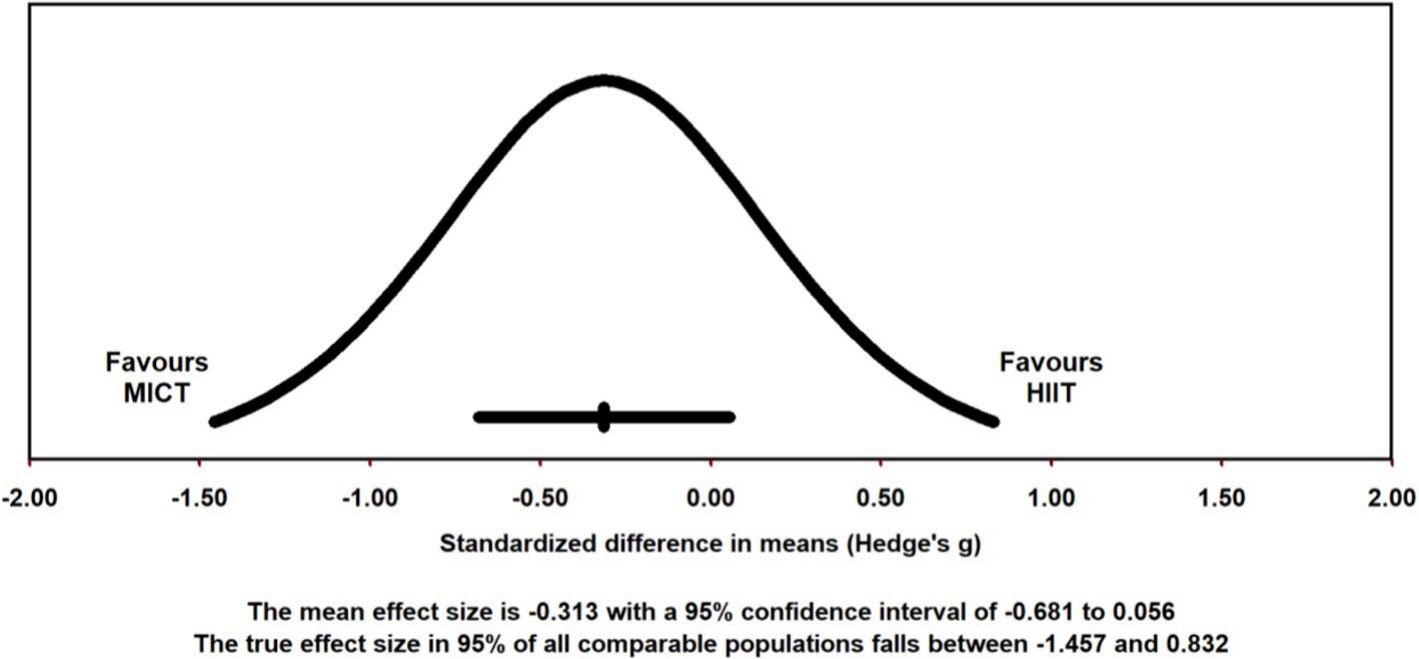
Pooled Effect Size Point Estimate, 95% Confidence Interval, and Accompanying Prediction Interval for Adherence Rates to HIIT vs. MICT Interventions

Ten studies met the criteria necessary to be included in a random-effects meta-analysis comparing adherence rates between unsupervised, real-world HIIT and MICT interventions. A forest plot depicting each study’s weight, effect size and accompanying 95% confidence interval can be found as Fig. 11. Pooled results showed no significant difference in adherence rates between unsupervised HIIT and MICT interventions [Hedge’s *g* = − 0.313 (95% CI: − 0.681 – 0.056), *p* = .096]. The prediction interval demonstrates that 95% of true effects for all comparable studies in the universe fall somewhere between − 1.457 and 0.832 from the reference line (see Fig. 12). Between-study variance of the true effects (*τ*^2^) was calculated to be 0.211. Effect size point estimates significantly varied among included studies [*Q* (9) = 30.96, *p* < .001]. Based on *I*^*2*^ statistic, 70.93% of the observed variance was due to true effects, with the remaining proportion attributable to sampling error.

*Sensitivity Analysis*. One-study removed analyses identified two studies that significantly influenced the pooled effect size. The removal of Jung and colleagues [139] from the main analysis resulted in a statistically significant pooled effect size favoring the MICT interventions (Hedge’s *g* = − 0.426, *p* = .016). Similarly, removing Keogh and colleagues [147] from the main analysis resulted in a statistically significant pooled effect size favoring the MICT interventions (Hedge’s *g* = − 0.388, *p* = .043).

**Fig. 13:**
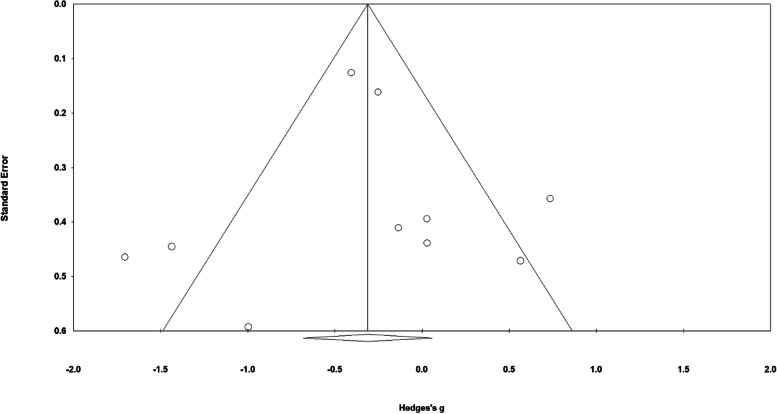
Funnel Plot for Adherence Rates to HIIT vs. MICT Interventions

*Publication Bias*. The rank correlation test was not used as a measure of publication bias since only 10 studies were included in this meta-analysis and concerns over low statistical power have been previously raised [240]. Funnel plot inspection was used instead. Visual inspection of the plot may have suggested publication bias as an unequal number of studies were found at bottom of the plot (see Fig. 13), but the trim and fill function had 0 adjusted values to the left or right of the mean, indicating a non-significant change in mean effect size due to publication bias.

#### Moderation Analyses

Three of the five planned sub-group analyses were completed for the adherence meta-analysis. Sub-group analysis based on study design was waived as only one included study was not a randomized controlled trial [124]. Similarly, all 10 included studies were on populations presenting with a medical condition, so sub-group analysis based on the presence vs. absence of a medical condition was also waived. For the remaining sub-group analyses, results should be interpreted with caution due to the low number of studies aggregated in each sub-group.

**Fig. 14:**
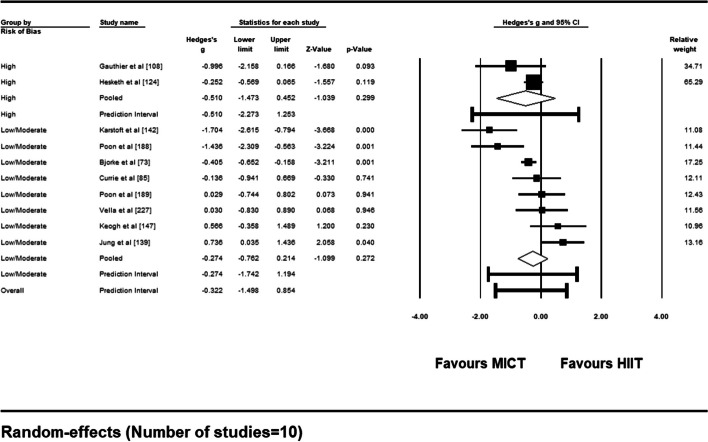
Forest Plot of Sub-Group Analysis (low/moderate vs. high risk of bias) for Adherence Rates to HIIT vs. MICT Interventions

For sub-group analysis based on risk of bias assessment, 2 studies had a subjective rating of high risk and the remaining 8 were rated as low/moderate risk. Summary results can be found as a forest plot (Fig. 14). Mean effect sizes were not statistically significant for either sub-group (*ps* > .05) and comparison between the two mean effect sizes was also non-significant, suggesting that adherence rates were not different dependent on risk of bias rating [*Q* (1) = 0.185, *p* = .668].

**Fig. 15:**
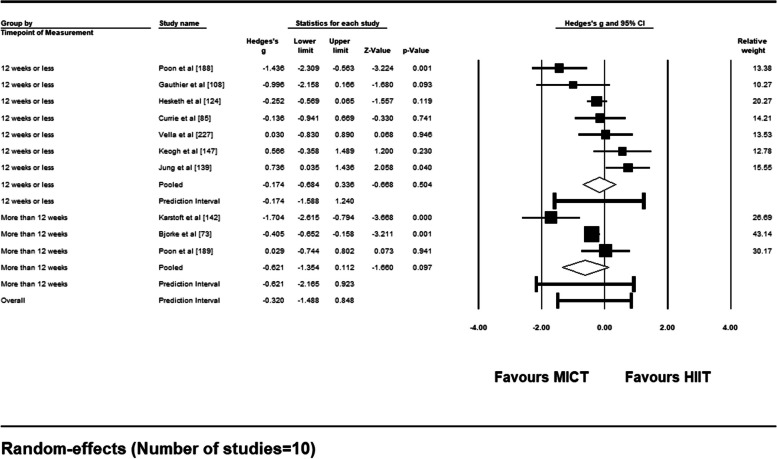
Forest Plot of Sub-Group Analysis (≤12 weeks vs. > 12 weeks) for Adherence Rates to HIIT vs. MICT Interventions

For sub-group analysis based on timepoint of adherence measurement (≤12 weeks vs. > 12 weeks), 7 included studies measured adherence ≤12 weeks post-intervention. Summary of results for this sub-group analysis can be found in Fig. 15. Mean effect sizes for both sub-groups were non-significant (*ps* > .05) and comparison between the two yielded no difference in adherence rates between measurements ≤12 weeks and > 12 weeks post-intervention [*Q* (1) = 0.961, *p* = .327].

**Fig. 16:**
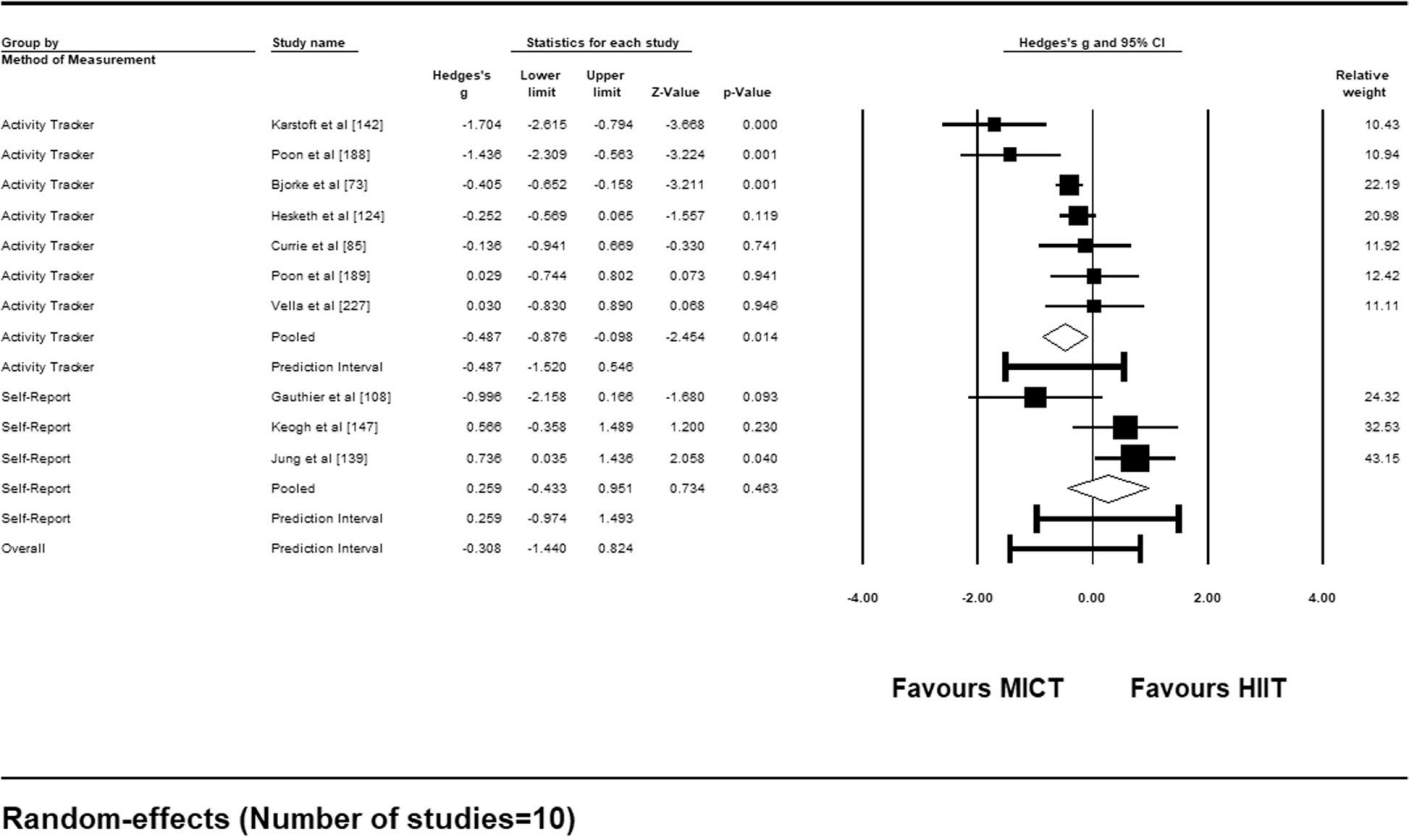
Forest Plot of Sub-Group Analysis (activity tracker vs. self-report) for Adherence Rates to HIIT vs. MICT Interventions

The results of the sub-group analysis based on the method of adherence measurement (activity tracker vs. self-report) can be found as a forest plot (Fig. 16). Seven of the 10 studies measured adherence with activity trackers (i.e., heart rate monitor, wearable watch, accelerometer). The remaining 3 studies measured adherence through self-report measures. For studies that measured adherence through self-report, mean effect size was not significant [Hedge’s *g* = 0.259 (95% CI: − 0.433 – 0.951), *p* = .46] with a prediction interval of − 0.974 to 1.493. Mean effect size for the studies that used activity trackers significantly favored the MICT interventions [Hedge’s *g* = − 0.487 (95% CI: − 0.876 – − 0.098), *p* = .014] with a prediction interval of − 1.520 to 0.546. The comparison between the two mean effect sizes showed no significant difference in adherence rates depending on whether adherence was measured using activity trackers or self-report measures [*Q* (1) = 3.391, *p* = .066].

### GRADE quality appraisal

Quality appraisal of the cumulative body of evidence for each outcome variable can be found in the GRADE Evidence Profile (Table 4). For the outcome variable of compliance to supervised HIIT vs. MICT interventions, no serious concerns were noted in four of the five domains appraised. Specifically, most included studies were randomized controlled trials, a relatively small proportion of included studies were assessed to have high risk of bias, heterogeneity values (i.e. *I*^*2*^, *τ*^2^) were moderate and did not significantly impact mean effect size estimates, sufficient sample sizes for each condition provided confidence in the findings, and no publication bias was detected. When appraising the directness of the evidence, some concerns were raised due to the potential of interventions being delivered differently in different settings based on the diversity of the interventions included in the analysis. For example, type, duration, mode of delivery, and intensity of exercise varied in each study, some of which may have influenced compliance to the intervention. As such, quality of the evidence was downgraded one level due to indirectness, resulting in an overall moderate certainty rating.

**Table 4:** GRADE Evidence Profile for compliance and adherence rates to high-intensity interval training vs. moderate-intensity continuous training interventions among insufficiently active individuals

Certainty assessment							№ of patients		Absolute Effect (95% CI)	Certainty
№ of studies	Study design	Risk of bias	Inconsistency	Indirectness	Imprecision	Other considerations	HIIT	MICT		
**Compliance**										
65	Randomized trials	Not serious^a^	Not serious^b^	Serious^c^	Not serious^d^	No publication bias detected	1249	1230	SMD **0.015 SD higher** (0.088 lower to 0.118 higher)	⨁⨁⨁◯ Moderate
**Adherence**										
10	Randomized trials	Serious^e^	Serious^f^	Serious^g^	Serious^h^	No publication bias detected	301	282	SMD **0.313 SD lower** (0.681 lower to 0.056 higher)	⨁◯◯◯ Very low

*CI* confidence interval, *SMD* standardized mean difference

^a^ 15.4% of included studies were assessed to have high risk of bias, the results of which did not significantly differ from studies with low/moderate risk of bias, so quality of evidence was not downgraded

^b^ Moderate heterogeneity in findings with relatively low between-study variance

^c^ Diversity in interventions delivered decreases the directness of the comparisons between groups

^d^ Adequate sample size gives confidence in findings

^e^ 20% of included studies were assessed to have a high risk of bias, posing some concerns over the design and/or execution of studies

^f^ Substantial heterogeneity in findings denotes inconsistency in findings

^g^ Diversity in the methods and timepoints of adherence measurement increase indirectness of the outcome variable

^h^ Insufficient sample size per intervention to have confidence in precision of results

For the outcome variable of adherence to unsupervised HIIT vs. MICT interventions, serious concerns were noted for each domain appraised. With the low number of included studies, heterogeneity in findings was high and inconsistent between studies, and a low sample size in each condition coupled with statistically significant sensitivity analyses decrease confidence in the precision and robustness of results. Similar to compliance, diversity in the interventions delivered, timepoints and methods of outcome measurement raise concerns over the directness of the comparisons being made. Taken together, quality of the evidence was rated as very low for the adherence outcome.

## Discussion

The primary and secondary purposes of this systematic review and meta-analyses were to first determine what compliance and adherence rates to supervised and unsupervised HIIT interventions were, respectively, for insufficiently active adults and adults presenting with a medical condition; and second, to determine whether compliance and adherence rates were different between HIIT and MICT interventions in both supervised and unsupervised settings. One-hundred and 88 unique studies were included in this review representing a diversity of populations and HIIT iterations. In congruence with our hypothesis, average compliance rate to supervised HIIT interventions was relatively high (> 89% of sessions attended), suggesting that under controlled settings, HIIT is a viable exercise option for insufficiently active adults and individuals presenting with a medical condition. This is inclusive of varying forms of HIIT, such as traditional 4 × 4-minute intervals, low-volume HIIT, sprint interval training, and so forth. This finding may shed light on whether HIIT is feasible for a largely sedentary population due to concerns over perceived difficulty and/or affective response in supervised settings [21–24].

Based on the 65 studies included in the meta-analysis, compliance rates were not different between supervised HIIT and MICT interventions. These results appear robust as sensitivity analyses suggested no study significantly influenced results and a small risk of publication bias was detected. This non-significant finding alludes to the thought that both HIIT and MICT are viable exercise options in supervised settings among insufficiently active adults and adults presenting with a medical condition. Given that both exercise modalities have been shown to elicit positive physiological benefits [14–18], perhaps providing a choice between the two may prove optimal when developing physical activity recommendations and designing supervised exercise interventions. Quality appraisal of the evidence was rated as moderate due to the diversity in interventions, thus limiting the direct comparisons between standardized HIIT and MICT modalities free from influences of exercise time, equipment choice and intensity. It would be interesting for future syntheses to compare compliance and adherence rates to different HIIT and MICT exercise protocols to determine whether optimal protocols exist that elicit the highest completion rates. The vast diversity of protocols found in this review precluded such formal analyses.

Average adherence rates to unsupervised, real-world HIIT/MICT interventions were moderate (HIIT:63%; MICT: 68%). This decrease in completion of HIIT and MICT sessions in real-world environments compared to supervised settings may suggest that individual’s behaviors are influenced by one’s knowledge of being directly under observation in a supervised setting, as is the case in randomized controlled trials [241]. Previous research has also indicated social support to be an important determinant of physical activity engagement [242–244]. Nonetheless, considering the minimal amount of external support received in unsupervised interventions, individuals who had never done HIIT and/or MICT before completed over 60% of prescribed sessions, implying such exercise modalities may be well tolerated in this population. Further research may be warranted to explore potential strategies to increase adherence rates to unsupervised HIIT and MICT exercise. For example, support through concurrent mHealth, eHealth, and activity tracker interventions [245–248], the implementation of behavior change techniques [249, 250], and/or the development of unsupervised interventions grounded on theoretical frameworks [251] are just some strategies that have shown promise in improving physical activity behaviour.

Based on the meta-analysis including 10 studies, no statistical difference was found in adherence rates between unsupervised, real-world HIIT and MICT interventions, although there appears to be a notable trend favouring MICT dependent on the method of measuring adherence. However, these results should be interpreted with great caution as concerns over the robustness of the analysis, high heterogeneity between studies, and very low quality of the evidence are apparent. Results from the sensitivity analysis support the need for caution, as the removal of two studies seem to influence results towards a non-significant finding [139, 147]. However, the purpose of a sensitivity analysis is not to discredit the main findings of a meta-analysis, but rather to assess whether such analysis is robust, or whether more research is needed to solidify the pooled effect estimate. Furthermore, due to the sheer diversity in interventions, there are countless confounding variables that cannot be controlled for in a meta-analysis (hence heterogeneity). The only manner in which to address this is by increasing the number of studies in the analysis, and by doing so, eventually diluting the effects of such confounders. Lastly, as it may be the case that certain studies are pulling the pooled effect estimate towards a non-significant finding [139, 147], it may equally be the case that further research would support or thwart the findings from these certain studies. This is the basic statistical concept of a normal sampling distribution, and the only way to confirm the precision of the effect estimate is by increasing the number of studies in the analysis, not by pointing to one or two studies which may or may not be an accurate depiction of the true parameter effect.

There is a clear need for more research to be conducted on adherence to unsupervised HIIT and MICT interventions to increase the confidence in mean effect size estimate and its accompanying confidence interval. Future interventions would greatly benefit from prescribing standardized HIIT and MICT protocols for ease of comparison across studies, as well as consensus on the method and unit of measuring adherence rates. Additionally, more randomized controlled trials comparing adherence rates are needed on insufficiently active but otherwise healthy individuals since none were included in this meta-analysis. Nonetheless, this preliminary evidence suggests that both HIIT and MICT exercise protocols may be viable options for adults presenting with a medical condition in unsupervised, real-world settings.

When considering the method of measuring adherence rates in real-world settings, sub-group analysis may point to differences between self-report and activity tracker measures, with activity trackers favoring higher adherence rates in MICT conditions compared to HIIT, and self-report measures showing similar adherence rates between the two conditions. Although causal conclusions cannot be drawn from this analysis due to the low number of studies aggregated in each sub-group and the lack of control for potential confounding variables, the results observed are interesting and could be due to a couple of factors. A review of reviews summarizes that self-report methods of measuring physical activity have the potential to be inconsistent dependent on the context of implementation and when compared to other forms of measurement [252]. This may be the case in the studies included in this review, and hence the differences observed between self-report measures and wearable activity measures of physical activity. In contrast, it may be the case that the inconsistencies found in this review stem from wearable activity trackers instead of self-report measures, and the inability for older and current trackers to accurately capture higher intensity exercise bouts [252]. Moving forward, it may be best practice to measure adherence rates to unsupervised physical activity in a multitude of ways in any given study, inclusive of self-report and activity trackers, so that cross-examination may be done to provide a more accurate depiction of physical activity intensity and behaviour in unsupervised settings.

### Strengths and limitations

Our systematic review and meta-analyses had a variety of strengths, such as the inclusion of a large number of studies conducted in a variety of settings with different populations across the globe. As such, our findings may be generalizable to most populations of interest. However, it should be noted that due to most included studies being randomized controlled trials, relatively small sample sizes, and substantial heterogeneity of trial design amongst free-living interventions, generalizability should be done with caution. Another strength of this review is the employment of rigorous processes in the database searches, article screening, data retrieval, and reporting phases, which further add to the quality of this review. Furthermore, the use of well-established tools, guidelines, and statistical processes at each stage provides confidence in the results presented.

Despite these strengths, there are several limitations that should be noted. Importantly, our review placed focus on attendance and completion rates of prescribed exercise modalities without considering whether individuals achieved the intensities of such exercises. It could be the case that although compliance and adherence to exercise were relatively high, the distinction between HIIT and MICT could be decreased in instances where individuals were unable to achieve and/or maintain higher-intensity efforts [20]. In congruence with recommendations by Taylor and colleagues [253], future research on exercise implementation should consider both attendance and intensity achievement to determine implementation success. Another limitation is that roughly a quarter of full texts were excluded from this review due to the non-reporting of compliance or adherence. Although we cannot be certain, perhaps studies that reported compliance or adherence rates are more prone to attempt to evoke engagement in their intervention, thus inflating the compliance and adherence rates calculated in this review. Another limitation that may exacerbate the differences in compliance and adherence rates between HIIT and MICT is the standardization of these outcomes to percentage of completed sessions when calculating weighted means and standardized mean differences. Although such standardization allows for ease of comparison between studies, it does not consider the absolute number of exercise sessions prescribed. In rare instances when the number of sessions prescribed are different between groups e.g., [174, 197, 205, 209], the percentage of completed sessions may be inflated when a lower number of sessions are prescribed compared to the other group. For example, Shepherd and colleagues [209] note that although attendance percentage is higher in the HIIT group compared to MICT, the absolute number of sessions completed by the MICT group was greater since they were prescribed more sessions.

The exclusion of grey literature, qualitative studies, and non-peer reviewed studies are also limitations. The information from these other sources may have impacted the results presented, although analyses aiming to detect publication bias were conducted to mitigate such risk. Although not necessarily a limitation of this study, the small number of studies included in the adherence meta-analysis and very low quality of evidence impede concrete conclusions to be made, highlighting the need for more research in this area.

## Conclusions

Results from this systematic review and meta-analyses indicate that compliance rate to supervised HIIT interventions is relatively high (89%) and not significantly different than supervised MICT interventions (92%) among insufficiently active adults and adults presenting with a medical condition. Such information could prove useful when developing physical activity recommendations and exercise interventions. Average adherence rate to unsupervised, real-world HIIT interventions is moderate (63%) and comparable to MICT interventions (68%), although these findings should be interpreted with caution due to the low number of studies, high heterogeneity, and very low quality of evidence. Further research is needed to increase confidence in adherence rate results among these populations, taking into consideration the exercise protocols employed, method of outcome measurement, unit of measurement, and timepoint of measurement. Future research on differences in compliance rates between varying HIIT and MICT protocols could also be of interest to determine whether optimal protocols exist to promote short- and long-term physical activity participation. Lastly, instead of focusing on whether one exercise modality is superior to another for improving free-living physical activity behavior, future research may benefit from focusing on constructs that may impact such behavior, including the use of eHealth, behavior change techniques, and theory in intervention development.

### Supplementary Information

**Additional File 1.** Completed PRISMA 2020 checklist for this systematic review.

**Additional File 2.** Search strategy used in the Medline database during the article retrieval phase of this systematic review.

**Additional File 3.** Data extraction form and accompanying examples used during the data extraction phase of this systematic review.

**Additional File 4.** Table including general information for each included study, such as year of publication, language, number of sites, countries of origin, sources of funding, conflicts of interest, ethical approval, and participant consent.

**Additional File 5.** Table including study design information for each included study, such as type of study, study design, and population of interest (presence of medical condition and level of physical activity).

**Additional File 6.** Table including group allocation information for each included study, such as total sample size, number of arms, allocation ratio, and types of groups included (HIIT, MICT, Control, Others).

**Additional File 7.** Table including intervention characteristics for supervised interventions, such as mean age (SD), percentage of biological sex, intervention settings, number of sessions per week, total number of sessions, description of interventions, and whether interventions included other components (strength training, other exercise, counselling).

**Additional File 8.** Table including information on compliance rates for supervised interventions, such as the method of measurement, unit of measurement, and mean compliance (SD).

## Data Availability

The datasets used and analyzed during the current study are available from the corresponding author on reasonable request.

## Abbreviations

MVPA: moderate-to-vigorous physical activity
HIIT: high-intensity interval training
MICT: moderate-intensity continuous training
HRmax: maximum heart rate
PRISMA: preferred reporting items for systematic reviews and meta-analyses
RoB 2.0: risk of bias tool 2.0
ROBINS-I: risk of bias in non-randomized studies – of interventions
SD: standard deviation
GRADE: grading of recommendations assessment, development and evaluation
