# Supplementary material for: Rates of compliance and adherence to high-intensity interval training: a systematic review and Meta-analyses
Source: Int J Behav Nutr Phys Act. 2023 Nov 21;20:134. doi: 10.1186/s12966-023-01535-w (PMC10664287; doi:10.1186/s12966-023-01535-w)
Supplement: Supplementary file 2 — Additional File 2. Search strategy used in the Medline database during the article retrieval phase of this systematic review. [file 12966_2023_1535_MOESM2_ESM.pdf]

Additional File 2 – Medline Search Strategy

Medline (Ovid) Legend:

- .mp = search term is found within either the title, abstract, or author-supplied keywords of the article.
- / = MeSH heading found in MeSH database.

("high intensity" adj3 train*).mp. OR
("high intensity" adj3 exercis*).mp. OR
("aerobic interval train*").mp. OR
("aerobic circuit train*").mp. OR
("aerobic interval exercis*").mp. OR
("aerobic intermittent exercis*").mp. OR
("aerobic circuit exercis*").mp. OR
("interval train*").mp. OR
("intermittent train*").mp. OR
("circuit train*").mp. OR
("interval exercis*").mp. OR
("intermittent exercis*").mp. OR
("circuit adj2 exercis*").mp. OR
(sprint adj2 train*).mp. OR
(sprint adj2 exercis*).mp. OR
(HIIT).mp. OR
(exp circuit-based exercise/) OR
(exp high-intensity interval training/)

AND

(adher*).mp. OR
(complian*).mp. OR
(participat*).mp. OR
(dropout).mp. OR
(complet*).mp. OR
(retention).mp. OR
(attend*).mp. OR
("lack of uptake").mp. OR
(frequen*).mp. OR
("lost to follow-up").mp. OR
("attendance frequen*").mp. OR
(exp treatment adherence and compliance/) OR
(exp lost to follow-up/) OR
(exp surveys and questionnaires/)
