# Supplementary material for: Rates of compliance and adherence to high-intensity interval training: a systematic review and Meta-analyses
Source: Int J Behav Nutr Phys Act. 2023 Nov 21;20:134. doi: 10.1186/s12966-023-01535-w (PMC10664287; doi:10.1186/s12966-023-01535-w)
Supplement: Supplementary file 3 — Additional File 3. Data extraction form and accompanying examples used during the data extraction phase of this systematic review. [file 12966_2023_1535_MOESM3_ESM.pdf]

Additional File 3 – Data Extraction Form

General Study Information

Language

Please provide the language the article is written in. If other than English, please let Alex know so he can recruit someone else to extract the data.

Title of Study

Provide full title in APA format: capitalize first word; capitalize first word after a colon; capitalize proper nouns.

Example: Rates of compliance and adherence to high-intensity interval training: A systematic review and meta-analysis protocol.

First Author

Authors full name

Example: Mary E. Jung

Other Authors

Provide all other authors in APA format.

Example: Santos, A., Locke, S., Stork, M.

Corresponding Author

Provide corresponding authors full name.

Example: Mary E. Jung

Corresponding Author's e-mail

Provide an e-mail address.

Example: mary.jung@ubc.ca

Year of Publication

Provide the 4 digits representing year.

Example: 2019

Journal of Publication

Provide full name of journal.

Example: Journal of Sports Sciences

Issue Number

Provide numerical digits.

Example: 3

Volume Number

Provide numerical digits.

Example: 10

Page Numbers

Provide a range of numerical digits separated by a dash.

Example: 103-110

Publisher

Provide the name of the publisher of the journal.

Example: Wiley & Sons

DOI

Provide the url of the article.

Example: https://doi.org/10.1186/s13643-020-01301-0

Number of Sites

Was the research conducted at a single place or were there multiple places/countries involved?

- Single-Centre
- Multi-centre

If multi-centre, countries where each centre was located (list all)

If research was conducted in multiple countries list them.

Example:

- *Canada*

- *United States*

- *Germany*

Country where research was conducted

If research was conducted in only one country, provide the country.

Example: Canada

Funding Sources (if any)

List all funding sources for this research study including grant identification number.

Example: - Canadian Institutes of Health Research Operating Grant (CH00067)

Conflict of interest

Did the authors report any conflicts of interest? This can be found either in-text, at the title page, or at the end of the manuscript.

- Yes
- No
- Not Mentioned

If conflicts of interest were present, copy and paste them here.

Example: RHS was funded by Biopharma Inc. from 2002 to 2005.

Ethics Approval

Did the authors specify whether ethical approval was obtained for this study and/or whether they followed the Declaration of Helsinki Ethics Code?

- Yes
- No

If ethical approval was granted, whom was it granted by?

Example: Ethical approval was granted by the research ethics board of the Children's Hospital of Louisiana.

Data available

Do the authors provide a statement as to how others can access the data used in this research?

- Upon Request
- As Supplementary Material
- As an open-sourced publication
- No

Informed Consent from Participants

Do the authors state whether they received informed consent from their participants?

- Yes
- No

If informed consent was received, how did they get consent?

Example: Participants provided informed consent during baseline via paper.

Study Characteristics

Type of Study

Was this a prospective study (i.e. participants were recruited and intervention then commenced) or a retrospective study (i.e. data was already collected and authors are now analyzing it)?

- Prospective
- Retrospective

Study Design

Do the authors specify the study design? Usually found at the beginning of the methodology.

- Randomized Controlled Trial
- Observational Trial
- Cross-Over Trial
- Case Series
- Other

Participants

Population of interest

Copy and paste the authors' description of the population.

Example: Patients in a cardiac rehabilitation clinic

Co-morbidity

Do the participants suffer from a medical condition?

- Yes
- No

State the medical condition.

Example: Asthma

Level of physical activity

Do the authors state what level of physical activity their participants were?

Our definition of insufficiently active:

Not meeting recommended physical activity guidelines of 150 minutes of moderate to vigorous physical activity per week.

- Insufficiently Active
- Recreationally Active
- Active
- Combination of the above
- Not mentioned

If insufficiently active, provide their definition.

Copy and paste their definition of insufficiently active if provided.

Example: Participants were deemed eligible if they were inactive (i.e. not meeting 150 minutes of MVPA/week).

If a combination of the above, state which ones.

If there were different groups with different levels of physical activity, what were they?

Example: A group deemed inactive and a group of endurance athletes.

Inclusion Criteria (list all)

Copy and paste the authors' inclusion criteria.

Example: - Between the ages of 30 to 65 years

- Inactive

- Overweight

- Not currently engaged in other treatments

Exclusion Criteria (list all)

Copy and paste the authors' exclusion criteria.

Example: - Pregnant

- *On insulin medication*

- *Engaged in another study*

Group Characteristics

Total Sample Size

Provide the numeric digits of the total sample size at the beginning of the study.

Example: 382

Number of arms

How many groups were participants divided into?

Example: 3

Allocation Ratio

What ratio were participants divided into these groups?

Example: 1:1:2 ratio

HIIT Group?

Was one of the groups a HIIT group?

- Yes
- No

MICT Group?

Was one of the groups a MICT group?

- Yes
- No

Control Group?

Was one of the groups a control group?

- Yes
- No

Other Group(s)?

Was one of the groups a different group other than HIIT, MICT, or control?

- Yes
- No

If yes, state the group(s)

Example: Circuit Training Group; Long-Interval HIIT Group; Soccer Group

HIIT Group/MICT Group/Other Group

Sample Size

How many participants were placed in this group at the beginning of the intervention?

Example: 122

Age Mean

What is the mean (or median) age for this group specifically?

Example: 46.7

Age Standard Deviation

What is the mean age's standard deviation for this group specifically?

Example: (3.4)

Biological Sex

Did the authors report percentages of biological sex? (the biological sex they were given at birth; male; female)

- Yes
- No

Number of male participants

Out of the total group sample, how many were identified as male (biological sex)?

Example: 68

%Male

Divide the number of male participants by the total group sample.

Example: 56%

Gender

Did the authors report on participants' gender? (the gender the participants identifies with at a given point in time; female, male, intersex, trans)

- Yes
- No

Gender Options (list all)

If gender was reported, which genders were included as options?

Example: - male

- female

- intersex

- trans

Percentage of each gender in group (list all)

For each of the gender options above, what percentages were reported for each?

Example: - 25%

Supervised Sessions

Was there a portion of the intervention that was supervised by someone?

- Yes
- No

If yes, Who Did Supervision? If no, type N/A

Example: Sessions were supervised by a certified exercise physiologist.

Intervention Setting

Was the intervention performed individually or in a group setting? Was it a combination of both?

- Individual
- Group
- Mixed

If group setting, provide description

If intervention was in a group setting, copy and paste the description of the setting.

Example: Participants exercised in a group of 5-10 at the local gym.

Number of sessions per week

How many sessions per week did participants engage during the supervised portion of the intervention?

Example: Three times per week

Weeks of Intervention

How many weeks did the supervised portion of the intervention last?

Example: 12 weeks

Duration of Sessions

How long did each supervised exercise session last?

Example: Participants exercised for a total of 45 minutes each session.

Total Number of Sessions

How many supervised exercise sessions in total did participants have to partake in?

Example: A total of 36 sessions.

Type of Aerobic Activity

Copy and paste the modality in which activity was performed.

Examples: - Participants exercised on a cycle ergometer.

- Participants had the choice of exercising on a treadmill, bicycle or elliptical.

Warm-up Period

How long did participants warm-up for?

Example: - 5-minute warm-up

- No warm-up period

Warm-up Intensity

At what intensity was the warm-up performed?

Examples: - 50% of Vo2peak

- Not mentioned

Cool-down Period

How long did participants cool-down for?

Example: - 5-minute cool-down

- no cool-down period

Work-period Description (including FITT Principles)

Copy and paste the authors' description of the exercise intervention.

Example: Participants exercised 4 minutes at 90% of VO2peak interspersed by 3-minutes of active recovery at 50% VO2peak on a treadmill.

Intensity range (including units)

What was the range in which participants exercised?

Example: - >80% HRmax

- 85-95% VO2peak

- RPE rating above 16

Was Intensity Measured?

Did the authors somehow measure intensity during the exercise?

- Yes
- No

Method of Intensity Measurement

If intensity was measured, how did the authors measure intensity?

Example: - HR monitoring throughout

- RPE rating at the end of each interval

Personnel Measuring Intensity

If intensity was measured, who collected the data?

Example: - HR monitor measurements were collected by the certified exercise physiologist supervising each exercise session.

Frequency of Intensity Measurement

How often was intensity measured during the exercise sessions?

Example: - HR monitor measurements were taken every minute.

- RPE rating was taken at the end of each interval.

Strength Training Component

Was there a strength training component in addition to the aerobic exercise as part of the intervention?

- Yes
- No

Strength Training Description

Copy and paste the authors' description of the strength training component.

Example: In addition to aerobic exercise, participants engaged in strength training twice a week throughout the intervention. Strength exercises included upper and lower body exercise at 70% 1RM capacity.

Other Exercise Components

Were there other exercise components in addition to the aerobic exercise as part of the intervention that were not strength training?

- Yes
- No

Other Exercise Description

Copy and paste the authors' description of the other exercises.

Example: - In addition to aerobic exercise, participants engaged in group yoga led by a certified professional once a week.

Counselling Component

Was there a counselling component in addition to the exercise of the intervention?

- Yes
- No

Counselling Component Description

Copy and paste the authors' description of the counselling component.

Example: Participants met with a counsellor three times a week for 30 minutes to discuss nutritional and physical activity goals.

Behaviour Change Techniques

Do the authors mention the use of any behaviour change techniques as part of the intervention?

- Yes
- No

Behaviour Change Techniques Used

Copy and paste the authors' description or list of behaviour change techniques used in the intervention.

Example: Behaviour change techniques used during the counselling portion of the intervention included goal-setting and self-regulation strategies.

Unsupervised Sessions

Was there an unsupervised exercise portion of the intervention?

- Yes
- No

Number of prescribed sessions per week

If unsupervised exercise was prescribed, how many exercise sessions per week were they prescribed?

Example: Participants were instructed to continue exercising 3 times per week for the subsequent 12 months.

Type of Aerobic Activity

What was the type of activity prescribed for the unsupervised sessions?

Examples: - Participants were instructed to walk at a moderate to vigorous intensity.

- Participants were prescribed 3 sessions of HIIT per week on a treadmill.

Method of Intensity Measurement

How was intensity of unsupervised sessions measured?

Examples: - Participants were provided with a wearable HR monitor that the research team had access to the data.

- Participants were instructed to monitor their intensity by using the talk test.

- Not mentioned.

Control Group

Sample Size

How many participants were placed in this group at the beginning of the intervention?

Example: 122

Age Mean

What is the mean (or median) age for this group specifically?

Example: 46.7

Age Standard Deviation

What is the mean age's standard deviation for this group specifically?

Example: (3.4)

Biological Sex

Did the authors report percentages of biological sex? (the biological sex they were given at birth; male; female)

- Yes
- No

Number of Male Participants

Out of the total group sample, how many were identified as male (biological sex)?

Example: 68

%Male

Divide the number of male participants by the total group sample.

Example: 56%

Gender

Did the authors report on participants' gender? (the gender the participants identifies with at a given point in time; female, male, intersex, trans)

- Yes
- No

Gender options (list all)

If gender was reported, which genders were included as options?

Example: - male

- *female*

- *intersex*

- *trans*

Percentage of each gender in group (list all)

For each of the gender options above, what percentages were reported for each?

Example: - 25%

Waitlist Control

Was the control group considered a waitlist control (participants in the control group would get the intervention at the end of the study)

- Yes
- No

Usual Care

Were participants in the control group provided usual care (went about normal treatment without any sort of intervention)?

- Yes
- No

Usual Care Description

If usual care was used, copy and paste the authors' description of usual care.

Example: Participants in the control received usual care consisting of cardiac rehabilitation medication at the hospital.

Control Protocol

Were participants in the control group provided with something other than usual care?

- Yes
- No

Control Protocol Description

If a protocol was used with the control group, copy and paste the authors' description of the control protocol.

Example: - Participants in the control group were only given the counselling portion of the intervention.

- Participants in the control group attended a 30-minute seminar about recommended physical activity guidelines.

Outcome Characteristics

Compliance

Did the authors report compliance to supervised exercise as an outcome?

Definition of compliance: the frequency of attendance, in percentage, to exercise sessions of a program

- Yes
- No

Definition of Compliance

Copy and paste the authors' definition of compliance.

Example: Attendance rate to the 36 sessions was used to assess program adherence.

**NOTE - A lot of articles use compliance and adherence interchangeably. Compliance applies to supervised sessions only.

Method of Compliance Measurement

What method did the authors use to measure compliance?

- Attendance Rate
- Reaching Target Intensity
- Other

If other, copy and paste how authors measured compliance.

Example: Participants were deemed compliant if they finished a minimum of 80% of sessions.

Steps Taken to Ensure Compliance

Copy and paste any protocols the research team implemented to make up for any missed exercise sessions.

Example: When a session was missed, participants were scheduled for a make-up session within the next 2 weeks to ensure all sessions were completed.

Unit of Measurement

What is the unit of measurement of compliance?

Example: Percentage of attendance

Timeframe in which Compliance was Measured For

For what period did compliance apply?

Example: Attendance rate was calculated as the percentage of sessions attended during the 12 week intervention period.

Adherence

Was adherence measured as one of the outcomes?

Definition of adherence: the number of minutes or sessions per week engaged in purposeful MVPA in unsupervised, unstructured environments after engaging in a program. Both self-report and wearable measures count.

- Yes
- No

Definition of Adherence

Copy and paste the authors' definition of adherence.

Example: self-reported exercise adherence and physical activity, objectively measured physical activity.

Method of Adherence Measurement

How was adherence measured in the study?

- Accelerometer
- Activity Tracker
- Self-Report
- Other

What other method of measuring adherence did the authors use?

Example: Adherence was measured with accelerometers AND self-report data.

Unit of Measurement

What is the unit of measurement of adherence?

Example: - Number of exercise sessions engaged in per week.

- Number of MVPA minutes engaged in over a 7-day period.

Time Period of Measurement

How long was each measure of adherence taken for?

Example: - Accelerometer data was taken for a 7-day period.

- Participants were asked to recall their physical activity patterns in the last month.

Time Points of Measurement (list all)

What timepoints was adherence measured on?

Example: - Adherence was measured before, after, 6-months, and 12-months after the supervised exercise intervention.

Results

Statistical Test for Dealing with Missing Data

Did the authors use a per-protocol analysis or intention to treat analysis? How did they handle missing data? Copy and paste the authors' discussion on this.

Example: An intention to treat analysis was used for this study. 13% of data was missing for the primary outcome variable. Multiple imputation method was used to account for missing data under the assumption that data was missing at random.

HIIT Group/MICT Group/Control Group/Other Group

Number of Dropouts

The numerical digit for how many participants dropped out at any point during the intervention.

Example: 4

Definition of dropout: Participants willingly stopped the program for any given reason.

Percentage of Dropouts

The number of dropouts divided by the original total number of participants in this group.

Example: 4/66 = 6.1%

Number of Lost to Follow-Ups

The numerical digit for how many participants were lost to follow-up at any point during the intervention.

Example: 6

Definition of lost to follow-up: Participants were contacted multiple times but were non-responsive.

Percentage of Lost to Follow-Ups

The number of lost to follow-ups divided by the original total number of participants in this group.

Example: 6/66 = 11%

Compliance Result

The numerical value of the compliance measure.

Example: 96%

Compliance Standard Deviation

The standard deviation of the compliance measure.

Example: (4.2)

Compliance Effect Size

If given, the effect size of the compliance measure.

Example: d = .20

Compliance 95% CI (standard Error)

If given, the 95% confidence interval of the compliance measure.

Example: 88%-97%

Adherence Results (include all timepoints)

Copy and paste the results of the adherence measure from the study for this group for each time point.

Example: At the 6-month follow-up, participants in the HIIT group reported exercising an average of 3.2 times per week at a moderate-to-vigorous intensity.

Adherence Standard Deviation (include all timepoints)

The standard deviation of the adherence measure results if given.

Example: (1.1)
