# Supplementary material for: Rates of compliance and adherence to high-intensity interval training: a systematic review and Meta-analyses
Source: Int J Behav Nutr Phys Act. 2023 Nov 21;20:134. doi: 10.1186/s12966-023-01535-w (PMC10664287; doi:10.1186/s12966-023-01535-w)
Supplement: Supplementary file 4 — Additional File 4. Table including general information for each included study, such as year of publication, language, number of sites, countries of origin, sources of funding, conflicts of interest, ethical approval, and participant consent. [file 12966_2023_1535_MOESM4_ESM.docx]

**Additional File 4.** General Study Information

| **Study Reference** | **Year of Publication** | **Language** | **Number of Sites** | **Countries of Origin** | **Source of Funding** | **Conflict of Interest** | **Ethical Approval** | **Participant Consent** |
| --- | --- | --- | --- | --- | --- | --- | --- | --- |
| Aamot et al. [54] | 2016 | English | Single-Centre | Norway | Yes | No | Yes | Yes |
| Adams et al. [55] | 2017 | English | Single-Centre | Canada | No | No | Yes | Yes |
| Allen et al. [56] | 2017 | English | Single-Centre | Australia | No | No | Yes | Yes |
| Allen et al. [57] | 2018 | English | Single-Centre | United States | Yes | No | Yes | Yes |
| Allison et al. [58] | 2017 | English | Multi-Centre | Canada | Yes | No | Yes | Yes |
| Alvarez et al. [59] | 2016 | English | Single-Centre | Chile | Yes | No | Yes | Yes |
| Arad et al. [60] | 2020 | English | Single-Centre | United States | Yes | No | Yes | Yes |
| Archila et al. [61] | 2021 | English | Single-Centre | Canada | Yes | Not Mentioned | Yes | Yes |
| Astorino et al. [62] | 2013 | English | Single-Centre | United States | Yes | No | Yes | Yes |
| Atan et al. [63] | 2020 | English | Single-Centre | Turkey | Not Mentioned | Not Mentioned | Yes | Yes |
| Avila-Gandi­a et al. [64] | 2021 | English | Single-Centre | Spain | Yes | No | Yes | Yes |
| Baekkerud et al. [65] | 2016 | English | Single-Centre | Norway | Yes | No | Yes | Yes |
| Bang-Kittilsen et al. [66] | 2021 | English | Single-Centre | Norway | Yes | No | Yes | Yes |
| Banitalebi et al. [67] | 2019 | English | Single-Centre | Iran | Yes | No | Yes | Yes |
| Beetham et al. [68] | 2019 | English | Single-Centre | Australia | Yes | Yes | Yes | Yes |
| Benda et al. [69] | 2015 | English | Single-Centre | Netherlands | Yes | No | Yes | Yes |
| Benham et al. [70] | 2021 | English | Single-Centre | Canada | Yes | No | Yes | Yes |
| Berger et al. [71] | 2006 | English | Single-Centre | United Kingdom | Not Mentioned | Not Mentioned | Yes | Yes |
| Billany et al. [72] | 2022 | English | Single-Centre | United Kingdom | Yes | No | Yes | Yes |
| Bjorke et al. [73] | 2022 | English | Single-Centre | Sweden | Yes | No | Yes | Yes |
| Briggs et al. [74] | 2021 | English | Single-Centre | United States | Yes | No | Yes | Yes |
| Brobakken et al. [75] | 2020 | English | Single-Centre | Norway | Yes | No | Yes | Yes |
| Cano-Montoya et al. [76] | 2016 | Spanish | Single-Centre | Chile | Yes | No | Yes | Yes |
| Cerini et al. [77] | 2022 | English | Single-Centre | Switzerland | No | No | Yes | Yes |
| Cheema et al. [78] | 2015 | English | Single-Centre | Australia | Yes | No | Yes | Yes |
| Ciolac et al. [79] | 2010 | English | Single-Centre | Brazil | Yes | No | Yes | Yes |
| Coletta et al. [80] | 2019 | English | Single-Centre | United States | Yes | No | Yes | Yes |
| Connolly et al. [81] | 2016 | English | Single-Centre | Denmark | Yes | No | Yes | Yes |
| Conraads et al. [82] | 2014 | English | Multi-Centre | Belgium | Yes | No | Yes | Yes |
| Cooke et al. [83] | 2022 | English | Single-Centre | Australia | No | No | Yes | Yes |
| Cooper et al. [84] | 2016 | English | Single-Centre | Australia | Yes | No | Yes | Yes |
| Currie et al. [85] | 2013 | English | Single-Centre | Canada | Yes | No | Yes | Yes |
| Currie et al. [86] | 2015 | English | Single-Centre | Canada | Yes | Not Mentioned | Yes | Yes |
| D’Amuri et al. [87] | 2021 | English | Single-Centre | Italy | Yes | No | Yes | Yes |
| Damme et al. [88] | 2022 | English | Single-Centre | United States | Yes | No | Yes | Yes |
| Deraas et al. [89] | 2021 | English | Single-Centre | Norway | Yes | No | Yes | Yes |
| Devin et al. [90] | 2016 | English | Single-Centre | Australia | Yes | No | Yes | Yes |
| Devin et al. [91] | 2018 | English | Single-Centre | Australia | Yes | No | Yes | Yes |
| Dissing et al. [92] | 2019 | English | Single-Centre | Denmark | Yes | No | Not Mentioned | Yes |
| Dolan et al. [93] | 2016 | English | Single-Centre | Canada | Yes | No | Yes | Yes |
| Dowd et al. [94] | 2022 | English | Single-Centre | Canada | Yes | No | Yes | Yes |
| Egegaard et al. [95] | 2019 | English | Single-Centre | Denmark | Yes | No | Yes | Yes |
| Eichner et al. [96] | 2019 | English | Single-Centre | United States | Yes | No | Yes | Yes |
| Ellingsen et al. [97] | 2017 | English | Multi-Centre | Belgium | Yes | Yes | Yes | Yes |
| Elmer et al. [98] | 2016 | English | Single-Centre | United States | Yes | No | Yes | Yes |
| Emtner et al. [99] | 1996 | English | Single-Centre | Sweden | Yes | Not Mentioned | Yes | Yes |
| Emtner et al. [100] | 1998 | English | Single-Centre | Sweden | Yes | Not Mentioned | Yes | Yes |
| Flaherty et al. [101] | 2014 | English | Single-Centre | United States | No | No | Yes | Yes |
| Flemmen et al. [102] | 2014 | English | Single-Centre | Norway | Yes | No | Yes | Yes |
| Foster et al. [103] | 2015 | English | Single-Centre | United States | Yes | Not Mentioned | Yes | Yes |
| Francois et al. [104] | 2017 | English | Single-Centre | Canada | Yes | No | Yes | Yes |
| Freese et al. [105] | 2014 | English | Single-Centre | United States | Yes | No | Yes | Yes |
| Freitag et al. [106] | 2018 | English | Single-Centre | Germany | No | No | Yes | Yes |
| Freyssin et al. [107] | 2012 | English | Single-Centre | France | Not Mentioned | No | Yes | Yes |
| Gauthier et al. [108] | 2018 | English | Single-Centre | Canada | Yes | No | Yes | Yes |
| Gilbertson et al. [109] | 2019 | English | Single-Centre | United States | Yes | No | Yes | Yes |
| Gildea et al. [110] | 2021 | English | Single-Centre | Ireland | Yes | No | Yes | Yes |
| Gillen et al. [111] | 2014 | English | Single-Centre | Canada | Yes | No | Yes | Yes |
| Gillen et al. [112] | 2016 | English | Single-Centre | Canada | Yes | No | Yes | Yes |
| Gloeckl et al. [113] | 2012 | English | Single-Centre | Germany | No | No | Yes | Yes |
| Golightly et al. [114] | 2021 | English | Single-Centre | United States | Yes | No | Yes | Yes |
| Gorostegi-Anduaga et al. [115] | 2018 | English | Single-Centre | Spain | Yes | No | Yes | Yes |
| Grace et al. [116] | 2018 | English | Single-Centre | Australia | No | No | Yes | Yes |
| Gremeaux et al. [117] | 2012 | English | Single-Centre | Canada | No | No | Not Mentioned | Yes |
| Guillamo et al. [118] | 2018 | English | Single-Centre | Spain | No | No | Yes | Yes |
| Haines et al. [119] | 2020 | English | Single-Centre | United Kingdom | No | No | Yes | Yes |
| Hatle et al. [120] | 2014 | English | Single-Centre | Norway | Yes | No | Yes | Yes |
| Hearon et al. [121] | 2022 | English | Single-Centre | United States | Yes | No | Yes | Yes |
| Heggelund et al. [122] | 2011 | English | Single-Centre | Norway | Not Mentioned | No | Yes | Not Mentioned |
| Heje et al. [123] | 2019 | English | Single-Centre | Denmark | Not Mentioned | Yes | Yes | Yes |
| Hesketh et al. [124] | 2021 | English | Single-Centre | United Kingdom | Yes | No | Yes | Yes |
| Hettchen et al. [125] | 2021 | English | Single-Centre | Germany | Yes | No | Yes | Yes |
| Heydari et al. [126] | 2013 | English | Single-Centre | Australia | No | No | Yes | Yes |
| Higgins et al. [127] | 2016 | English | Single-Centre | United States | No | No | Yes | Yes |
| Hindso et al. [128] | 2022 | English | Single-Centre | Denmark | Yes | No | Yes | Yes |
| Howden et al. [129] | 2018 | English | Single-Centre | United States | Yes | No | Yes | Yes |
| Humphreys et al. [130] | 2022 | English | Single-Centre | United Kingdom | No | No | Yes | Yes |
| Hwang et al. [131] | 2012 | English | Single-Centre | Taiwan | No | No | Yes | Yes |
| Hwang et al. [132] | 2016 | English | Single-Centre | United States | Yes | Not Mentioned | Yes | Yes |
| Iellamo et al. [133] | 2013 | English | Single-Centre | Italy | No | Not Mentioned | Yes | Yes |
| Ivanova et al. [134] | 2022 | English | Single-Centre | Canada | Yes | No | Yes | Yes |
| Izadi et al. [135] | 2018 | English | Single-Centre | Iran | No | No | Yes | Yes |
| Jabbour et al. [136] | 2018a | English | Single-Centre | Canada | Yes | No | Yes | Yes |
| Jabbour et al. [137] | 2018b | English | Single-Centre | Canada | Yes | Not Mentioned | Yes | Yes |
| Jakobsen et al. [138] | 2011 | English | Single-Centre | Denmark | No | Not Mentioned | Yes | Yes |
| Jung et al. [139] | 2015 | English | Single-Centre | Canada | Yes | No | Yes | Yes |
| Jung et al. [26] | 2020 | English | Single-Centre | Canada | Yes | Yes | Yes | Yes |
| Kang et al. [140] | 2021 | English | Single-Centre | Canada | Yes | No | Yes | Yes |
| Karlsen et al. [141] | 2008 | English | Single-Centre | Norway | Yes | Not Mentioned | Yes | Yes |
| Karstoft et al. [142] | 2014 | English | Single-Centre | Denmark | Yes | No | Yes | Yes |
| Kaur et al. [143] | 2021 | English | Single-Centre | Canada | Yes | No | Yes | Yes |
| Keating et al. [144] | 2014 | English | Single-Centre | Australia | Yes | Yes | Yes | Yes |
| Keating et al. [145] | 2022 | English | Single-Centre | Spain | Not mentioned | Not Mentioned | Yes | Yes |
| Kemmler et al. [146] | 2014 | English | Single-Centre | Germany | Yes | No | Yes | Yes |
| Keogh et al. [147] | 2018 | English | Single-Centre | Australia | No | Yes | Yes | Yes |
| Keteyian et al. [148] | 2014 | English | Multi-Centre | United States | No | No | Yes | Yes |
| Keytsman et al. [149] | 2018 | English | Single-Centre | Belgium | No | No | Yes | Yes |
| Kiel et al. [150] | 2022 | English | Multi-Centre | Norway | Yes | No | Yes | Yes |
| Klonizakis et al. [151] | 2014 | English | Single-Centre | United Kingdom | No | No | Yes | Yes |
| Knowles et al. [152] | 2015 | English | Single-Centre | United Kingdom | Not Mentioned | Not Mentioned | Yes | Yes |
| Kong et al. [153] | 2016 | English | Single-Centre | China | Yes | No | Yes | Yes |
| Lanzi et al. [154] | 2015 | English | Single-Centre | Italy | No | No | Yes | Yes |
| Lee et al. [155] | 2020 | English | Single-Centre | Australia | Yes | Yes | Yes | Yes |
| Lee et al. [156] | 2021 | English | Single-Centre | United States | Yes | No | Yes | Yes |
| Locke et al. [157] | 2018 | English | Single-Centre | Canada | No | No | Yes | Yes |
| Lunt et al. [25] | 2014 | English | Single-Centre | New Zealand | Yes | Yes | Yes | Yes |
| Lyall et al. [158] | 2022 | English | Single-Centre | United Kingdom | Yes | Not Mentioned | Yes | Yes |
| MacDonald et al. [159] | 2021 | English | Single-Centre | United States | Yes | Yes | Yes | Yes |
| MacLean et al. [160] | 2018 | English | Single-Centre | United Kingdom | Yes | No | Yes | Yes |
| Madsen et al. [161] | 2015 | English | Single-Centre | Denmark | Yes | No | Yes | Yes |
| Madssen et al. [162] | 2014a | English | Single-Centre | Norway | Yes | No | Yes | Yes |
| Madssen et al. [163] | 2014b | English | Single-Centre | Norway | Yes | No | Yes | Yes |
| Martin et al. [164] | 2021 | English | Single-Centre | Switzerland | No | No | Yes | Yes |
| Martins et al. [165] | 2016 | English | Single-Centre | Norway | Yes | No | Yes | Yes |
| Matsuo et al. [166] | 2013 | English | Single-Centre | Japan | Yes | No | Yes | Yes |
| Mendelson et al. [167] | 2022 | English | Single-Centre | France | Yes | No | Yes | Yes |
| Metcalfe et al. [168] | 2012 | English | Single-Centre | United Kingdom | Yes | No | Yes | Yes |
| Metcalfe et al. [169] | 2016 | English | Single-Centre | United Kingdom | Yes | No | Yes | Yes |
| Metcalfe et al. [170] | 2020 | English | Multi-Centre | Scotland, United Kingdom | Yes | No | Yes | Yes |
| Midtgaard et al. [171] | 2013 | English | Single-Centre | Denmark | Yes | No | Yes | Not Mentioned |
| Mijwel et al. [172] | 2018 | English | Single-Centre | Sweden | Yes | No | Yes | Yes |
| Moholdt et al. [173] | 2009 | English | Single-Centre | Norway | Yes | No | Yes | Yes |
| Moholdt et al. [174] | 2012 | English | Single-Centre | Norway | Yes | No | Yes | Yes |
| Munk et al. [175] | 2009 | English | Single-Centre | Norway | Yes | No | Yes | Yes |
| Nikseresht et al. [176] | 2016 | English | Single-Centre | Iran | Yes | No | Yes | Yes |
| Nilsson et al. [177] | 2019 | English | Single-Centre | Norway | Yes | Yes | Yes | Yes |
| Northey et al. [178] | 2018 | English | Single-Centre | Australia | No | Not Mentioned | Yes | Yes |
| Nybo et al. [179] | 2010 | English | Single-Centre | Denmark | No | Not Mentioned | Yes | Yes |
| Nytroen et al. [180] | 2012 | English | Single-Centre | Norway | Yes | No | Yes | Yes |
| Nytroen et al. [181] | 2019 | English | Multi-Centre | Denmark | Yes | Not Mentioned | Yes | Yes |
| Olsen et al. [182] | 2015 | English | Single-Centre | Denmark | Yes | No | Yes | Yes |
| Papadopoulos et al. [183] | 2021 | English | Single-Centre | Canada | Yes | Yes | Yes | Yes |
| Pattyn et al. [184] | 2016 | English | Single-Centre | Belgium | Yes | No | Yes | Yes |
| Pedersen et al. [185] | 2015 | English | Single-Centre | Denmark | Yes | No | Yes | Yes |
| Phillips et al. [186] | 2017 | English | Multi-Centre | United Kingdom | Yes | Yes | Yes | Yes |
| Piraux et al. [187] | 2022 | English | Single-Centre | Belgium | Yes | No | Yes | Yes |
| Poon et al. [188] | 2020 | English | Single-Centre | Hong Kong | No | No | Yes | Yes |
| Poon et al. [189] | 2022 | English | Single-Centre | Hong Kong | Yes | Not Mentioned | Yes | Yes |
| Rakobowchuk et al. [190] | 2013 | English | Single-Centre | England | Yes | Not Mentioned | Yes | Yes |
| Reljic et al. [191] | 2018 | English | Single-Centre | Germany | No | No | Yes | Yes |
| Reljic et al. [192] | 2022 | English | Single-Centre | Germany | Yes | No | Yes | Yes |
| Robinson et al. [193] | 2015 | English | Single-Centre | Canada | Yes | No | Yes | Yes |
| Rolid et al. [194] | 2020 | English | Multi-Centre | Scandinavia | Yes | No | Yes | Yes |
| Romain et al. [195] | 2018 | English | Single-Centre | Canada | Yes | Not Mentioned | Yes | Yes |
| Rowan et al. [196] | 2017 | English | Single-Centre | Canada | Yes | Not Mentioned | Yes | Yes |
| Roxburgh et al. [197] | 2014 | English | Single-Centre | New Zealand | No | Not Mentioned | Yes | Yes |
| Roy et al. [198] | 2018 | English | Single-Centre | New Zealand | Yes | No | Yes | Yes |
| Ruffino et al. [199] | 2016 | English | Single-Centre | United Kingdom | Yes | No | Yes | Yes |
| Rustad et al. [200] | 2012 | English | Single-Centre | Norway | Yes | No | Yes | Yes |
| Saanijoki et al. [201] | 2015 | English | Single-Centre | Finland | No | Not Mentioned | Yes | Yes |
| Safiyari-Hafizi et al. [202] | 2016 | English | Single-Centre | Canada | Yes | No | Yes | Yes |
| Sargeant et al. [203] | 2018 | English | Single-Centre | United Kingdom | Yes | Yes | Yes | Yes |
| Sawyer et al. [204] | 2016 | English | Single-Centre | United States | Yes | No | Yes | Yes |
| Schmitt et al. [205] | 2016 | English | Single-Centre | Germany | Not Mentioned | No | Yes | Yes |
| Schulz et al. [206] | 2018 | English | Single-Centre | Germany | No | No | Yes | Yes |
| Scott et al. [207] | 2019 | English | Single-Centre | United Kingdom | No | No | Yes | Yes |
| Shenouda et al. [208] | 2017 | English | Single-Centre | Canada | Yes | No | Yes | Yes |
| Shepherd et al. [209] | 2015 | English | Single-Centre | United Kingdom | Yes | No | Yes | Yes |
| Sim et al. [210] | 2015 | English | Single-Centre | Australia | Yes | No | Yes | Yes |
| Simonsen et al. [211] | 2020 | English | Single-Centre | Denmark | Yes | No | Yes | Yes |
| Smith-Ryan et al. [212] | 2016 | English | Single-Centre | United States | Yes | No | Yes | Yes |
| Smith-Ryan et al. [213] | 2021 | English | Single-Centre | United States | Yes | Not Mentioned | Yes | Yes |
| Sogaard et al. [214] | 2017 | English | Single-Centre | Denmark | Yes | No | Yes | Yes |
| Stavrinou et al. [215] | 2019 | English | Single-Centre | Greece | No | No | Yes | Yes |
| Sveaas et al. [216] | 2017 | English | Single-Centre | Norway | No | No | Yes | Yes |
| Taylor et al. [217] | 2020 | English | Single-Centre | Australia | Yes | No | Yes | Yes |
| Terada et al. [218] | 2012 | English | Single-Centre | Canada | Yes | No | Yes | Yes |
| Tew et al. [219] | 2019 | English | Multi-Centre | England | Yes | No | Yes | Yes |
| Tjonna et al. [220] | 2008 | English | Multi-Centre | Norway | Yes | No | Yes | Yes |
| Toennesen et al. [221] | 2018 | English | Single-Centre | Denmark | No | No | Yes | Yes |
| Tong et al. [222] | 2018 | English | Single-Centre | China | Yes | No | Yes | Yes |
| Tschentscher et al. [223] | 2016 | English | Single-Centre | Austria | No | No | Yes | Yes |
| Tsirigkakis et al. [224] | 2021 | English | Single-Centre | Greece | Yes | No | Yes | Yes |
| Turri-Silva et al. [225] | 2021 | English | Single-Centre | Brazil | Yes | No | Yes | Yes |
| Valent et al. [226] | 2009 | English | Single-Centre | Netherlands | No | Not Mentioned | Yes | Yes |
| Vella et al. [227] | 2017 | English | Single-Centre | United States | Yes | No | Yes | Yes |
| Verbrugghe et al. [228] | 2018 | English | Single-Centre | Netherlands | No | No | Yes | Yes |
| Verbrugghe et al. [229] | 2019 | English | Single-Centre | Belgium | Yes | No | Yes | Yes |
| Vestergaard et al. [230] | 2022 | English | Single-Centre | Denmark | Yes | No | Yes | Yes |
| Vidal-Almela et al. [231] | 2022 | English | Single-Centre | Canada | Yes | No | Yes | Yes |
| Way et al. [232] | 2020 | English | Single-Centre | Canada | Yes | No | Yes | Yes |
| Weng et al. [233] | 2013 | English | Single-Centre | China | Yes | No | Yes | Yes |
| Willoughby et al. [234] | 2016 | English | Single-Centre | Canada | Yes | No | Yes | Yes |
| Wilson et al. [235] | 2019 | English | Single-Centre | New Zealand | Yes | No | Yes | Yes |
| Winding et al. [236] | 2018 | English | Single-Centre | Denmark | Yes | No | Yes | Yes |
| Wormgoor et al. [237] | 2018 | English | Single-Centre | New Zealand | Not Mentioned | No | Yes | Yes |
| Zhang et al. [238] | 2017 | English | Multi-Centre | Australia | Yes | No | Yes | Yes |
| Zisko et al. [239] | 2015 | English | Single-Centre | Norway | Yes | No | Yes | Yes |
