# Supplementary material for: Rates of compliance and adherence to high-intensity interval training: a systematic review and Meta-analyses
Source: Int J Behav Nutr Phys Act. 2023 Nov 21;20:134. doi: 10.1186/s12966-023-01535-w (PMC10664287; doi:10.1186/s12966-023-01535-w)
Supplement: Supplementary file 5 — Additional File 5. Table including study design information for each included study, such as type of study, study design, and population of interest (presence of medical condition and level of physical activity). [file 12966_2023_1535_MOESM5_ESM.docx]

**Additional File 5.** Study Design Information

| **Study Reference** | **Type of Study** | **Study Design** | **Medical Condition** | **Level of Physical Activity** |
| --- | --- | --- | --- | --- |
| Aamot et al. [54] | Prospective | Randomized Controlled Trial | Myocardial Infarction | Not mentioned |
| Adams et al. [55] | Prospective | Randomized Controlled Trial | Testicular Cancer | Not mentioned |
| Allen et al. [56] | Prospective | Randomized Controlled Trial | -- | Insufficiently Active |
| Allen et al. [57] | Prospective | Randomized Controlled Trial | -- | Insufficiently Active |
| Allison et al. [58] | Prospective | Observational Trial | -- | Insufficiently Active |
| Alvarez et al. [59] | Prospective | Randomized Controlled Trial | Type 2 Diabetes | Insufficiently Active |
| Arad et al. [60] | Retrospective | Randomized Controlled Trial | Obesity | Insufficiently Active |
| Archila et al. [61] | Prospective | Randomized Controlled Trial | -- | Insufficiently Active |
| Astorino et al. [62] | Prospective | Randomized Controlled Trial | -- | Insufficiently Active |
| Atan et al. [63] | Prospective | Randomized Controlled Trial | Fibromyalgia | Insufficiently Active |
| Avila-Gandi­a et al. [64] | Prospective | Randomized Controlled Trial | Hypertension | Insufficiently Active |
| Baekkerud et al. [65] | Prospective | Randomized Controlled Trial | Obesity | Insufficiently Active |
| Bang-Kittilsen et al. [66] | Prospective | Randomized Controlled Trial | Schizophrenia | Not mentioned |
| Banitalebi et al. [67] | Prospective | Randomized Controlled Trial | Type 2 Diabetes | Insufficiently Active |
| Beetham et al. [68] | Prospective | Randomized Controlled Trial | Chronic Kidney Disease | Not mentioned |
| Benda et al. [69] | Prospective | Randomized Controlled Trial | Heart Failure | Not mentioned |
| Benham et al. [70] | Prospective | Randomized Controlled Trial | Polycystic Ovary Syndrome | Insufficiently Active |
| Berger et al. [71] | Prospective | Randomized Controlled Trial | -- | Insufficiently Active |
| Billany et al. [72] | Prospective | Randomized Controlled Trial | Kidney Failure | Not mentioned |
| Bjorke et al. [73] | Prospective | Randomized Controlled Trial | Cancer | Not mentioned |
| Briggs et al. [74] | Prospective | Randomized Controlled Trial | HIV | Insufficiently Active |
| Brobakken et al. [75] | Prospective | Randomized Controlled Trial | Schizophrenia | Not mentioned |
| Cano-Montoya et al. [76] | Prospective | Quasi-Experimental Trial | Type 2 Diabetes/Hypertension | Insufficiently Active |
| Cerini et al. [77] | Prospective | Randomized Controlled Trial | Chronic Low Back Pain | Not mentioned |
| Cheema et al. [78] | Prospective | Randomized Controlled Trial | Obesity | Insufficiently Active |
| Ciolac et al. [79] | Prospective | Randomized Controlled Trial | -- | Insufficiently Active |
| Coletta et al. [80] | Prospective | Randomized Controlled Trial | Breast Cancer | Not mentioned |
| Connolly et al. [81] | Prospective | Randomized Controlled Trial | Arterial Hypertension | Insufficiently Active |
| Conraads et al. [82] | Prospective | Randomized Controlled Trial | Coronary Artery Disease | Not mentioned |
| Cooke et al. [83] | Prospective | Randomized Controlled Trial | -- | Insufficiently Active |
| Cooper et al. [84] | Prospective | Randomized Controlled Trial | -- | Insufficiently Active |
| Currie et al. [85] | Prospective | Randomized Controlled Trial | Coronary Artery Disease | Insufficiently Active |
| Currie et al. [86] | Prospective | Randomized Controlled Trial | Coronary Artery Disease | Not mentioned |
| D’Amuri et al. [87] | Prospective | Randomized Controlled Trial | -- | Insufficiently Active |
| Damme et al. [88] | Prospective | Randomized Controlled Trial | Attenuated Psychosis Syndrome | Insufficiently Active |
| Deraas et al. [89] | Prospective | Observational Trial | -- | Insufficiently Active |
| Devin et al. [90] | Prospective | Randomized Controlled Trial | Colorectal Cancer | Not mentioned |
| Devin et al. [91] | Prospective | Randomized Controlled Trial | Colorectal Cancer | Not mentioned |
| Dissing et al. [92] | Prospective | Observational Trial | Type 2 Diabetes | Not mentioned |
| Dolan et al. [93] | Prospective | Randomized Controlled Trial | Breast Cancer | Not mentioned |
| Dowd et al. [94] | Prospective | Randomized Controlled Trial | Coeliac Disease | Insufficiently Active |
| Egegaard et al. [95] | Prospective | Randomized Controlled Trial | Non-Small Cell Lung Cancer | Not mentioned |
| Eichner et al. [96] | Prospective | Randomized Controlled Trial | Prediabetes | Insufficiently Active |
| Ellingsen et al. [97] | Prospective | Randomized Controlled Trial | Heart Failure | Insufficiently Active |
| Elmer et al. [98] | Prospective | Matched Group Trial | -- | Insufficiently Active |
| Emtner et al. [99] | Prospective | Observational Trial | Asthma | Insufficiently Active |
| Emtner et al. [100] | Prospective | Randomized Controlled Trial | Asthma | Insufficiently Active |
| Flaherty et al. [101] | Prospective | Randomized Controlled Trial | -- | Insufficiently Active |
| Flemmen et al. [102] | Prospective | Randomized Controlled Trial | Substance Use Disorder | Insufficiently Active |
| Foster et al. [103] | Prospective | Randomized Controlled Trial | -- | Insufficiently Active |
| Francois et al. [104] | Prospective | Randomized Controlled Trial | Type 2 Diabetes | Not mentioned |
| Freese et al. [105] | Prospective | Randomized Controlled Trial | Metabolic Syndrome | Not mentioned |
| Freitag et al. [106] | Prospective | Case Series | Cancer | Insufficiently Active |
| Freyssin et al. [107] | Prospective | Randomized Controlled Trial | Heart Failure | Not mentioned |
| Gauthier et al. [108] | Prospective | Randomized Controlled Trial | Spinal Cord Injury | Varying Levels of Activity |
| Gilbertson et al. [109] | Prospective | Randomized Controlled Trial | Prediabetes | Insufficiently Active |
| Gildea et al. [110] | Prospective | Randomized Controlled Trial | Type 2 Diabetes | Insufficiently Active |
| Gillen et al. [111] | Prospective | Observational Trial | Obesity | Insufficiently Active |
| Gillen et al. [112] | Prospective | Randomized Controlled Trial | -- | Insufficiently Active |
| Gloeckl et al. [113] | Prospective | Randomized Controlled Trial | COPD | Insufficiently Active |
| Golightly et al. [114] | Prospective | Observational Trial | Knee Osteoarthritis | Insufficiently Active |
| Gorostegi-Anduaga et al. [115] | Prospective | Randomized Controlled Trial | Obesity/Hypertension | Insufficiently Active |
| Grace et al. [116] | Prospective | Non-Randomized Trial | -- | Insufficiently Active |
| Gremeaux et al. [117] | Retrospective | Observational Trial | Obesity | Not mentioned |
| Guillamo et al. [118] | Prospective | Non-Randomized Trial | Multiple Sclerosis | Not mentioned |
| Haines et al. [119] | Prospective | Randomized Controlled Trial | Hyperglycemia | Not mentioned |
| Hatle et al. [120] | Prospective | Randomized Controlled Trial | -- | Insufficiently Active |
| Hearon et al. [121] | Prospective | Randomized Controlled Trial | Heart Failure | Not mentioned |
| Heggelund et al. [122] | Prospective | Non-Randomized Trial | Schizophrenia | Not mentioned |
| Heje et al. [123] | Prospective | Randomized Controlled Trial | Muscular Dystrophy | Insufficiently Active |
| Hesketh et al. [124] | Prospective | Non-randomized trial | Cardiovascular Disease | Not mentioned |
| Hettchen et al. [125] | Prospective | Randomized Controlled Trial | Osteopenia/Osteoporosis | Insufficiently Active |
| Heydari et al. [126] | Prospective | Randomized Controlled Trial | -- | Insufficiently Active |
| Higgins et al. [127] | Prospective | Randomized Controlled Trial | -- | Insufficiently Active |
| Hindso et al. [128] | Prospective | Observational Trial | -- | Insufficiently Active |
| Howden et al. [129] | Prospective | Randomized Controlled Trial | -- | Insufficiently Active |
| Humphreys et al. [130] | Prospective | Observational Trial | Multiple Sclerosis | Not mentioned |
| Hwang et al. [131] | Prospective | Randomized Controlled Trial | Lung Cancer | Not mentioned |
| Hwang et al. [132] | Prospective | Randomized Controlled Trial | -- | Insufficiently Active |
| Iellamo et al. [133] | Prospective | Randomized Controlled Trial | Heart Failure | Not mentioned |
| Ivanova et al. [134] | Prospective | Randomized Controlled Trial | Prediabetes | Insufficiently Active |
| Izadi et al. [135] | Prospective | Randomized Controlled Trial | Hypertension | Insufficiently Active |
| Jabbour et al. [136] | Prospective | Observational Trial | -- | Insufficiently Active |
| Jabbour et al. [137] | Prospective | Observational Trial | -- | Insufficiently Active |
| Jakobsen et al. [138] | Prospective | Randomized Controlled Trial | -- | Insufficiently Active |
| Jung et al. [139] | Prospective | Randomized Controlled Trial | Prediabetes | Insufficiently Active |
| Jung et al. [26] | Prospective | Randomized Controlled Trial | Prediabetes | Insufficiently Active |
| Kang et al. [140] | Prospective | Randomized Controlled Trial | Prostate Cancer | Insufficiently Active |
| Karlsen et al. [141] | Prospective | Randomized Controlled Trial | Coronary Artery Disease | Not mentioned |
| Karstoft et al. [142] | Prospective | Randomized Controlled Trial | Type 2 Diabetes | Insufficiently Active |
| Kaur et al. [143] | Prospective | Cohort Multiple Randomized Controlled Trial | HIV | Insufficiently Active |
| Keating et al. [144] | Prospective | Randomized Controlled Trial | -- | Insufficiently Active |
| Keating et al. [145] | Prospective | Observational Trial | -- | Insufficiently Active |
| Kemmler et al. [146] | Prospective | Randomized Controlled Trial | -- | Insufficiently Active |
| Keogh et al. [147] | Prospective | Randomized Controlled Trial | Osteoarthritis | Not mentioned |
| Keteyian et al. [148] | Prospective | Randomized Controlled Trial | Myocardial Infarction | Not mentioned |
| Keytsman et al. [149] | Prospective | Randomized Controlled Trial | Multiple Sclerosis | Not mentioned |
| Kiel et al. [150] | Prospective | Randomized Controlled Trial | Polycystic Ovary Syndrome | Insufficiently Active |
| Klonizakis et al. [151] | Prospective | Randomized Controlled Trial | -- | Insufficiently Active |
| Knowles et al. [152] | Prospective | Observational Trial | -- | Insufficiently Active |
| Kong et al. [153] | Prospective | Randomized Controlled Trial | Obesity | Insufficiently Active |
| Lanzi et al. [154] | Prospective | Randomized Controlled Trial | Obesity | Not mentioned |
| Lee et al. [155] | Prospective | Randomized Controlled Trial | Type 1 Diabetes | Insufficiently Active |
| Lee et al. [156] | Prospective | Randomized Controlled Trial | Breast Cancer | Insufficiently Active |
| Locke et al. [157] | Prospective | Randomized Controlled Trial | Prediabetes | Insufficiently Active |
| Lunt et al. [25] | Prospective | Randomized Controlled Trial | -- | Insufficiently Active |
| Lyall et al. [158] | Prospective | Randomized Controlled Trial | -- | Insufficiently Active |
| MacDonald et al. [159] | Prospective | Observational Trial | Chronic Lymphocytic Leukemia | Not mentioned |
| MacLean et al. [160] | Prospective | Observational Trial | Non-alcoholic Fatty Liver Disease | Not mentioned |
| Madsen et al. [161] | Prospective | Randomized Controlled Trial | Type 2 Diabetes | Insufficiently Active |
| Madssen et al. [162] | Prospective | Randomized Controlled Trial | Coronary Artery Disease | Not mentioned |
| Madssen et al. [163] | Prospective | Randomized Controlled Trial | Cardiovascular Disease | Not mentioned |
| Martin et al. [164] | Prospective | Observational Trial | Abdominal Condition | Not mentioned |
| Martins et al. [165] | Prospective | Randomized Controlled Trial | Obesity | Insufficiently Active |
| Matsuo et al. [166] | Prospective | Randomized Controlled Trial | -- | Insufficiently Active |
| Mendelson et al. [167] | Prospective | Randomized Controlled Trial | Obesity | Insufficiently Active |
| Metcalfe et al. [168] | Prospective | Randomized Controlled Trial | -- | Insufficiently Active |
| Metcalfe et al. [169] | Prospective | Randomized Controlled Trial | -- | Insufficiently Active |
| Metcalfe et al. [170] | Prospective | Randomized Feasibility Trial | -- | Insufficiently Active |
| Midtgaard et al. [171] | Prospective | Randomized Controlled Trial | Cancer | Not mentioned |
| Mijwel et al. [172] | Prospective | Randomized Controlled Trial | Breast Cancer | Not mentioned |
| Moholdt et al. [173] | Prospective | Randomized Controlled Trial | Coronary Artery Disease | Not mentioned |
| Moholdt et al. [174] | Prospective | Randomized Controlled Trial | Coronary Artery Disease | Not mentioned |
| Munk et al. [175] | Prospective | Randomized Controlled Trial | Coronary Artery Disease | Not mentioned |
| Nikseresht et al. [176] | Prospective | Randomized Controlled Trial | Obesity | Insufficiently Active |
| Nilsson et al. [177] | Prospective | Randomized Controlled Trial | Kidney Failure | Not mentioned |
| Northey et al. [178] | Prospective | Randomized Controlled Trial | Breast Cancer | Insufficiently Active |
| Nybo et al. [179] | Prospective | Non-Randomized Trial | -- | Insufficiently Active |
| Nytroen et al. [180] | Prospective | Randomized Controlled Trial | Cardiovascular Disease | Not mentioned |
| Nytroen et al. [181] | Prospective | Randomized Controlled Trial | Cardiovascular Disease | Not mentioned |
| Olsen et al. [182] | Prospective | Randomized Controlled Trial | Coronary Artery Disease | Not mentioned |
| Papadopoulos et al. [183] | Prospective | Randomized Controlled Trial | Prostate Cancer | Insufficiently Active |
| Pattyn et al. [184] | Prospective | Randomized Controlled Trial | Coronary Artery Disease | Not mentioned |
| Pedersen et al. [185] | Prospective | Randomized Controlled Trial | Coronary Artery Disease | Insufficiently Active |
| Phillips et al. [186] | Prospective | Multi-Stage Clinical Trial | Prediabetes | Insufficiently Active |
| Piraux et al. [187] | Prospective | Randomized Controlled Trial | Rectal Cancer | Insufficiently Active |
| Poon et al. [188] | Prospective | Randomized Controlled Trial | Obesity | Insufficiently Active |
| Poon et al. [189] | Prospective | Randomized Controlled Trial | Obesity | Insufficiently Active |
| Rakobowchuk et al. [190] | Prospective | Non-Randomized Trial | -- | Insufficiently Active |
| Reljic et al. [191] | Prospective | Randomized Controlled Trial | -- | Insufficiently Active |
| Reljic et al. [192] | Prospective | Randomized Controlled Trial | Cancer | Not mentioned |
| Robinson et al. [193] | Prospective | Randomized Controlled Trial | Prediabetes | Insufficiently Active |
| Rolid et al. [194] | Prospective | Randomized Controlled Trial | Cardiovascular Disease | Not mentioned |
| Romain et al. [195] | Prospective | Randomized Controlled Trial | Psychotic Disorder | Insufficiently Active |
| Rowan et al. [196] | Prospective | Randomized Controlled Trial | Prediabetes | Insufficiently Active |
| Roxburgh et al. [197] | Prospective | Randomized Controlled Trial | -- | Insufficiently Active |
| Roy et al. [198] | Prospective | Randomized Controlled Trial | Obesity | Not mentioned |
| Ruffino et al. [199] | Prospective | Randomized Controlled Trial | Type 2 Diabetes | Varying Levels of Activity |
| Rustad et al. [200] | Prospective | Randomized Controlled Trial | Cardiovascular Disease | Not mentioned |
| Saanijoki et al. [201] | Prospective | Randomized Controlled Trial | -- | Insufficiently Active |
| Safiyari-Hafizi et al. [202] | Prospective | Randomized Controlled Trial | Heart Failure | Insufficiently Active |
| Sargeant et al. [203] | Prospective | Randomized Controlled Trial | Non-alcoholic Fatty Liver Disease | Insufficiently Active |
| Sawyer et al. [204] | Prospective | Randomized Controlled Trial | Obesity | Not mentioned |
| Schmitt et al. [205] | Prospective | Randomized Controlled Trial | Cancer | Not mentioned |
| Schulz et al. [206] | Prospective | Randomized Controlled Trial | Breast Cancer | Not mentioned |
| Scott et al. [207] | Prospective | Observational Trial | Type 1 Diabetes | Not mentioned |
| Shenouda et al. [208] | Prospective | Randomized Controlled Trial | -- | Insufficiently Active |
| Shepherd et al. [209] | Prospective | Randomized Controlled Trial | -- | Insufficiently Active |
| Sim et al. [210] | Prospective | Randomized Controlled Trial | -- | Insufficiently Active |
| Simonsen et al. [211] | Prospective | Controlled Abode-Based Feasibility Study | Cancer | Not mentioned |
| Smith-Ryan et al. [212] | Prospective | Randomized Controlled Trial | Obesity | Insufficiently Active |
| Smith-Ryan et al. [213] | Prospective | Observational Trial | Cardiovascular Disease Risk | Not mentioned |
| Sogaard et al. [214] | Prospective | Observational Trial | -- | Insufficiently Active |
| Stavrinou et al. [215] | Prospective | Randomized Controlled Trial | -- | Insufficiently Active |
| Sveaas et al. [216] | Retrospective | Randomized Controlled Trial | Spondyloarthritis | Insufficiently Active |
| Taylor et al. [217] | Prospective | Randomized Controlled Trial | Coronary Artery Disease | Not mentioned |
| Terada et al. [218] | Prospective | Randomized Controlled Trial | Type 2 Diabetes | Insufficiently Active |
| Tew et al. [219] | Prospective | Randomized Controlled Trial | Crohn's disease | Insufficiently active |
| Tjonna et al. [220] | Prospective | Randomized Controlled Trial | Metabolic Syndrome | Not mentioned |
| Toennesen et al. [221] | Prospective | Randomized Controlled Trial | Asthma | Not mentioned |
| Tong et al. [222] | Prospective | Randomized Controlled Trial | Obesity | Insufficiently Active |
| Tschentscher et al. [223] | Prospective | Randomized Controlled Trial | Coronary Artery Disease | Not mentioned |
| Tsirigkakis et al. [224] | Prospective | Randomized Controlled Trial | Obesity | Insufficiently Active |
| Turri-Silva et al. [225] | Prospective | Randomized Controlled Trial | Heart Failure | Insufficiently Active |
| Valent et al. [226] | Prospective | Observational Trial | Spinal Cord Injury | Insufficiently Active |
| Vella et al. [227] | Prospective | Randomized Controlled Trial | Obesity | Insufficiently Active |
| Verbrugghe et al. [228] | Prospective | Non-Randomized Trial | Chronic Low Back Pain | Not mentioned |
| Verbrugghe et al. [229] | Prospective | Randomized Controlled Trial | Chronic Low Back Pain | Not mentioned |
| Vestergaard et al. [230] | Prospective | Observational Trial | Spinal Cord Injury | Not mentioned |
| Vidal-Almela et al. [231] | Prospective | Observational Trial | Cardiovascular Disease | Not mentioned |
| Way et al. [232] | Retrospective | Exploratory Retrospective Analysis | Cardiovascular Disease | Not mentioned |
| Weng et al. [233] | Prospective | Randomized Controlled Trial | -- | Insufficiently Active |
| Willoughby et al. [234] | Prospective | Randomized Controlled Trial | -- | Insufficiently Active |
| Wilson et al. [235] | Prospective | Randomized Controlled Trial | Type 2 Diabetes | Not mentioned |
| Winding et al. [236] | Prospective | Randomized Controlled Trial | Type 2 Diabetes | Not mentioned |
| Wormgoor et al. [237] | Prospective | Randomized Controlled Trial | Type 2 Diabetes | Not mentioned |
| Zhang et al. [238] | Prospective | Randomized Controlled Trial | Obesity | Insufficiently Active |
| Zisko et al. [239] | Prospective | Randomized Controlled Trial | -- | Insufficiently Active |
