# Supplementary material for: Rates of compliance and adherence to high-intensity interval training: a systematic review and Meta-analyses
Source: Int J Behav Nutr Phys Act. 2023 Nov 21;20:134. doi: 10.1186/s12966-023-01535-w (PMC10664287; doi:10.1186/s12966-023-01535-w)
Supplement: Supplementary file 6 — Additional File 6. Table including group allocation information for each included study, such as total sample size, number of arms, allocation ratio, and types of groups included (HIIT, MICT, Control, Others). [file 12966_2023_1535_MOESM6_ESM.docx]

**Additional File 6.** Group Allocation Information

| **Study Reference** | **Total Sample Size** | | **Number of Arms** | **Allocation Ratio** | **HIIT Group** | **MICT Group** | **Control Group** | **Other Group** |
| --- | --- | --- | --- | --- | --- | --- | --- | --- |
| Aamot et al. [54] | | 90 | 3 | 1:1:1 | Yes | No | Yes | Group-Based Circuit Training |
| Adams et al. [55] | | 63 | 2 | 1:1 | Yes | No | Yes | No |
| Allen et al. [56] | | 55 | 3 | 1:1:1 | Yes | No | Yes | Prolonged Intermittent Sprint Training |
| Allen et al. [57] | | 32 | 3 | 1:1:1 | Yes | Yes | Yes | No |
| Allison et al. [58] | | 23 | 2 | -- | Yes | No | No | 60-Second Stair Climbing |
| Alvarez et al. [59] | | 28 | 2 | 1:1 | Yes | No | Yes | No |
| Arad et al. [60] | | 28 | 2 | 1:1 | Yes | No | Yes | No |
| Archila et al. [61] | | 22 | 2 | 1:1 | Yes | No | Yes | No |
| Astorino et al. [62] | | 30 | 3 | 2:2:1 | Yes | No | Yes | Moderate-Intensity Interval Training |
| Atan et al. [63] | | 60 | 3 | 1:1:1 | Yes | Yes | Yes | No |
| Avila-Gandi­a et al. [64] | | 60 | 3 | 1:1:1 | Yes | No | Yes | Low-Moderate Intensity Training |
| Baekkerud et al. [65] | | 30 | 3 | 1:1:1 | Yes | Yes | No | 1-HIIT |
| Bang-Kittilsen et al. [66] | | 82 | 2 | 1:1 | Yes | No | No | Active Video Gaming |
| Banitalebi et al. [67] | | 52 | 3 | 1:1:1 | Yes | No | Yes | Aerobic + Resistance Training |
| Beetham et al. [68] | | 21 | 2 | 1:1 | Yes | Yes | No | No |
| Benda et al. [69] | | 33 | 3 | 1:2:2 | Yes | Yes | Yes | No |
| Benham et al. [70] | | 47 | 3 | 1:1:1 | Yes | Yes | Yes | No |
| Berger et al. [71] | | 23 | 3 | 1:1:1 | Yes | Yes | Yes | No |
| Billany et al. [72] | | 24 | 3 | 1:1:1 | Yes | Yes | No | 4x4minute HIIT |
| Bjorke et al. [73] | | 255 | 2 | 1:1 | Yes | Yes | No | No |
| Briggs et al. [74] | | 26 | 2 | 1:1 | Yes | No | No | High Intensity Continuous Training |
| Brobakken et al. [75] | | 48 | 2 | 1:1 | Yes | No | Yes | No |
| Cano-Montoya et al. [76] | | 23 | 1 | -- | Yes | No | No | No |
| Cerini et al. [77] | | 30 | 2 | 1:1 | Yes | Yes | No | No |
| Cheema et al. [78] | | 12 | 2 | 1:1 | Yes | Yes | No | No |
| Ciolac et al. [79] | | 44 | 3 | 1:1:1 | Yes | Yes | Yes | No |
| Coletta et al. [80] | | 44 | 3 | 1:1:1 | Yes | Yes | Yes | No |
| Connolly et al. [81] | | 62 | 3 | 1:1:1 | Yes | No | Yes | Low-Intensity Continuous Swimming |
| Conraads et al. [82] | | 200 | 2 | 1:1 | Yes | Yes | No | No |
| Cooke et al. [83] | | 45 | 3 | 1:1:1 | Yes | No | Yes | SIT + Weekly Fasting Diet |
| Cooper et al. [84] | | 62 | 4 | 1:1:1:1 | Yes | Yes | Yes | Passive Rest Sprint Interval Training |
| Currie et al. [85] | | 22 | 2 | 1:1 | Yes | Yes | No | No |
| Currie et al. [86] | | 19 | 2 | 1:1 | Yes | Yes | No | No |
| D’Amuri et al. [87] | | 44 | 2 | 1:1 | Yes | Yes | No | No |
| Damme et al. [88] | | 37 | 2 | 1:1 | Yes | No | Yes | No |
| Deraas et al. [89] | | 16 | 1 | -- | Yes | No | No | No |
| Devin et al. [90] | | 47 | 2 | 2:1 | Yes | Yes | No | No |
| Devin et al. [91] | | 57 | 3 | 1:1:1 | Yes | Yes | No | Tapered HIIT |
| Dissing et al. [92] | | 10 | 1 | -- | Yes | No | No | No |
| Dolan et al. [93] | | 36 | 3 | 1:1:1 | Yes | Yes | Yes | No |
| Dowd et al. [94] | | 41 | 2 | 1:1 | Yes | No | Yes | No |
| Egegaard et al. [95] | | 15 | 2 | 1:1 | Yes | No | Yes | No |
| Eichner et al. [96] | | 31 | 2 | 1:1 | Yes | Yes | No | No |
| Ellingsen et al. [97] | | 247 | 3 | 1:1:1 | Yes | Yes | Yes | No |
| Elmer et al. [98] | | 14 | 2 | 1:1 | Yes | Yes | No | No |
| Emtner et al. [99] | | 26 | 1 | -- | Yes | No | No | No |
| Emtner et al. [100] | | 32 | 2 | 1:1 | Yes | No | No | Water Training |
| Flaherty et al. [101] | | 28 | 2 | 1:1 | Yes | No | Yes | No |
| Flemmen et al. [102] | | 24 | 2 | 1:1 | Yes | No | Yes | No |
| Foster et al. [103] | | 65 | 3 | 1:1:1 | Yes | Yes | No | Meyer HIIT |
| Francois et al. [104] | | 53 | 3 | 1:1:1 | Yes | No | Yes | Macronutrient Control |
| Freese et al. [105] | | 76 | 2 | 1:1 | Yes | No | Yes | No |
| Freitag et al. [106] | | 1 | 1 | -- | Yes | No | No | No |
| Freyssin et al. [107] | | 26 | 2 | 1:1 | Yes | Yes | No | No |
| Gauthier et al. [108] | | 11 | 2 | 1:1 | Yes | Yes | No | No |
| Gilbertson et al. [109] | | 29 | 2 | 1:1 | Yes | Yes | No | No |
| Gildea et al. [110] | | 35 | 3 | 1:1:1 | Yes | Yes | Yes | No |
| Gillen et al. [111] | | 14 | 1 | -- | Yes | No | No | No |
| Gillen et al. [112] | | 27 | 3 | 1:1:1 | Yes | Yes | Yes | No |
| Gloeckl et al. [113] | | 71 | 2 | 1:1 | Yes | Yes | No | No |
| Golightly et al. [114] | | 30 | 1 | -- | Yes | No | No | No |
| Gorostegi-Anduaga et al. [115] | | 175 | 4 | 1:1:1:1 | Yes | Yes | Yes | Low-Volume HIIT |
| Grace et al. [116] | | 44 | 2 | -- | Yes | No | Yes | No |
| Gremeaux et al. [117] | | 69 | 1 | -- | Yes | No | No | No |
| Guillamo et al. [118] | | 29 | 3 | -- | Yes | No | Yes | Home-Based Exercise |
| Haines et al. [119] | | 40 | 2 | 1:1 | Yes | No | Yes | No |
| Hatle et al. [120] | | 21 | 2 | 1:1 | Yes | No | No | Moderate-Frequency HIIT |
| Hearon et al. [121] | | 80 | 2 | 1:1 | Yes | No | Yes | No |
| Heggelund et al. [122] | | 25 | 2 | 2:1 | Yes | No | Yes | No |
| Heje et al. [123] | | 10 | 2 | 1:1 | Yes | No | Yes | No |
| Hesketh et al. [124] | | 154 | 2 | -- | Yes | Yes | No | No |
| Hettchen et al. [125] | | 54 | 2 | 1:1 | Yes | No | Yes | No |
| Heydari et al. [126] | | 46 | 2 | 1:1 | Yes | No | Yes | No |
| Higgins et al. [127] | | 60 | 2 | 1:1 | Yes | Yes | No | No |
| Hindso et al. [128] | | 10 | 1 | -- | Yes | No | No | No |
| Howden et al. [129] | | 61 | 2 | 1:2:1 | Yes | No | Yes | No |
| Humphreys et al. [130] | | 11 | 1 | -- | Yes | No | No | No |
| Hwang et al. [131] | | 24 | 2 | 1:1 | Yes | No | Yes | No |
| Hwang et al. [132] | | 51 | 3 | 1:1:1 | Yes | Yes | Yes | No |
| Iellamo et al. [133] | | 20 | 2 | 1:1 | Yes | Yes | No | No |
| Ivanova et al. [134] | | 32 | 2 | 1:1 | Yes | Yes | No | No |
| Izadi et al. [135] | | 44 | 2 | 1:1 | Yes | No | Yes | No |
| Jabbour et al. [136] | | 30 | 1 | -- | Yes | No | No | No |
| Jabbour et al. [137] | | 30 | 2 | 1:1 | Yes | No | No | Older Adults |
| Jakobsen et al. [138] | | 43 | 4 | 1:1:1:1 | Yes | Yes | Yes | Soccer |
| Jung et al. [139] | | 32 | 2 | 1:1 | Yes | Yes | No | No |
| Jung et al. [26] | | 99 | 2 | 1:1 | Yes | Yes | No | No |
| Kang et al. [140] | | 52 | 2 | 1:1 | Yes | No | Yes | No |
| Karlsen et al. [141] | | 21 | 2 | 1:1 | Yes | No | Yes | No |
| Karstoft et al. [142] | | 27 | 3 | 1:1:1 | Yes | Yes | Yes | No |
| Kaur et al. [143] | | 32 | 1 | -- | Yes | No | Yes | Refused Intervention |
| Keating et al. [144] | | 38 | 3 | 1:1:1 | Yes | Yes | Yes | No |
| Keating et al. [145] | | 10 | 1 | -- | Yes | No | No | No |
| Kemmler et al. [146] | | 81 | 2 | 1:1 | Yes | Yes | No | No |
| Keogh et al. [147] | | 17 | 2 | 1:1 | Yes | Yes | No | No |
| Keteyian et al. [148] | | 39 | 2 | 1:1 | Yes | Yes | No | No |
| Keytsman et al. [149] | | 55 | 2 | 1:1 | Yes | No | Yes | No |
| Kiel et al. [150] | | 64 | 3 | 1:1:1 | Yes | No | Yes | High-Volume HIIT |
| Klonizakis et al. [151] | | 22 | 2 | 1:1 | Yes | Yes | No | No |
| Knowles et al. [152] | | 44 | 2 | 1:1 | Yes | No | Yes | No |
| Kong et al. [153] | | 31 | 2 | 1:1 | Yes | Yes | No | No |
| Lanzi et al. [154] | | 20 | 2 | 1:1 | Yes | Yes | No | No |
| Lee et al. [155] | | 30 | 2 | 1:1 | Yes | No | Yes | No |
| Lee et al. [156] | | 30 | 2 | 1:1 | Yes | No | Yes | No |
| Locke et al. [157] | | 32 | 2 | 1:1 | Yes | Yes | No | No |
| Lunt et al. [25] | | 49 | 3 | 1:1:1 | Yes | Yes | No | Maximal Volitional Intensity Training |
| Lyall et al. [158] | | 27 | 2 | 1:1 | Yes | No | No | Moderate-Intensity Interval Training |
| MacDonald et al. [159] | | 18 | 2 | 3:2 | Yes | No | Yes | No |
| MacLean et al. [160] | | 12 | 1 | -- | Yes | No | No | No |
| Madsen et al. [161] | | 23 | 2 | 1:1 | Yes | No | Yes | No |
| Madssen et al. [162] | | 41 | 2 | 1:1 | Yes | Yes | No | No |
| Madssen et al. [163] | | 49 | 2 | 1:1 | Yes | No | Yes | No |
| Martin et al. [164] | | 20 | 1 | -- | Yes | No | No | No |
| Martins et al. [165] | | 46 | 3 | 1:1:1 | Yes | Yes | No | Half-Duration HIIT |
| Matsuo et al. [166] | | 42 | 3 | 1:1:1 | Yes | Yes | No | Sprint Interval Training |
| Mendelson et al. [167] | | 60 | 3 | 1:1:1 | Yes | Yes | No | Recovery Modulation HIIT |
| Metcalfe et al. [168] | | 29 | 2 | 1:1 | Yes | No | Yes | No |
| Metcalfe et al. [169] | | 50 | 1 | -- | Yes | No | No | No |
| Metcalfe et al. [170] | | 29 | 2 | 1:1 | Yes | No | Yes | No |
| Midtgaard et al. [171] | | 214 | 2 | 1:1 | Yes | No | Yes | No |
| Mijwel et al. [172] | | 240 | 3 | 1:1:1 | Yes | No | Yes | Resistance Training HIIT |
| Moholdt et al. [173] | | 69 | 2 | 1:1 | Yes | Yes | No | No |
| Moholdt et al. [174] | | 30 | 2 | 1:1 | Yes | Yes | No | No |
| Munk et al. [175] | | 40 | 2 | 1:1 | Yes | No | Yes | No |
| Nikseresht et al. [176] | | 55 | 3 | 1:1:1 | Yes | No | Yes | Non-Linear Resistance Training |
| Nilsson et al. [177] | | 20 | 3 | 1:1:1 | Yes | Yes | Yes | No |
| Northey et al. [178] | | 17 | 3 | 1:1:1 | Yes | Yes | Yes | No |
| Nybo et al. [179] | | 36 | 4 | -- | Yes | Yes | Yes | Strength Training |
| Nytroen et al. [180] | | 52 | 2 | 1:1 | Yes | No | Yes | No |
| Nytroen et al. [181] | | 81 | 2 | 1:1 | Yes | Yes | No | No |
| Olsen et al. [182] | | 70 | 2 | 1:1 | Yes | No | No | Low-Energy Diet |
| Papadopoulos et al. [183] | | 18 | 3 | 1:1:1 | Yes | No | Yes | Resistance Training |
| Pattyn et al. [184] | | 200 | 2 | 1:1 | Yes | Yes | No | No |
| Pedersen et al. [185] | | 70 | 2 | 1:1 | Yes | No | Yes | No |
| Phillips et al. [186] | | 223 | 3 | -- | Yes | No | Yes | 7-by-1 HIIT |
| Piraux et al. [187] | | 18 | 3 | 1:1:1 | Yes | No | Yes | Resistance Training |
| Poon et al. [188] | | 24 | 2 | 1:1 | Yes | Yes | No | No |
| Poon et al. [189] | | 48 | 4 | 1:1:1:1 | Yes | Yes | Yes | Alternating HIIT-MICT |
| Rakobowchuk et al. [190] | | 20 | 2 | 1:1 | Yes | No | No | Moderate-Intensity Interval Training |
| Reljic et al. [191] | | 34 | 3 | 1:1:1 | Yes | Yes | No | 5-by-1 HIIT |
| Reljic et al. [192] | | 27 | 2 | 1:1 | Yes | No | Yes | No |
| Robinson et al. [193] | | 39 | 2 | 1:1 | Yes | Yes | No | No |
| Rolid et al. [194] | | 83 | 2 | 1:1 | Yes | Yes | No | No |
| Romain et al. [195] | | 66 | 2 | 1:1 | Yes | No | Yes | No |
| Rowan et al. [196] | | 21 | 2 | 1:1 | Yes | Yes | No | No |
| Roxburgh et al. [197] | | 29 | 3 | 1:1:1 | Yes | Yes | Yes | No |
| Roy et al. [198] | | 250 | 2 | 1:1 | Yes | No | Yes | No |
| Ruffino et al. [199] | | 21 | 2 | 1:1 | Yes | Yes | No | No |
| Rustad et al. [200] | | 52 | 2 | 1:1 | Yes | No | Yes | No |
| Saanijoki et al. [201] | | 28 | 2 | 1:1 | Yes | Yes | No | No |
| Safiyari-Hafizi et al. [202] | | 40 | 2 | 1:1 | Yes | No | Yes | No |
| Sargeant et al. [203] | | 9 | 1 | -- | Yes | No | Yes | No |
| Sawyer et al. [204] | | 22 | 2 | 1:1 | Yes | Yes | No | No |
| Schmitt et al. [205] | | 26 | 2 | 1:1 | Yes | Yes | No | No |
| Schulz et al. [206] | | 26 | 2 | 1:1 | Yes | No | Yes | No |
| Scott et al. [207] | | 11 | 1 | -- | Yes | No | No | No |
| Shenouda et al. [208] | | 27 | 3 | 1:1:1 | Yes | Yes | Yes | No |
| Shepherd et al. [209] | | 90 | 2 | 1:1 | Yes | Yes | No | No |
| Sim et al. [210] | | 30 | 3 | 1:1:1 | Yes | Yes | Yes | No |
| Simonsen et al. [211] | | 50 | 2 | -- | Yes | No | Yes | No |
| Smith-Ryan et al. [212] | | 32 | 3 | 1:1:1 | Yes | No | Yes | 2-Minute HIIT |
| Smith-Ryan et al. [213] | | 10 | 1 | -- | Yes | No | No | No |
| Sogaard et al. [214] | | 28 | 1 | -- | Yes | No | No | No |
| Stavrinou et al. [215] | | 35 | 3 | 1:1:1 | Yes | No | Yes | Higher-Frequency HIIT |
| Sveaas et al. [216] | | 28 | 2 | 1:1 | Yes | No | Yes | No |
| Taylor et al. [217] | | 93 | 2 | 1:1 | Yes | Yes | No | No |
| Terada et al. [218] | | 15 | 2 | 1:1 | Yes | Yes | No | No |
| Tew et al. [219] | | 36 | 3 | 1:1:1 | Yes | Yes | Yes | No |
| Tjonna et al. [220] | | 32 | 3 | 1:1:1 | Yes | Yes | Yes | No |
| Toennesen et al. [221] | | 36 | 1 | -- | Yes | No | No | No |
| Tong et al. [222] | | 54 | 3 | 1:1:1 | Yes | No | Yes | Sprint Interval Training |
| Tschentscher et al. [223] | | 66 | 3 | 1:1:1 | Yes | Yes | No | Pyramid Training |
| Tsirigkakis et al. [224] | | 20 | 3 | 1:1 | Yes | No | No | 1-Minute HIIT |
| Turri-Silva et al. [225] | | 27 | 3 | 1:1:1 | Yes | No | Yes | Circuit-Resistance Training |
| Valent et al. [226] | | 22 | 1 | -- | Yes | No | No | No |
| Vella et al. [227] | | 19 | 2 | 1:1 | Yes | Yes | No | No |
| Verbrugghe et al. [228] | | 20 | 2 | 1:1 | Yes | No | Yes | No |
| Verbrugghe et al. [229] | | 38 | 2 | 1:1 | Yes | Yes | No | No |
| Vestergaard et al. [230] | | 8 | 1 | -- | Yes | No | No | No |
| Vidal-Almela et al. [231] | | 140 | 1 | -- | Yes | No | No | No |
| Way et al. [232] | | 151 | 1 | -- | Yes | No | No | No |
| Weng et al. [233] | | 30 | 3 | 1:1:1 | Yes | Yes | Yes | No |
| Willoughby et al. [234] | | 40 | 2 | 1:1 | Yes | No | No | Middle-Aged Adults |
| Wilson et al. [235] | | 16 | 2 | 2:1 | Yes | No | Yes | No |
| Winding et al. [236] | | 29 | 3 | 1:1:1 | Yes | Yes | Yes | No |
| Wormgoor et al. [237] | | 23 | 2 | 1:1 | Yes | Yes | No | No |
| Zhang et al. [238] | | 52 | 3 | 1:1:1 | Yes | Yes | Yes | No |
| Zisko et al. [239] | | 30 | 3 | 1:1:1 | Yes | Yes | No | 4x4-Minute HIIT |

*Notes*. HIIT: high-intensity interval training; MICT: moderate-intensity continuous training; SIT: sprint interval training.
