# Supplementary material for: Rates of compliance and adherence to high-intensity interval training: a systematic review and Meta-analyses
Source: Int J Behav Nutr Phys Act. 2023 Nov 21;20:134. doi: 10.1186/s12966-023-01535-w (PMC10664287; doi:10.1186/s12966-023-01535-w)
Supplement: Supplementary file 7 — Additional File 7. Table including intervention characteristics for supervised interventions, such as mean age (SD), percentage of biological sex, intervention settings, number of sessions per week, total number of sessions, description of interventions, and whether interventions included other components (strength training, other exercise, counselling). [file 12966_2023_1535_MOESM7_ESM.docx]

**Additional File 7.** Supervised Intervention Characteristics

| **Study Reference** | **Mean Age (SD)** | | **% Male** | | **Intervention Setting** | **# Supervised Sessions per Week (Total Sessions)** | **FITT Description** | | **Strength Training Component** | **Other Exercise Component** | **Counselling Component** |
| --- | --- | --- | --- | --- | --- | --- | --- | --- | --- | --- | --- |
|  | **HIIT** | **MICT** | **HIIT** | **MICT** |  | | **HIIT** | **MICT** |  | | |
| Aamot et al. [54] | 58 (8) | -- | 86% | -- | Groups (3-7) | 2 (24) | 4x4min; 85-95% HR peak; Treadmill; 4min breaks. | -- | No | No | No |
| Adams et al. [55] | 44 (11.6) | -- | 100% | -- | Individual | 3 (36) | 4x4min; 75-95% VO2 peak; Treadmill; 3min recovery. | -- | No | No | No |
| Allen et al. [56] | 49.2 (6.1) | -- | 35% | -- | Groups (2-6) | 3 (27) | 5-8x 30s; Maximal sprints; Cycle ergometer; 3-5min recovery. | -- | No | No | No |
| Allen et al. [57] | 30.4 (1.5) | 29.2 (1.8) | 67% | 22% | Individual | 1 (12) | 4x30s; 80-90% HRR; Stationary bike; 2.5min recovery. | 30-40min; Moderate intensity; Running. | No | No | No |
| Allison et al. [58] | 26 (11) | -- | 0% | -- | Individual | 3 (18) | 3x20s; Maximal sprints; Stair climbing; 2min recovery. | -- | No | No | No |
| Alvarez et al. [59] | 46 (3) | -- | 0% | -- | Individual | 3 (48) | 8-14x 30s; 90-100% HRR; Indoor running; 2min recovery. | -- | No | No | No |
| Arad et al. [60] | 29 (4) | -- | 0% | -- | Individual | 3 (42) | 4x 30-60s; 75-95% HRR; Cycle ergometer; 3-3.5min recovery. | -- | No | No | Yes |
| Archila et al. [61] | 20 (1) | -- | 32% | -- | Individual | 3 (18) | 5x1min; 80% HR max; Body weight exercises; 60s recovery. | -- | No | No | No |
| Astorino et al. [62] | 22.7 (5.4) | -- | 0% | -- | Individual | 3 (36) | 6-10x 60s; 85-100% HR max; Cycle ergometer; 75s recovery. | -- | No | No | No |
| Atan et al. [63] | 46.6 (9.4) | 47.4 (8) | 0% | 0% | Individual | 5 (30) | 4x4min; 80-95% HR peak; Cycle ergometer; 3min recovery. | 45min; 65-70% HR peak; Cycle ergometer. | Yes | Yes | No |
| Avila-Gandi­a et al. [64] | 54.2 (7.8) | -- | 43.2% | -- | Individual | 3 (84) | Intervals; 80-90% HR max; Whole-Body Exercises. | -- | Yes | No | No |
| Baekkerud et al. [65] | 39 (10) | 41 (10) | 41.7% | 44.4% | Individual | 3 (18) | 4x4min; 85-95% HR max; Treadmill; 3min recovery. | 45min; 70% HR max; Treadmill. | No | No | No |
| Bang-Kittilsen et al. [66] | 36.6 (14.3) | -- | 60.5% | -- | Mixed | 2 (24) | 4x4min; 85-96% HR max; Treadmill; 3min recovery. | -- | No | No | No |
| Banitalebi et al. [67] | 55.4 (5.9) | 54.1 (5.4) | 0% | 0% | Groups (3-5) | 3 (30) | 4x30s; Maximal sprints; Cycle ergometer; 2min recovery. | 20-30min; 50-70% HR max; Treadmill/Cycle ergometer. | No | No | No |
| Beetham et al. [68] | 60.9 (6.3) | 62.8 (10.5) | 66.7% | 80% | Individual | 3 (36) | 4x4min; 80-95% HR peak; Treadmill; 3min recovery. | 40min; 60% HR peak; Treadmill. | No | No | No |
| Benda et al. [69] | 63 (8) | 64 (8) | 75% | 83% | Individual | 2 (24) | 10x60s; 90% Workload max; Cycle ergometer; 2.5min recovery. | 30min; 60-75% Watt max; Cycle ergometer. | No | No | No |
| Benham et al. [70] | 29.1 (4.1) | 29.5 (4.6) | 0% | 0% | Individual | 3 (78) | 10x30s; 90% HRR; Treadmill/Cycle ergometer/Elliptical; 90s recovery. | 40min; 50-60% HRR; Treadmill/Cycle ergometer/Elliptical. | No | No | No |
| Berger et al. [71] | 23 (4) | 24 (5) | 62.5% | 37.5% | Individual | 3-4 (22) | 20x60s; 90% VO2 peak; Cycling; 1min recovery. | 30min; 60% VO2 peak; Cycling. | No | No | No |
| Billany et al. [72] | 41 (14) | 52 (11) | 67% | 88% | Individual | 3 (24) | 2x4-, 2-, 1min; 80-90% VO2 peak; Cycle ergometer; 2 min recovery. | 40 min; 50-60% VO2 peak; Cycle ergometer. | No | No | No |
| Bjorke et al. [73] | 55 (11) | 53 (10) | 0% | 0% | -- | -- | -- | -- | Yes | No | No |
| Briggs et al. [74] | 63.4 (7.1) | -- | 100% | -- | Individual | 3 (36) | 4x4min; 90-95% HR max; Treadmill; 3min recovery. | -- | Yes | No | No |
| Brobakken et al. [75] | 34 | -- | 68% | -- | Individual | 2 (96) | 4x4min; 85-95% HR peak; Treadmill; 3min recovery. | -- | No | No | Yes |
| Cano-Montoya et al. [76] | 62 (11) | -- | 13% | -- | Individual | 2 (24) | 10x60s; 8-10 BORG10 RPE; Stationary bike; 2min recovery. | -- | No | No | No |
| Cerini et al. [77] | 50.3 (10.1) | 50.4 (13.1) | 42.9% | 50% | Individual | 3 (36) | 10x1min; 90% HRR; Cycle ergometer; 60s recovery. | 20 min; 40-59% HRR; Cycle ergometer. | No | No | No |
| Cheema et al. [78] | 43 (19) | 36 (15) | 50% | 33% | Individual | 4 (48) | 15x2min; >75% HR max; Boxing drills; 1min recovery. | 45min; Moderate intensity; Walking. | No | No | No |
| Ciolac et al. [79] | 24.4 (3.8) | 26.6 (4.9) | 0% | 0% | Individual | 3 (48) | 13x60s; 80-90% VO2 max; Treadmill; 2min recovery. | 40min; 60-70% VO2 max; Treadmill. | No | No | No |
| Coletta et al. [80] | 63.7 (6.9) | 64.6 (12.2) | 0% | 0% | Individual | 3 (36) | 4x4min; 90-100% HR peak; Treadmill; 3min recovery. | 41min; 60-70% HR peak; Treadmill. | No | No | Yes |
| Connolly et al. [81] | 44 (5) | -- | 0% | -- | Individual | 3 (45) | 6-10x 30s; All-out effort; Pool swimming; 2min recovery. | -- | No | No | No |
| Conraads et al. [82] | 57 (8.8) | 59.9 (9.2) | 91% | 89% | Individual | 3 (36) | 4x4min; 85-90% VO2 peak; Stationary bike; 3min recovery. | 37min; 65-75% HR peak; Stationary bike. | No | No | Yes |
| Cooke et al. [83] | 32 (8.3) | -- | 9% | -- | Individual | 3 (48) | 4x20s; 150% VO2 peak; Cycle ergometer; 40s recovery. | -- | No | No | No |
| Cooper et al. [84] | 49.1 (5.3) | 51.1 (5.7) | 100% | 100% | Individual | 3 (36) | 4-10x 30s; Maximal sprints; Cycle ergometer; 3min recovery. | 50-60min; 80% HR max; Cycle ergometer. | No | No | No |
| Currie et al. [85] | 62 (11) | 68 (11) | 90.9% | 90.9% | Individual | 2 (24) | 10x60s; 80-104% PPO; Cycle ergometer; 1min recovery. | 30-50 min; 51-65% PPO; Cycle ergometer. | No | No | No |
| Currie et al. [86] | 63 (8) | 66 (8) | 100% | 90% | Individual | 2 (48) | 10x60s; 85-121% PPO; Cycle ergometer; 1min recovery. | 30-50min; 57% PPO; Cycle ergometer. | Yes | No | No |
| D’Amuri et al. [87] | 40 (7) | 37 (9) | 50% | 56% | Individual | 3 (36) | 3-7x3min; 100% VO2 peak; Treadmill; 90s recovery. | 60% VO2 peak; Treadmill. | No | No | Yes |
| Damme et al. [88] | 21.2 (1.7) | -- | 15% | -- | Individual | 2 (24) | 3x60s; 95% VO2 max; Treadmill; 10min recovery. | -- | No | No | No |
| Deraas et al. [89] | 64.5 | -- | 68.8% | -- | Groups | 1 (21) | 4x4min; 90-95% HR max; Walking; 3min recovery. | -- | Yes | No | Yes |
| Devin et al. [90] | 61.4 (11.1) | 61.5 (10.8) | 60% | 47.1% | Individual | 3 (12) | 4x4min; 85-95% HR peak; Cycle ergometer; 3min recovery. | 50min; 50-70% HR peak; Cycle ergometer. | No | No | No |
| Devin et al. [91] | 60.7 (11.7) | 59.8 (11.4) | 72.2% | 47.4% | Individual | 3 (24) | 4x4min; 85-95% HR peak; Cycle ergometer; 3min recovery. | 50min; 70% HR peak; Cycle ergometer. | No | No | No |
| Dissing et al. [92] | 56.1 (1.9) | -- | 90% | -- | Individual | 3 (8) | 10x60s; 90% HR max; Stationary bike; 60s recovery. | -- | No | No | No |
| Dolan et al. [93] | 56.2 (9) | 56.3 (9) | 0% | 0% | Individual | 3 (18) | 4-6x 2-4min; 80-95% VO2 peak; Treadmill; --. | 3.22-4.02km; 55-60% VO2 peak; Treadmill. | No | No | No |
| Dowd et al. [94] | 42 (12.3) | -- | 80% | -- | Individual | 2 (24) | 14x30s; 90% HR max; 2 min recovery. | -- | No | No | Yes |
| Egegaard et al. [95] | 64 (5.8) | -- | 27.5% | -- | Individual | 5 (35) | 5x30s; 80-95% iPPO; Cycle ergometer; 30s recovery. | -- | No | No | No |
| Eichner et al. [96] | 60.4 (7.7) | 61.3 (9) | 21.4% | 23.5% | Individual | 6 (12) | 10x3min; 90% HR peak; Cycle ergometer; 3min recovery. | 60min; 70% HR peak; Cycle ergometer. | No | No | No |
| Ellingsen et al. [97] | 65 | 60 | 82% | 81% | Individual | 3 (36) | 4x4min; 90-95% HR max; Treadmill/Cycle ergometer; 3min recovery. | 47min; 60-70% HR max; Treadmill/Cycle ergometer. | No | No | Yes |
| Elmer et al. [98] | 21.4 (1.1) | 21.8 (2.1) | 100% | 100% | Individual | 3 (24) | 12x1min; 90-110% VO2 max; Treadmill; 1min recovery. | 30min; 70-80% VO2 max; Treadmill. | No | No | No |
| Emtner et al. [99] | 41 | -- | 23.1% | -- | Groups (2-3) | 5 (10) | 5x2min; 80-90% HR max; Pool swimming; 1.5min recovery. | -- | No | No | Yes |
| Emtner et al. [100] | 38 (12) | -- | 64.3% | -- | Individual | 2-5 (26) | 5x2min; 80-100% HR max; Gymnastic exercises; 1.5min recovery. | -- | No | Yes | Yes |
| Flaherty et al. [101] | 34 (11) | -- | 0% | -- | Individual | 3 (18) | 4-18x 1-4min; 85-95% HRR; Cycle ergometer; 1-3min recovery. | -- | No | No | No |
| Flemmen et al. [102] | 33 (11) | -- | 67% | -- | Individual | 3 (24) | 4x4min; 90-95% HR max; Treadmill; 3min recovery. | -- | No | Yes | Yes |
| Foster et al. [103] | 19.9 | 19.6 | -- | -- | Individual | 3 (24) | 8x20s; 170% Watt max; Cycle ergometer; 10s recovery. | 20min; 90% of VT; Cycle ergometer. | No | No | No |
| Francois et al. [104] | 62 (8) | -- | 38.9% | -- | Individual | 3 (36) | 4-10x 60s; 85-90% HR max; Treadmill/Cycle ergometer/Elliptical; 60s recovery. | -- | Yes | No | No |
| Freese et al. [105] | 51.7 (10.4) | -- | 0% | -- | Individual | 3 (18) | 4-8x 30s; Maximal sprints; Cycle ergometer; 4min recovery. | -- | No | No | No |
| Freitag et al. [106] | 52 | -- | 0% | -- | Individual | 2 (8) | 5x3min; 90% Watt max; Cycle ergometer; 2min recovery. | -- | No | No | No |
| Freyssin et al. [107] | 54 (9) | 55 (12) | 50% | 50% | Individual | 6 (48) | 12x30s; 80% Watt max; Cycle ergometer; 60s recovery. | 45min; VT threshold; Cycle ergometer. | No | Yes | Yes |
| Gauthier et al. [108] | 33.9 (3) | 43.2 (18.5) | 66.7% | 100% | Individual | 3 (18) | -- | -- | No | No | Yes |
| Gilbertson et al. [109] | 45.7 (4.4) | 50.8 (4.4) | -- | -- | Individual | 3 (48) | 4-10x 30s; Maximal sprints; Treadmill; 4min recovery. | 30-60min; 45-55% HRR; Treadmill. | No | No | Yes |
| Gildea et al. [110] | 52 (10) | 53 (10) | 67% | 58% | Individual | 3 (36) | 10x60s; 90% HR max; Cycle ergometer; 60s recovery. | 50min; 80-90% Ventilatory Threshold; Cycle ergometer. | No | No | No |
| Gillen et al. [111] | 29.5 (9.5) | -- | 50% | -- | Individual | 3 (18) | 3x20s; Maximal sprints; Cycle ergometer; 2min recovery. | -- | No | No | No |
| Gillen et al. [112] | 27 (7) | 28 (9) | 100% | 100% | Individual | 3 (31) | 3x20s; Maximal sprints; Cycle ergometer; 2min recovery. | 45min; 70% HR max; Cycle ergometer. | No | No | No |
| Gloeckl et al. [113] | 52 (6) | 55 (7) | 49% | 44.4% | Individual | 5-6 (18) | 12-36x 30s; 100% Watt max; Cycle ergometer; 30s recovery. | 10-30min; 60% Watt max; Cycle ergometer. | Yes | No | Yes |
| Golightly et al. [114] | 63 (7) | -- | 34.5% | -- | Individual | 2 (24) | 10x60s; 90% VO2 peak; Treadmill/Cycle ergometer/Elliptical; 60s recovery. | -- | No | No | No |
| Gorostegi-Anduaga et al. [115] | 53.5 (9.1) | 54.7 (7.6) | 72.7% | 66.7% | Individual | 2 (32) | 1. 4x4min; 90% VO2 peak; Treadmill; 3min recovery.  2. 18x30s; 90% VO2 peak; Cycle ergometer; 30s recovery. | 45min; 65% VO2 peak; Treadmill. | No | No | Yes |
| Grace et al. [116] | 62.7 (5.2) | -- | 100% | -- | Groups (4-6) | 1-2 (9) | 6x30s; Maximal sprints; Cycle ergometer; 3min recovery. | -- | No | No | No |
| Gremeaux et al. [117] | 53.3 (9.7) | -- | 58% | -- | Individual | 2-3 (72) | 2 sets of 10-20x 15-30s; 80% Watt max; Cycle ergometer; 15-30s recovery. | -- | Yes | No | Yes |
| Guillamo et al. [118] | 42 (1) | -- | 25% | -- | Individual | 2 (40) | 3x3-5min; 17-18 BORG20 RPE; Stationary bike; 3min recovery. | -- | Yes | Yes | No |
| Haines et al. [119] | 46.4 (6.1) | -- | -- | -- | Individual | 2-3 (15) | 2-8x 5s; Maximal sprints; Cycle ergometer; ~40s-5min recovery. | -- | No | No | No |
| Hatle et al. [120] | 23.7 (2.1) | -- | 41.7% | -- | Individual | 8 (24) | 4x4-min; 90-95% HR max; Treadmill; 3min recovery. | -- | No | No | No |
| Hearon et al. [121] | 50 (6) | -- | 45% | -- | Individual | 2 (104) | 5-8x 30s-120s; 95% HR peak; Cycle ergometer; 2min recovery. | -- | No | Yes | No |
| Heggelund et al. [122] | 30.5 (8.7) | -- | 75% | -- | Individual | 3 (24) | 4x4min; 85-95% HR peak; Treadmill; 3min recovery. | -- | No | No | No |
| Heje et al. [123] | 53 (12) | -- | 100% | -- | Individual | 3 (24) | 2 sets of 5x10s; Maximal sprints; Cycle ergometer; 50s recovery. | -- | No | No | No |
| Hesketh et al. [124] | 49 (10) | 48 (11) | 54% | 61% | -- | -- | -- | -- | -- | -- | -- |
| Hettchen et al. [125] | 53.6 (2) | -- | 0% | -- | Individual | 3 (36) | 30-60s intervals; 80-85% HR max; Dance; 30-60s recovery. | -- | Yes | No | No |
| Heydari et al. [126] | 24.7 (4.8) | -- | 100% | -- | Individual | 3 (36) | 60x8s; 80-90% HR peak; Cycle ergometer; 12s recovery. | -- | No | No | No |
| Higgins et al. [127] | 20.4 (1.5) | 20.4 (1.5) | 0% | 0% | Groups | 3 (18) | 5-7x 30s; Maximal sprints; Cycle ergometer; 4min recovery. | 20-30min; 60-70% HRR; Cycle ergometer. | No | No | No |
| Hindso et al. [128] | 43 (6) | -- | 60% | -- | Individual | 3 (18) | 7x60s; 95-100% VO2 max; Cycle ergometer; 60s recovery. | -- | No | No | No |
| Howden et al. [129] | 53.2 | -- | 45% | -- | Individual | 3-6 (80) | -- | -- | Yes | No | No |
| Humphreys et al. [130] | 38 (7.1) | -- | 9% | -- | Individual | 2 (12) | 6-10x 60s; 80-90% Watt max; Cycle ergometer; 2min recovery. | -- | No | No | No |
| Hwang et al. [131] | 61 (6.3) | -- | 38.5% | -- | Individual | 3 (24) | 2x5min; 80% VO2 peak; Treadmill/Cycle ergometer; 5min recovery. | -- | No | No | No |
| Hwang et al. [132] | 64.8 (1.4) | 65.6 (1.8) | 35.3% | 50% | Individual | 4 (32) | 4x4min; 90% HR peak; All-extremity ergometer; 3min recovery. | 32min; 70% HR peak; All-extremity ergometer. | No | No | No |
| Iellamo et al. [133] | 62.2 (8) | 62.6 (9) | 100% | 100% | Individual | 2-5 (42) | 4x4min; 75-80% HRR; Treadmill; 3min recovery. | 30-45min; 45-60% HRR; Treadmill. | No | No | No |
| Ivanova et al. [134] | 51 (9.9) | 51 (9.9) | 15.6% | 15.6% | Individual | 5 (10) | 4-10x 60s; 90% HR peak; Treadmill/Cycle ergometer/Elliptical; 60s recovery. | 20-50min; 65% HR peak; Treadmill/Cycle ergometer/Elliptical. | No | No | Yes |
| Izadi et al. [135] | 74.6 (3.8) | -- | 53.3% | -- | Individual | 3 (18) | 10x1.5min; 85-90% HRR; Stationary bike; 2min recovery. | -- | No | No | No |
| Jabbour et al. [136] | 38 (30) | -- | 33.3% | -- | Individual | 3 (18) | 6x6s; Maximal sprints; Cycle ergometer; 2min recovery. | -- | No | No | No |
| Jabbour et al. [137] | 26.2 (2.4) | -- | 29.4% | -- | Individual | 3 (18) | 6x6s; Maximal sprints; Cycle ergometer; 2min recovery. | -- | No | No | No |
| Jakobsen et al. [138] | 36.9 (5) | 29 (5.2) | 100% | 100% | -- | 3 (36) | 5x2min; 90% HR max; Running; 60s recovery. | 45min; 80% HR max; Running. | No | No | No |
| Jung et al. [139] | 51 (11) | 51 (10) | 27.7% | 5.8% | Individual | 5 (10) | 4-10x 60s; 90% HR peak; Treadmill/Stationary bike/Elliptical; 60s recovery. | 20-50min; 65% HR peak; Treadmill/Stationary bike/Elliptical. | No | No | No |
| Jung et al. [26] | 51.8 (8.8) | 50 (9.9) | 27.7% | 28.9% | Individual | 3-4 (7) | 4-10x 60s; 80-90% VO2 peak; Treadmill/Stationary bike/Elliptical; 60s recovery. | 20-50min; 45-55% VO2 peak; Treadmill/Stationary bike/Elliptical. | No | No | Yes |
| Kang et al. [140] | 63.9 (7.5) | -- | 100% | -- | Individual | 3 (36) | 5-8x 2min; 85-95% VO2 peak; Treadmill; 2min recovery. | -- | No | No | No |
| Karlsen et al. [141] | 61.1 (7.1) | -- | 75% | -- | Individual | 3 (30) | 4x4min; 85-95% HR peak; Treadmill; 3min recovery. | -- | No | No | No |
| Karstoft et al. [142] | 57.5 (2.4) | 60.8 (2.2) | 58.3% | 66.7% | Individual | 5 (85) | -- | -- | No | No | No |
| Kaur et al. [143] | 54.4 (6.5) | -- | 85% | -- | Groups (4-5) | 3 (36) | 6x30s; 80-85% HR max; Elliptical; 90s recovery. | -- | Yes | No | No |
| Keating et al. [144] | 41.8 (2.7) | 44.1 (1.9) | 23.1% | 18.2% | Individual | 3 (36) | 4-6x 30-60s; 120% VO2 peak; Cycle ergometer; 2-3min recovery. | 30-45min; 50-65% VO2 peak; Cycle ergometer. | No | No | No |
| Keating et al. [145] | 64.2 (6.1) | -- | 30% | -- | Individual | 2 (12) | 4x4min; 85% HR max; Cycle ergometer; 3min recovery. | -- | No | No | No |
| Kemmler et al. [146] | 43.9 (5) | 42.9 (5.1) | 100% | 100% | Individual | 2-4 (49) | 1.5min intervals; 85-97.5% HR max; Running; 1-3min recovery. | 35-90min; 70-82.5% HR max; Running. | No | No | No |
| Keogh et al. [147] | 59.1 (6.7) | 66.1 (8.8) | 33.3% | 12.5% | Individual | 4 (32) | -- | -- | No | No | No |
| Keteyian et al. [148] | 60 (7) | 58 (9) | 73% | 92% | Individual | 3 (30) | 4x4min; 80-90% HRR; Treadmill; 3min recovery. | 30min; 60-80% HRR; Treadmill. | No | No | Yes |
| Keytsman et al. [149] | 41.7 (8.5) | -- | 66.6% | -- | Individual | 3 (72) | 3-5x 60-90s; 90-100% HR max; Cycle ergometer; 1-3min recovery. | -- | No | No | No |
| Kiel et al. [150] | 30.4 (5) | -- | 0% | -- | Individual | 3 (48) | 10x60s; 90-95% HR max; Treadmill; 60s recovery. | -- | No | No | No |
| Klonizakis et al. [151] | 64 (7) | 64 (4) | 0% | 0% | Individual | 3 (6) | 10x60s; 100% Watt max; Cycle ergometer; 60s recovery. | 40min; 65% Watt max; Cycle ergometer. | No | No | No |
| Knowles et al. [152] | 63 (5) | -- | 100% | -- | Groups (4-6) | 1-2 (9) | 6x30sec; Maximal sprints; Cycle ergometer; 3min recovery. | -- | No | No | No |
| Kong et al. [153] | 21.5 (4) | 20.5 (1.9) | 0% | 0% | Individual | 4 (20) | 60x8s; Maximal sprints; Cycle ergometer; 12s recovery. | 40min; 60% VO2 peak; Cycle ergometer. | No | No | No |
| Lanzi et al. [154] | 34.9 (3.4) | 38.1 (2.3) | 100% | 100% | Individual | 4 (8) | 10x60s; 90% HR max; Cycle ergometer; 60s recovery. | 40-50min; Moderate intensity; Cycle ergometer. | No | Yes | Yes |
| Lee et al. [155] | 40.5 (10) | -- | 40% | -- | Individual | 3 (36) | 4x4min; 85-95% HR peak; Treadmill/Cycle ergometer; 3min recovery. | -- | No | No | Yes |
| Lee et al. [156] | 49.1 (7.9) | -- | 0% | -- | Individual | 3 (24) | 7x60s; 90% PPO; Stationary bike; 2min recovery. | -- | No | No | No |
| Locke et al. [157] | 50.9 (10.6) | 51.1 (9.6) | 6.7% | 23.5% | Individual | 5 (10) | 4-10x 60s; 90% HR peak; Treadmill/Stationary bike/Elliptical; 60s recovery. | 20-50min; 65% HR peak; Treadmill/Stationary bike/Elliptical. | No | No | Yes |
| Lunt et al. [25] | 48.2 (5.6) | 46.3 (5.4) | 25% | 29.4% | Groups | 3 (36) | 4x4min; 85-95% HR max; Treadmill; 3min recovery. | 33min; 65-75% HR max; Treadmill. | No | No | No |
| Lyall et al. [158] | 57 (4) | -- | 0% | -- | Individual | 2 (24) | 30s intervals; 120 +/- 10% Gas Exchange Threshold; Cycle Ergometer; 30s recovery. | -- | No | No | No |
| MacDonald et al. [159] | 63.9 (10.8) | -- | 40% | -- | Individual | 3 (36) | 60-90s intervals; 80-90% VO2 reserve; Treadmill; 60-90s recovery. | -- | Yes | No | No |
| MacLean et al. [160] | 45 (8) | -- | 83.3% | -- | Individual | 2 (12) | 5-10x 6s; Maximal sprints; Cycle ergometer; 60s recovery. | -- | No | No | No |
| Madsen et al. [161] | 56 (2) | -- | 30% | -- | Groups (4-5) | 3 (24) | 10x60s; 90% HR max; Cycle ergometer; 60s recovery. | -- | No | Yes | No |
| Madssen et al. [162] | 55.5 | 60.5 | 93.3% | 71.4% | Individual | 3 (36) | 4x4min; 85-95% HR peak; Treadmill; 3min recovery. | 46min; 70% HR max; Treadmill. | No | No | No |
| Madssen et al. [163] | 64.4 | -- | 75% | -- | Individual | 1-4 (8) | 4x4min; 85-95% HR peak; Treadmill; 3min recovery. | -- | No | No | No |
| Martin et al. [164] | 64 (13.9) | -- | 57.1% | -- | Individual | 3 (9) | 40x15s; 100% Watt max; Cycle ergometer; 15s recovery. | -- | No | No | No |
| Martins et al. [165] | 33.9 (7.8) | 33 (9.9) | 40% | 60% | Individual | 3 (36) | 60x8s; 85-90% HR max; Cycle ergometer; 12s recovery. | 250kcal deficit; 70% HR max; Cycle ergometer. | No | No | No |
| Matsuo et al. [166] | 27.2 (6.4) | 25.9 (6) | 100% | 100% | Individual | 5 (40) | 3x3min; 85-95% VO2 max; Cycle ergometer; 2min recovery. | 40min; 60-65% VO2 max; Cycle ergometer. | No | No | No |
| Mendelson et al. [167] | 52 (8) | 51 (11) | 80% | 85% | Individual | 3 (24) | 22x60s; 100% PPO; Cycle ergometer; 60s recovery. | 32-44min; 50% PPO; Cycle ergometer. | No | No | No |
| Metcalfe et al. [168] | 25 (3) | -- | 46.7% | -- | Individual | 3 (18) | 1-2x 10-20s; Maximal sprints; Cycle ergometer; 3.5min recovery. | -- | No | No | No |
| Metcalfe et al. [169] | 34.5 (9) | -- | 54% | -- | Individual | 3 (18) | 1-2x 10-20s; Maximal sprints; Cycle ergometer; 3.5min recovery. | -- | No | No | No |
| Metcalfe et al. [170] | 48 (8) | -- | 50% | -- | Individual | 2 (12) | 2x20s; Maximal sprints; Cycle ergometer; 3min recovery. | -- | No | No | No |
| Midtgaard et al. [171] | 48.2 (10.1) | -- | 16.7% | -- | Groups | 1 (52) | 30s-6min; 90-95% HR max; Cycle ergometer; 1-2min recovery. | -- | Yes | No | Yes |
| Mijwel et al. [172] | 52.7 (10.3) | -- | 0% | -- | Individual | 2 (32) | 3x3min; 16-18 BORG20 RPE; Cycle ergometer; 60s recovery. | -- | Yes | No | No |
| Moholdt et al. [173] | 60.2 (6.9) | 62 (7.6) | 85.7% | 66.7% | Individual | 5 (16-18) | 4x4min; 90% HR max; Cycle ergometer; 3min recovery. | 46min; 70% HR max; Cycle ergometer. | No | Yes | No |
| Moholdt et al. [174] | 61.7 (8) | 63.6 (7.3) | 78.6% | 81.3% | Individual | 3 (72) | -- | -- | No | No | Yes |
| Munk et al. [175] | 57 (14) | -- | 85% | -- | Groups (10) | 3 (72) | 4x4min; 80-90% HR max; Running/Cycling; 3min recovery. | -- | Yes | No | No |
| Nikseresht et al. [176] | 39.6 (3.7) | -- | 100% | -- | Individual | 3 (32) | 4x4min; 80-90% HR max; Treadmill; 3min recovery. | -- | No | No | No |
| Nilsson et al. [177] | 59.5 (3) | 57 (10.8) | 100% | 88% | Individual | 2 (32) | 3x3min; 85-95% HR peak; Cycle ergometer; 4min recovery. | 45min; 50-60% HR peak; Cycle ergometer. | No | No | No |
| Northey et al. [178] | 60.3 (8.1) | 67.8 (7) | 0% | 0% | -- | 3 (36) | 4-7x 30s; Maximal sprints; Cycle ergometer; 2min recovery. | 30min; 55-65% Watt max; Cycle ergometer. | No | No | No |
| Nybo et al. [179] | 37 (3) | 31 (2) | 100% | 100% | Individual | 3 (36) | 5x2min; 95% HR max; Running; 2min recovery. | 60min; 65% VO2 max; Running. | No | No | No |
| Nytroen et al. [180] | 48 (17) | -- | 67% | -- | Individual | 3 (72) | 4x4min; 85-95% HR max; Treadmill; 3min recovery. | -- | No | No | No |
| Nytroen et al. [181] | 50 (12) | 48 (14) | 76% | 71% | Individual | 2-3 (72) | 4x4min; 85-95% HR peak; Treadmill; 3min recovery. | 40min; 60-80% HR peak; Treadmill. | Yes | No | No |
| Olsen et al. [182] | 62.3 (5.7) | -- | 85% | -- | Individual | 3 (36) | 4-16x 1-4min; 85-90% VO2 peak; Stationary bike; 1-3min recovery. | -- | No | No | No |
| Papadopoulos et al. [183] | 62 (10.4) | -- | 100% | -- | Individual | 2 (16) | 10x60s; 85% HR peak; Recumbent bike; 60s recovery. | -- | No | No | No |
| Pattyn et al. [184] | 57.4 (8.7) | 59.9 (9.2) | 95% | 92% | Individual | 3 (36) | 4x4min; 85-95% HR peak; Cycle ergometer; 3min recovery. | 37min; 70-75% HR peak; Cycle ergometer. | No | No | No |
| Pedersen et al. [185] | 62.3 (5.7) | -- | 84.6% | -- | Individual | 3 (36) | 4-16x 1-4min; 85-90% VO2 peak; Stationary bike; 1-3min recovery. | -- | No | No | No |
| Phillips et al. [186] | 36 (9) | -- | 47.1% | -- | Individual | 3 (18) | 5x60s; 85-105% VO2 max; Cycle ergometer; 1.5min recovery. | -- | No | No | No |
| Piraux et al. [187] | 61 | -- | 83.3% | -- | Individual | 3 (14) | 8-15x 60s; 85% HR max; Cycle ergometer; 60s recovery. | -- | No | No | No |
| Poon et al. [188] | 49.6 (7.8) | 46.5 (3.6) | 100% | 100% | -- | -- | -- | -- | No | No | No |
| Poon et al. [189] | 40.5 (7.1) | 40.1 (3.6) | 100% | 100% | -- | -- | -- | -- | No | No | No |
| Rakobowchuk et al. [190] | 23.1 (2.5) | -- | 35% | -- | Individual | 3 (18) | 20-26x 30s; 120% Watt max; Cycle ergometer; 60s recovery. | -- | No | No | No |
| Reljic et al. [191] | 29.9 (7.2) | 32.8 (8.4) | 33.3% | 20% | Groups | 2 (16) | 2x4min; 85-95% HR max; Cycle ergometer; 2min recovery. | 33min; 65-75% HR max; Cycle ergometer. | No | No | No |
| Reljic et al. [192] | 52.5 | -- | 54% | -- | Individual | 2 (24) | 5x60s; 80-95% HR peak; Cycle ergometer; 60s recovery. | -- | No | No | No |
| Robinson et al. [193] | 52 (10) | 52 (10) | 15% | 21% | Individual | 5 (10) | 4-10x 60s; 85-90% HR peak; Treadmill/Cycle ergometer/Elliptical; 60s recovery. | 20-50min; 60-65% HR peak; Treadmill/Cycle ergometer/Elliptical. | No | No | No |
| Rolid et al. [194] | 50 (12) | 48 (14) | 76% | 71% | Individual | 2-3 (72) | 2-4x 4min; 85-95% HR max; --; 3min recovery. | 25min; 60-80% HR max; --. | Yes | No | No |
| Romain et al. [195] | 29.7 (7.2) | -- | 65.8% | -- | Individual | 2 | 30s intervals; 80-90% HR max; Walking; 1.5min recovery. | -- | No | No | No |
| Rowan et al. [196] | 53.6 (8.2) | 47.7 (6.9) | 27% | 30% | Individual | 3 (36) | 4x4min; 90% HRR; Treadmill; 3min recovery. | 28min; 60-70% HRR; Treadmill. | Yes | No | No |
| Roxburgh et al. [197] | 37.9 (7.1) | 36.5 (9.2) | -- | -- | Individual | 5 (10) | 8-12x 60s; 100% VO2 max; Treadmill; 2.5min recovery. | 30min; 45-60% HRR; Treadmill/Cycle ergometer. | No | No | No |
| Roy et al. [198] | 43.5 (10.2) | -- | 43.3% | -- | -- | -- | -- | -- | -- | -- | -- |
| Ruffino et al. [199] | 55 (5) | -- | 100% | -- | Individual | 3 (48) | 1-2x 10-20s; Maximal sprints; Cycle ergometer; --. | -- | No | Yes | No |
| Rustad et al. [200] | 56 (13) | -- | 67% | -- | Individual | 3 (24) | 4x4min; 85-95% HR peak; Treadmill; 3min recovery. | -- | No | No | No |
| Saanijoki et al. [201] | 48 (5) | 48 (5) | 100% | 100% | Individual | 3 (6) | 4-6x 30s; Maximal sprints; Cycle ergometer; 4min recovery. | 40-60min; 60% Watt max; Cycle ergometer. | No | No | No |
| Safiyari-Hafizi et al. [202] | 57.8 (8.1) | -- | 75% | -- | Individual | 3-7 (55-59) | 5-8x 1-8min; 80-85% VO2 peak; Walking; 1-3min recovery. | -- | Yes | No | No |
| Sargeant et al. [203] | 41 (8) | -- | 100% | -- | Individual | 3 (18) | 4-6x 30s; Maximal sprints; Cycle ergometer; 4.5min recovery. | -- | No | No | No |
| Sawyer et al. [204] | 34.8 (7.7) | 35.6 (8.9) | 44% | 55% | Individual | 3 (24) | 10x60s; 90-95% HR max; Cycle ergometer; 60s recovery. | 30min; 70-75% HR max; Cycle ergometer. | No | No | No |
| Schmitt et al. [205] | 53 (8) | 54 (9) | 0% | 0% | Groups | 3 (8) | 8x60s; 95% HR peak; Walking; 2min recovery. | 75min; 60% HR peak; Walking/Stationary bike. | No | No | Yes |
| Schulz et al. [206] | 51.9 (9.8) | -- | 0% | -- | Individual | 2 (12) | 10x60s; 85-100% VO2 peak; Cycle ergometer; 60s recovery. | -- | Yes | No | No |
| Scott et al. [207] | 30 (3) | -- | 44% | -- | -- | -- | -- | -- | -- | -- | -- |
| Shenouda et al. [208] | 27 (7) | 28 (9) | 100% | 100% | Individual | 1-3 (30-35) | 3x20s; Maximal sprints; Cycle ergometer; 2min recovery. | 45min; 70% HR max; Cycle ergometer. | No | No | No |
| Shepherd et al. [209] | 42 (11) | 43 (11) | 33% | 34% | Groups (10-15) | 3 (30) | 4-12x 15-60s; Maximal sprints; Cycle Ergometer; 45-120s recovery. | 30-45min; 70% HR max; Cycle ergometer. | No | No | No |
| Sim et al. [210] | 31 (8) | 31 (8) | 100% | 100% | Individual | 3 (36) | 15s intervals; 170% VO2 peak; Cycle ergometer; 60s recovery. | 30-45min; 60% VO2 peak; Cycle ergometer. | No | No | No |
| Simonsen et al. [211] | 63.7 (8.1) | -- | 85% | -- | Individual | 2 (24) | 3-4x 4min; 85-95% HR max; Treadmill/Cycle ergometer; 3min recovery. | -- | Yes | No | No |
| Smith-Ryan et al. [212] | 33.2 (12.8) | -- | 0% | -- | Individual | 3 (9) | 10x60s; 90% Watt max; Cycle ergometer; 60s recovery. | -- | No | No | No |
| Smith-Ryan et al. [213] | 44.9 (12.6) | -- | 31% | -- | -- | -- | -- | -- | No | No | No |
| Sogaard et al. [214] | 63 (2) | -- | 50% | -- | Individual | 3 (18) | 3-5x 60s; 85-100% VO2 max; Cycle ergometer; 60s recovery. | -- | No | No | No |
| Stavrinou et al. [215] | 31.5 (3.5) | -- | 42.8% | -- | Individual | 2 (16) | 10x60s; 83% Watt max; Cycle ergometer; 60s recovery. | -- | No | No | No |
| Sveaas et al. [216] | 46.6 (13.6) | -- | 20% | -- | Individual | 2 (24) | 4x4min; 90-95% HR max; Treadmill; 3min recovery. | -- | Yes | No | No |
| Taylor et al. [217] | 65 (7) | 65 (8) | 85% | 83% | Individual | 3 (12) | 4x4min; RPE 15-18 BORG20 RPE; Treadmill/Cycle ergometer/Elliptical; 3min recovery. | 40min; RPE 11-13 BORG20 RPE; Treadmill/Cycle ergometer/Elliptical. | No | No | No |
| Terada et al. [218] | 62 (3) | 63 (5) | 50% | 57.1% | Individual | 4 (48) | 7x60s; 100% VO2 reserve; Treadmill/Cycle ergometer; 3min recovery. | 30-60min; 40% VO2 reserve; Treadmill/Cycle ergometer. | No | Yes | No |
| Tew et al. [219] | 37 (11.1) | 38.5 (13) | 54% | 25% | Individual | 3 (36) | 10x60s; 90% Watt max; Cycle ergometer; 60s recovery. | 30min; 35% Watt max; Cycle ergometer. | No | No | No |
| Tjonna et al. [220] | 55.3 (13.2) | 52 (10.6) | 36.4% | 50% | Individual | 3 (48) | 4x4min; 90% HR max; Treadmill; 3min recovery. | 47min; 70% HR max; Treadmill. | No | No | No |
| Toennesen et al. [221] | 39.4 (12.5) | -- | 55% | -- | Individual | 3 (24) | 10-20x 10s; 90% HR max; Stationary bike; 50s recovery. | -- | No | No | No |
| Tong et al. [222] | 21.3 (1) | -- | 0% | -- | Individual | 3-4 | 4x4min; 90% VO2 max; Cycle ergometer; 3min recovery. | -- | No | No | No |
| Tschentscher et al. [223] | 62.1 (9.5) | 63 (10.9) | 75% | 75% | Individual | 3 (18) | 4x4min; 85-95% HR peak; Cycle ergometer; 3min recovery. | 33min; 65-85% HR peak; Cycle ergometer. | No | No | No |
| Tsirigkakis et al. [224] | 37.2 (9.5) | -- | 100% | -- | Individual | 3 (24) | 48x10s; 100% Watt max; Cycle ergometer; 15s recovery. | -- | No | No | No |
| Turri-Silva et al. [225] | 60.9 (9.7) | -- | 62.5% | -- | Individual | 3 (36) | 4x4min; High-Intensity; Treadmill/Cycle ergometer; 3min recovery. | -- | No | No | No |
| Valent et al. [226] | 39 (12) | -- | 81.9% | -- | -- | -- | -- | -- | No | No | No |
| Vella et al. [227] | 23.1 (6.6) | 28.9 (8.1) | 22.2% | 60% | Individual | 3 (9) | 10x60s; 75-80% HRR; Treadmill/Stationary bike/Elliptical; 60s recovery. | 20mins; 55-59% HRR; Treadmill/Stationary bike/Elliptical. | No | No | No |
| Verbrugghe et al. [228] | 38.5 | -- | 70% | -- | Individual | 2 (12) | 5x60-110s; Maximal sprints; Cycle ergometer; 60s recovery. | -- | Yes | No | No |
| Verbrugghe et al. [229] | 44.3 (8.8) | 44 (11) | 31.5% | 31.5% | Individual | 2 (24) | 5x60-110s; Maximal sprints; Cycle ergometer; 60s recovery. | 14-22min; 60% VO2 max; Cycle ergometer. | Yes | No | No |
| Vestergaard et al. [230] | 42.8 | -- | 87.5% | -- | Individual | 3 (24) | 4x4min; 90% Watt max; FES leg cycle/ski ergometer; 2min recovery. | -- | No | No | No |
| Vidal-Almela et al. [231] | 58 (9) | -- | 71% | -- | Groups | 2 (20) | 4x4min; 85-95% HR peak; Treadmill/Cycle ergometer/Elliptical; 3min recovery. | -- | No | No | No |
| Way et al. [232] | 57.4 (9.5) | -- | 67% | -- | Groups | 2 (20) | 4x4min; 85-95% HR peak; Treadmill/Cycle ergometer/Elliptical; 3min recovery. | -- | No | No | No |
| Weng et al. [233] | 22.3 (0.2) | 22.5 (1) | 100% | 100% | Individual | 5 (25) | 5x3min; 80% VO2 max; Cycle ergometer; 3min recovery. | 30min; 60% VO2 max; Cycle ergometer. | No | No | No |
| Willoughby et al. [234] | 22.9 (3.1) | -- | 41.2% | -- | Individual | 3 (12) | 4-6x 30s; Maximal sprints; Treadmill; 4min recovery. | -- | No | No | No |
| Wilson et al. [235] | 52 (2) | -- | 64% | -- | Individual | 3 (36) | 3-10x 1-3min; 90% HR peak; Cycle ergometer; 1-2min recovery. | -- | No | No | No |
| Winding et al. [236] | 54 (6) | 58 (8) | 53.8% | 58.3% | Individual | 3 (33) | 10x60s; 95% Watt max; Cycle ergometer; 60s recovery. | 40min; 50% Watt max; Cycle ergometer. | No | No | No |
| Wormgoor et al. [237] | 52.2 (7.1) | 52.5 (7) | 100% | 100% | Individual | 3 (36) | 1. 12x60s; 95% Watt max; Cycle ergometer; 60s recovery.  2. 8x30s; Maximal sprints; Cycle ergometer; 2.25min recovery. | 17.5-32min; 55% Watt max; Cycle ergometer. | Yes | No | No |
| Zhang et al. [238] | 21.5 (1.7) | 20.9 (1.4) | 0% | 0% | Individual | 3-4 (44) | 4x4min; 90% VO2 max; Cycle ergometer; 3min recovery. | 300 kJ; 60% VO2 max; Cycle ergometer. | No | No | No |
| Zisko et al. [239] | 39 (5) | 40 (7) | 100% | 100% | Individual | 3 (18) | 1x4min; 90-95% HR max; Treadmill; --. | 47min; 70% HR max; Treadmill. | No | No | No |

*Notes*. FITT descriptions follow the convention of duration of intervals/exercise, intensity of exercise sessions, modality/type of exercise, and for HIIT interventions, recovery duration. HR: heart rate; HRR: heart rate reserve; VO2: volume of oxygen consumption; PPO: peak power output; BORG10 RPE: rating of perceived exertion based on the 0-10 scale; BORG20 RPE: rating of perceived exertion based on the 6-20 Borg scale.
