# Supplementary material for: Rates of compliance and adherence to high-intensity interval training: a systematic review and Meta-analyses
Source: Int J Behav Nutr Phys Act. 2023 Nov 21;20:134. doi: 10.1186/s12966-023-01535-w (PMC10664287; doi:10.1186/s12966-023-01535-w)
Supplement: Supplementary file 8 — Additional File 8. Table including information on compliance rates for supervised interventions, such as the method of measurement, unit of measurement, and mean compliance (SD). [file 12966_2023_1535_MOESM8_ESM.docx]

**Additional File 8.** Compliance Rates to Supervised Interventions

| **Study Reference** | **Method of Measurement** | **Unit of Measurement** | **# of Dropouts (%)** | | **Compliance Result (SD)** | |
| --- | --- | --- | --- | --- | --- | --- |
|  | | | **HIIT** | **MICT** | **HIIT** | **MICT** |
| Aamot et al. [54] | Attendance to Supervised Sessions | Percentage of attendance | 5 (14.7%) | -- | 100% | -- |
| Adams et al. [55] | Attendance to Supervised Sessions | Percentage of attendance | 0 (0%) | -- | 99% | -- |
| Allen et al. [56] | Attendance to Supervised Sessions | Percentage of attendance | 1 (4.8%) | -- | 95% (8%) | -- |
| Allen et al. [57] | Attendance to Supervised Sessions | Percentage of attendance | 5 (41.7%) | 4 (44.4%) | 52% (9%) | 49% (13%) |
| Allison et al. [58] | Attendance to Supervised Sessions | Percentage of attendance | 0 (0%) | -- | 99% (3%) | -- |
| Alvarez et al. [59] | Attendance to Supervised Sessions | Percentage of attendance | 0 (0%) | -- | 89% (5%) | -- |
| Arad et al. [60] | Attendance to Supervised Sessions | Percentage of attendance | 5 (35.7%) | -- | 100% | -- |
| Archila et al. [61] | Attendance to Supervised Sessions | Percentage of attendance | 2 (18.2%) | -- | 100% | -- |
| Astorino et al. [62] | Attendance to Supervised Sessions | Percentage of attendance | 1 (9.1%) | -- | 96.4% (2.4%) | -- |
| Atan et al. [63] | Attendance to Supervised Sessions | Percentage of attendance | 0 (0%) | 0 (0%) | 98.6% (2.4%) | 95.5% (3.8%) |
| Avila-Gandi­a et al. [64] | Attendance to Supervised Sessions | Percentage of attendance | 10 (50%) | -- | 85.5% (5.5%) | -- |
| Baekkerud et al. [65] | Attendance to Supervised Sessions | Percentage of attendance | 4 (33.3%) | 0 (0%) | 100% | 100% |
| Bang-Kittilsen et al. [66] | Attendance to Supervised Sessions | Percentage of attendance | 9 (20.9%) | -- | 65% (29.2%) | -- |
| Banitalebi et al. [67] | Attendance to Supervised Sessions | Percentage of attendance | 0 (0%) | 0 (0%) | 78% | 82% |
| Beetham et al. [68] | Attendance to Supervised Sessions | Percentage of attendance | 3 (25%) | 4 (44.4%) | 91.7% (19.4%) | 93.1% (9.2%) |
| Benda et al. [69] | Attendance to Supervised Sessions | Percentage of attendance | 2 (16.7%) | 2 (16.7%) | 100% | 100% |
| Benham et al. [70] | Attendance to Supervised Sessions | Percentage of attendance | 3 (18.8%) | 3 (21.4%) | 65% (25.2%) | 81% (21.5%) |
| Berger et al. [71] | Attendance to Supervised Sessions | Percentage of attendance | 0 (0%) | 0 (0%) | 100% | 100% |
| Billany et al. [72] | Attendance to Supervised Sessions | Percentage of attendance | 2 (25%) | 2 (25%) | 92% | 92% |
| Briggs et al. [74] | Attendance to Supervised Sessions | Percentage of attendance | 2 (15.4%) | -- | 91% (8.5%) | -- |
| Brobakken et al. [75] | Attendance to Supervised Sessions | Percentage of attendance | 0 (0%) | -- | 71% | -- |
| Cano-Montoya et al. [76] | Attendance to Supervised Sessions | Percentage of attendance | 0 (0%) | -- | 60.9% | -- |
| Cerini et al. [77] | Attendance to Supervised Sessions | Percentage of attendance | 3 (21.4%) | 0 (0%) | 94% (17%) | 96% (5.9%) |
| Cheema et al. [78] | Attendance to Supervised Sessions | Percentage of attendance | 0 (0%) | 2 (33%) | 79% (15%) | 55% (43%) |
| Ciolac et al. [79] | Attendance to Supervised Sessions | Percentage of attendance | 5 (31.3%) | 5 (31.3%) | 83.2% (6.3%) | 83.7% (3.9%) |
| Coletta et al. [80] | Attendance to Supervised Sessions | Percentage of attendance | 4 (25%) | 4 (28.6%) | 90% | 89% |
| Connolly et al. [81] | Attendance to Supervised Sessions | Percentage of attendance | 0 (0%) | -- | 97.8% (2.2%) | -- |
| Conraads et al. [82] | Attendance to Supervised Sessions | Percentage of attendance | 15 (15%) | 11 (11%) | 99.2% (3.1%) | 98.9% (4.2%) |
| Cooke et al. [83] | Attendance to Supervised Sessions | Percentage of attendance | 1 (6.3%) | -- | 88% | -- |
| Cooper et al. [84] | Attendance to Supervised Sessions | Percentage of attendance | 1 (6.7%) | 0 (0%) | 91.4% (7.6%) | 90.9% (7.3%) |
| Currie et al. [85] | Attendance to Supervised Sessions | Percentage of attendance | 0 (0%) | 0 (0%) | 79.2% (16.7%) | 91.7% (12.5%) |
| Currie et al. [86] | Attendance to Supervised Sessions | Percentage of attendance | 0 (0%) | 0 (0%) | 89.6% (10.4%) | 91.7% (8.3%) |
| D’Amuri et al. [87] | Attendance to Supervised Sessions | Percentage of attendance | 6 (27.3%) | 6 (27.3%) | 97% (15.3%) | 95% (15.3%) |
| Damme et al. [88] | Attendance to Supervised Sessions | Percentage of attendance | 5 (27.8%) | -- | 76.3% (15.8%) | -- |
| Deraas et al. [89] | Attendance to Supervised Sessions | Percentage of attendance | 1 (6.3%) | -- | 70% (20.4%) | -- |
| Devin et al. [90] | Attendance to Supervised Sessions | Percentage of attendance | 1 (3.3%) | 1 (5.9%) | 97% (18.3%) | 97.1% (12.1%) |
| Devin et al. [91] | Attendance to Supervised Sessions | Percentage of attendance | 1 (5.6%) | 2 (10.5%) | 99.3% (2.2%) | 100% |
| Dissing et al. [92] | Attendance to Supervised Sessions | Percentage of attendance | 0 (0%) | -- | 100% | -- |
| Dolan et al. [93] | Attendance to Supervised Sessions | Percentage of attendance | 0 (0%) | 1 (8.3%) | 98.7% | 98.6% |
| Dowd et al. [94] | Attendance to Supervised Sessions | Percentage of attendance | 6 (30%) | -- | 74.3% (1.5%) | -- |
| Egegaard et al. [95] | Attendance to Supervised Sessions | Percentage of attendance | 0 (0%) | -- | 90% (11.6%) | -- |
| Eichner et al. [96] | Attendance to Supervised Sessions | Percentage of attendance | 0 (0%) | 0 (0%) | 97.1% (3.9%) | 95.9% (7.6%) |
| Ellingsen et al. [97] | Attendance to Supervised Sessions | Percentage of attendance | 11 (12.5%) | 11 (14.1%) | 97% | 97% |
| Elmer et al. [98] | Attendance to Supervised Sessions | Percentage of attendance | 1 (14.3%) | 1 (14.3%) | 100% | 100% |
| Emtner et al. [99] | Attendance to Supervised Sessions | Percentage of attendance | 4 (15.4%) | -- | 100% | -- |
| Emtner et al. [100] | Attendance to Supervised Sessions | Percentage of attendance | 0 (0%) | -- | 100% | -- |
| Flaherty et al. [101] | Attendance to Supervised Sessions | Percentage of attendance | 0 (0%) | -- | 100% | -- |
| Flemmen et al. [102] | Attendance to Supervised Sessions | Percentage of attendance | 3 (25%) | -- | 91.7% (4.2%) | -- |
| Foster et al. [103] | Attendance to Supervised Sessions | Percentage of attendance | 3 (12.5%) | 1 (5%) | 100% | 100% |
| Francois et al. [104] | Attendance to Supervised Sessions | Percentage of attendance | 2 (11.1%) | -- | 100% | -- |
| Freese et al. [105] | Attendance to Supervised Sessions | Percentage of attendance | 12 (32.4%) | -- | 100% | -- |
| Freitag et al. [106] | Attendance to Supervised Sessions | Percentage of attendance | -- | -- | 100% | -- |
| Freyssin et al. [107] | Attendance to Supervised Sessions | Percentage of attendance | 0 (0%) | 0 (0%) | 100% | 100% |
| Gilbertson et al. [109] | Attendance to Supervised Sessions | Percentage of attendance | 11 (64.7%) | 3 (25%) | 89.6% (2.1%) | 91.7% (8.3%) |
| Gildea et al. [110] | Attendance to Supervised Sessions | Percentage of attendance | 3 (30%) | 2 (15.4%) | 94% (6%) | 96% (6%) |
| Gillen et al. [111] | Attendance to Supervised Sessions | Percentage of attendance | 0 (0%) | -- | 100% | -- |
| Gillen et al. [112] | Attendance to Supervised Sessions | Percentage of attendance | 1 (10%) | 1 (9.1%) | 93.9% (3%) | 97% (6.1%) |
| Gloeckl et al. [113] | Attendance to Supervised Sessions | Percentage of attendance | 5 (14.3%) | 6 (16.7%) | 82.8% (10.6%) | 81.7% (8.3%) |
| Golightly et al. [114] | Attendance to Supervised Sessions | Percentage of attendance | 8 (27.6%) | -- | 97% (5%) | -- |
| Gorostegi-Anduaga et al. [115] | Attendance to Supervised Sessions | Percentage of attendance | 2 (4.5%) | 2 (4.8%) | 100% | 100% |
| Grace et al. [116] | Attendance to Supervised Sessions | Percentage of attendance | 3 (12%) | -- | 100% | -- |
| Gremeaux et al. [117] | Attendance to Supervised Sessions | Percentage of attendance | 4 (10.1%) | -- | 97% | -- |
| Guillamo et al. [118] | Attendance to Supervised Sessions | Percentage of attendance | 0 (0%) | -- | 77.5% | -- |
| Haines et al. [119] | Attendance to Supervised Sessions | Percentage of attendance | 2 (28.6%) | -- | 72.4% (43.3%) | -- |
| Hatle et al. [120] | Attendance to Supervised Sessions | Percentage of attendance | 0 (0%) | -- | 95% | -- |
| Hearon et al. [121] | Attendance to Supervised Sessions | Percentage of attendance | 9 (40.9%) | -- | 90% (14.1%) | -- |
| Heggelund et al. [122] | Attendance to Supervised Sessions | Percentage of attendance | 4 (25%) | -- | 85% (9%) | -- |
| Heje et al. [123] | Attendance to Supervised Sessions | Percentage of attendance | 1 (20%) | -- | 95.4% (12.1%) | -- |
| Hettchen et al. [125] | Attendance to Supervised Sessions | Percentage of attendance | 0 (0%) | -- | 78% (12%) | -- |
| Heydari et al. [126] | Attendance to Supervised Sessions | Percentage of attendance | 0 (0%) | -- | 100% | -- |
| Higgins et al. [127] | Attendance to Supervised Sessions | Percentage of attendance | 5 (17.9%) | 2 (6.5%) | 83.3% (8.3%) | 87.8% (10.6%) |
| Hindso et al. [128] | Attendance to Supervised Sessions | Percentage of attendance | 0 (0%) | -- | 100% | -- |
| Humphreys et al. [130] | Attendance to Supervised Sessions | Percentage of attendance | 1 (9.1%) | -- | 87.5% (25%) | -- |
| Hwang et al. [131] | Attendance to Supervised Sessions | Percentage of attendance | 2 (15.4%) | -- | 71.2% | -- |
| Hwang et al. [132] | Attendance to Supervised Sessions | Percentage of attendance | 2 (11.8%) | 4 (22.2%) | 88% | 88% |
| Iellamo et al. [133] | Attendance to Supervised Sessions | Percentage of attendance | 2 (20%) | 2 (20%) | 100% | 100% |
| Izadi et al. [135] | Attendance to Supervised Sessions | Percentage of attendance | 7 (31.8%) | -- | 68% | -- |
| Jabbour et al. [136] | Attendance to Supervised Sessions | Percentage of attendance | 0 (0%) | -- | 100% | -- |
| Jabbour et al. [137] | Attendance to Supervised Sessions | Percentage of attendance | 0 (0%) | -- | 100% | -- |
| Jakobsen et al. [138] | Attendance to Supervised Sessions | Percentage of attendance | 1 (11.1%) | 2 (16.6%) | 66.7% | 83.9% |
| Jung et al. [139] | Attendance to Supervised Sessions | Percentage of attendance | 0 (0%) | 1 (5.9%) | 100% | 100% |
| Jung et al. [26] | Attendance to Supervised Sessions | Percentage of attendance | 12 (25.5%) | 15 (28.8%) | 100% | 100% |
| Kang et al. [140] | Attendance to Supervised Sessions | Percentage of attendance | 2 (7.7%) | -- | 96% | -- |
| Karlsen et al. [141] | Attendance to Supervised Sessions | Percentage of attendance | 2 (20%) | -- | 98.7% (2.3%) | -- |
| Kaur et al. [143] | Attendance to Supervised Sessions | Percentage of attendance | 5 (15.6%) | -- | 85.2% | -- |
| Keating et al. [144] | Attendance to Supervised Sessions | Percentage of attendance | 2 (15.4%) | 2 (15.4%) | 96% | 92% |
| Keating et al. [145] | Attendance to Supervised Sessions | Percentage of attendance | -- | -- | 95.8% (8.1%) | -- |
| Kemmler et al. [146] | Attendance to Supervised Sessions | Percentage of attendance | 7 (17.5%) | 7 (17.9%) | 82.6% (11.1%) | 82.3% (12%) |
| Keteyian et al. [148] | Attendance to Supervised Sessions | Percentage of attendance | 4 (19%) | 3 (16.7%) | 96.7% (3.3%) | 96.7% (6.7%) |
| Keytsman et al. [149] | Attendance to Supervised Sessions | Percentage of attendance | 5 (21.7%) | -- | 95% | -- |
| Kiel et al. [150] | Attendance to Supervised Sessions | Number of sessions per week | 0 (0%) | -- | 2 (1) | -- |
| Klonizakis et al. [151] | Attendance to Supervised Sessions | Percentage of attendance | 1 (8.3%) | 3 (30%) | 90.3% (28.3%) | 71.7% (46%) |
| Knowles et al. [152] | Attendance to Supervised Sessions | Percentage of attendance | 3 (12%) | -- | 100% | -- |
| Kong et al. [153] | Attendance to Supervised Sessions | Percentage of attendance | 2 (13.3%) | 3 (18.8%) | 100% | 100% |
| Lanzi et al. [154] | Attendance to Supervised Sessions | Percentage of attendance | 1 (10%) | 0 (0%) | 98.6% (4.2%) | 100% |
| Lee et al. [155] | Attendance to Supervised Sessions | Percentage of attendance | 3 (20%) | -- | 67% (20%) | -- |
| Lee et al. [156] | Attendance to Supervised Sessions | Percentage of attendance | 3 (20%) | -- | 82.3% (8.8%) | -- |
| Locke et al. [157] | Attendance to Supervised Sessions | Percentage of attendance | 6 (40%) | 1 (5.9%) | 100% | 100% |
| Lunt et al. [25] | Attendance to Supervised Sessions | Percentage of attendance | 7 (43.8%) | 3 (17.6%) | 59% | 75% |
| Lyall et al. [158] | Attendance to Supervised Sessions | Percentage of attendance | 4 (12.9%) | -- | 99% (3%) | -- |
| MacDonald et al. [159] | Attendance to Supervised Sessions | Percentage of attendance | 1 (9.1%) | -- | 99% (3.6%) | -- |
| MacLean et al. [160] | Attendance to Supervised Sessions | Percentage of attendance | 3 (25%) | -- | 100% | -- |
| Madsen et al. [161] | Attendance to Supervised Sessions | Percentage of attendance | 1 (9%) | -- | 100% | -- |
| Madssen et al. [162] | Attendance to Supervised Sessions | Percentage of attendance | 4 (21%) | 1 (4.5%) | 90% | 90% |
| Madssen et al. [163] | Attendance to Supervised Sessions | Percentage of attendance | 0 (0%) | -- | 97.5% (5.4%) | -- |
| Martin et al. [164] | Number of Adherent Participants | Percentage of adherent participants | 6 (30%) | -- | 70% | -- |
| Martins et al. [165] | Attendance to Supervised Sessions | Percentage of attendance | 3 (18.8%) | 1 (7.1%) | 100% | 100% |
| Matsuo et al. [166] | Attendance to Supervised Sessions | Percentage of attendance | 0 (0%) | 0 (0%) | 97.6% (4.3%) | 95.9% (7.3%) |
| Mendelson et al. [167] | Attendance to Supervised Sessions | Percentage of attendance | 2 (10%) | 1 (5%) | 98% (2%) | 98% (2%) |
| Metcalfe et al. [168] | Attendance to Supervised Sessions | Percentage of attendance | 0 (0%) | -- | 97% (6.2%) | -- |
| Metcalfe et al. [169] | Attendance to Supervised Sessions | Percentage of attendance | 15 (30%) | -- | 99% (4.1%) | -- |
| Metcalfe et al. [170] | Attendance to Supervised Sessions | Percentage of attendance | 3 (18.8%) | -- | 92% (10%) | -- |
| Midtgaard et al. [171] | Attendance to Supervised Sessions | Percentage of attendance | 35 (32.4%) | -- | 66.6% | -- |
| Mijwel et al. [172] | Attendance to Supervised Sessions | Percentage of attendance | 9 (12%) | -- | 68% (27%) | -- |
| Moholdt et al. [173] | Attendance to Supervised Sessions | Percentage of attendance | 4 (12.1%) | 5 (13.9%) | 94% (7.6%) | 95.5% (6.2%) |
| Munk et al. [174] | Attendance to Supervised Sessions | Percentage of attendance | 0 (0%) | -- | 90% | -- |
| Nikseresht et al. [176] | Attendance to Supervised Sessions | Percentage of attendance | 5 (33.3%) | -- | 90.6% (5.3%) | -- |
| Nilsson et al. [177] | Attendance to Supervised Sessions | Percentage of attendance | 3 (50%) | 3 (37.5%) | 100% | 100% |
| Northey et al. [178] | Attendance to Supervised Sessions | Percentage of attendance | 0 (0%) | 0 (0%) | 78.7% (13.2%) | 79.4% (12%) |
| Nybo et al. [179] | Attendance to Supervised Sessions | Number of sessions per week | -- | -- | 2.0 (0.1) | 2.5 (0.2) |
| Nytroen et al. [180] | Attendance to Supervised Sessions | Percentage of attendance | 2 (7.7%) | -- | 95.8% (8.3%) | -- |
| Nytroen et al. [181] | Attendance to Supervised Sessions | Percentage of attendance | 2 (5.4%) | 1 (2.4%) | 81% (30.6%) | 81% (30.6%) |
| Olsen et al. [182] | Attendance to Supervised Sessions | Percentage of attendance | -- | -- | 81% | -- |
| Papadopoulos et al. [183] | Attendance to Supervised Sessions | Percentage of attendance | 0 (0%) | -- | 95% (5.2%) | -- |
| Pedersen et al. [185] | Attendance to Supervised Sessions | Percentage of attendance | 0 (0%) | -- | 74% | -- |
| Phillips et al. [186] | Physical Activity Energy Expenditure | MET min/week | 11 (6.7%) | -- | 1.5 METs (0.34 METs) | -- |
| Piraux et al. [187] | Attendance to Supervised Sessions | Percentage of attendance | 0 (0%) | -- | 92% (13.1%) | -- |
| Rakobowchuk et al. [190] | Attendance to Supervised Sessions | Percentage of attendance | 0 (0%) | -- | 100% | -- |
| Reljic et al. [191] | Attendance to Supervised Sessions | Percentage of attendance | 2 (16.7%) | 3 (30%) | 81% (21%) | 79% (23%) |
| Reljic et al. [192] | Attendance to Supervised Sessions | Percentage of attendance | 1 (7.7%) |  | 92.5% (9.7%) | -- |
| Robinson et al. [193] | Attendance to Supervised Sessions | Percentage of attendance | 0 (0%) | 0 (0%) | 100% | 100% |
| Rolid et al. [194] | Attendance to Supervised Sessions | Percentage of attendance | 2 (5.1%) | 1 (2.4%) | 80.6% (30.6%) | 80.6% (30.6%) |
| Romain et al. [195] | Attendance to Supervised Sessions | Percentage of attendance | 19 (50%) | -- | 64% (38.7%) | -- |
| Rowan et al. [196] | Attendance to Supervised Sessions | Percentage of attendance | 0 (0%) | 2 (18%) | 100% | 100% |
| Roxburgh et al. [197] | Attendance to Supervised Sessions | Percentage of attendance | -- | -- | 100% | 86.7% (5.9%) |
| Ruffino et al. [199] | Attendance to Supervised Sessions | Percentage of attendance | -- | -- | 99% (2%) | -- |
| Rustad et al. [200] | Attendance to Supervised Sessions | Percentage of attendance | -- | -- | 95.8% (8.3%) | -- |
| Saanijoki et al. [201] | Attendance to Supervised Sessions | Percentage of attendance | 1 (7%) | 1 (7%) | 100% | 97.6% |
| Safiyari-Hafizi et al. [202] | Attendance to Supervised Sessions | Percentage of attendance | 6 (30%) | -- | 77% (20%) | -- |
| Sargeant et al. [203] | Attendance to Supervised Sessions | Percentage of attendance | 0 (0%) | -- | 100% | -- |
| Sawyer et al. [204] | Attendance to Supervised Sessions | Percentage of attendance | 2 (18%) | 2 (18%) | 100% | 100% |
| Schmitt et al. [205] | Exercise Minutes Completed in Supervised Sessions | Number of minutes | 0 (0%) | 0 (0%) | 239min (17min) | 413min (32min) |
| Schulz et al. [206] | Attendance to Supervised Sessions | Percentage of attendance | 0 (0%) | -- | 97.2% (5.1%) | -- |
| Shenouda et al. [208] | Attendance to Supervised Sessions | Percentage of attendance | 1 (10%) | 1 (11.1%) | 88.6% (2.9%) | 91.4% (5.7%) |
| Shepherd et al. [209] | Attendance to Supervised Sessions | Percentage of attendance | 0 (0%) | 0 (0%) | 83% (14%) | 61% (15%) |
| Sim et al. [210] | Attendance to Supervised Sessions | Percentage of attendance | 0 (0%) | -- | 98% (3%) | 97% (4%) |
| Simonsen et al. [211] | Attendance to Supervised Sessions | Percentage of attendance | 3 (23.1%) | -- | 69.2% | -- |
| Smith-Ryan et al. [212] | Attendance to Supervised Sessions | Percentage of attendance | 0 (0%) | -- | 100% | -- |
| Sogaard et al. [214] | Attendance to Supervised Sessions | Percentage of attendance | 6 (21.4%) | -- | 98.7% | -- |
| Stavrinou et al. [215] | MVPA minutes in Supervised Sessions | MVPA minutes per week | 0 (0%) | -- | 1012min (505min) | -- |
| Sveaas et al. [216] | Attendance to Supervised Sessions | Percentage of attendance | 2 (15.4%) | -- | 80% | -- |
| Taylor et al. [217] | Attendance to Supervised Sessions | Percentage of attendance | 2 (4.3%) | 3 (6.4%) | 100% | 90.6% |
| Terada et al. [218] | Attendance to Supervised Sessions | Percentage of attendance | 0 (0%) | 0 (0%) | 97.2% (2.7%) | 97.3% (3.7%) |
| Tew et al. [219] | Attendance to Supervised Sessions | Percentage of attendance | 1 (7.7%) | 0 (0%) | 62% (37.6%) | 75% (14.1%) |
| Tjonna et al. [220] | Attendance to Supervised Sessions | Percentage of attendance | 1 (9%) | 1 (12.5%) | 90% (2%) | 90% (2%) |
| Toennesen et al. [221] | Attendance to Supervised Sessions | Percentage of attendance | 7 (19.4%) | -- | 91.3% | -- |
| Tong et al. [222] | Attendance to Supervised Sessions | Percentage of attendance | 2 (11.1%) | -- | 95% (2%) | -- |
| Tschentscher et al. [223] | Attendance to Supervised Sessions | Percentage of attendance | 3 (15%) | 2 (10%) | 99.2% | 99.2% |
| Tsirigkakis et al. [224] | Attendance to Supervised Sessions | Percentage of attendance | 2 (20%) | -- | 95% (4%) | -- |
| Turri-Silva et al. [225] | Attendance to Supervised Sessions | Percentage of attendance | 1 (11.1%) | -- | 93.5% | -- |
| Verbrugghe et al. [228] | Attendance to Supervised Sessions | Percentage of attendance | 0 (0%) | -- | 100% | -- |
| Verbrugghe et al. [229] | Attendance to Supervised Sessions | Percentage of attendance | 1 (5%) | 2 (10.5%) | 93.3% (5.4%) | 97.5% (5.4%) |
| Vestergaard et al. [230] | Attendance to Supervised Sessions | Percentage of attendance | 1 (12.5%) | -- | 82.1% (24%) | -- |
| Vidal-Almela et al. [231] | Attendance to Supervised Sessions | Percentage of attendance | -- | -- | 86.2% (25.5%) | -- |
| Way et al. [232] | Attendance to Supervised Sessions | Percentage of attendance | -- | -- | 80% (25%) | -- |
| Weng et al. [233] | Attendance to Supervised Sessions | Percentage of attendance | -- | -- | 100% | 100% |
| Willoughby et al. [234] | Attendance to Supervised Sessions | Percentage of attendance | 3 (17.6%) | -- | 100% | -- |
| Wilson et al. [235] | Attendance to Supervised Sessions | Percentage of attendance | -- | -- | 78% (4%) | -- |
| Winding et al. [236] | Attendance to Supervised Sessions | Percentage of attendance | 0 (0%) | 0 (0%) | 91% (18%) | 94% (9%) |
| Wormgoor et al. [237] | Attendance to Supervised Sessions | Percentage of attendance | -- | -- | 91.2% (9.9%) | 90.4% (6.8%) |
| Zhang et al. [238] | Attendance to Supervised Sessions | Percentage of attendance | 1 (6.3%) | 1 (6.3%) | 96% (3%) | 95% (1%) |
| Zisko et al. [239] | Attendance to Supervised Sessions | Percentage of attendance | 0 (0%) | 0 (0%) | 95% | 95% |

*Notes*. Standard deviations of compliance rates of 100% that were included in the meta-analysis were changed from 0% to 0.001%. MET: metabolic equivalent; MVPA: moderate-to-vigorous physical activity.
